# Dendritic cell redundancy enables priming of anti-tumor CD4^+^ T cells in pancreatic cancer

**DOI:** 10.1016/j.ccell.2026.04.005

**Published:** 2026-05-07

**Authors:** Courtney T.S. Kureshi, Michael J. Walsh, Rakeeb Kureshi, Victoire Cardot-Ruffino, Dennis A. Agardy, Lestat R. Ali, James M. Dougan, Li Qiang, Jiao Shen, Chong Zuo, Patrick J. Lenehan, S. Jennifer Wang, Eugena Chang, Joshua Remland, Lauren Brais, Thomas E. Clancy, James M. Cleary, Jason L. Hornick, Brandon M. Huffman, Joseph D. Mancias, George Molina, Mark Fairweather, Jonathan A. Nowak, Kimberly J. Perez, Douglas A. Rubinson, Sarah Slater, Ritchell van Dams, Jiping Wang, Brian M. Wolpin, Lei Zhao, Katharine Barrientos, Ruslan Novosiadly, Miranda Broz, Harshabad Singh, Michael Dougan, Stephanie K. Dougan

**Affiliations:** 1 Harvard Medical School Program in Immunology, Boston, MA, USA; 2 Massachusetts General Hospital, Department of Medicine, Division of Gastroenterology, Boston, MA, USA; 3 Dana-Farber Cancer Institute, Department of Cancer Immunology & Virology, Boston, MA, USA; 4 Harvard Medical School Program in Virology, Boston, MA, USA; 5 Harvard Medical School, Boston, MA, USA; 6 Brookline High School, Brookline, MA, USA; 7 Dana-Farber Cancer Institute, Department of Medical Oncology, Boston, MA, USA; 8 Brigham and Women’s Hospital, Department of Pathology, Boston, MA, USA; 9 Dana-Farber Cancer Institute, Department of Oncologic Pathology, Boston, MA, USA; 10 Dana-Farber Cancer Institute, Department of Radiation Oncology, Boston, MA, USA; 11 Brigham and Women’s Hospital, Division of Surgical Oncology, Boston, MA, USA; 12 Bristol Myers Squibb, Princeton, NJ, USA; 13 Lead contact

## Abstract

Pancreatic ductal adenocarcinoma (PDAC) is resistant to current immunotherapies and lacks effective antitumor CD8^+^ T cells, which is potentially due to insufficient cross-presentation by cDC1s. Here, we combine a STING agonist with anti-CTLA-4 and anti-PD-1 to achieve durable remissions and immunologic memory in multiple mouse models of poorly immunogenic PDAC. We find that tumor control does not depend on CD8^+^ T cells or tumor cell MHC expression but instead requires IFNγ-producing CD4^+^ T cells (Th1s) that are primed by dendritic cells in lymph nodes. The triple combination immunotherapy induces an accumulation of activated cDC2s carrying tumor antigen into tumor-draining lymph nodes; cDC2s are required for orthotopic tumor clearance. Intratumoral CD4^+^ T cells and cDC2s remain present in treatment-naive and chemotherapy-exposed human PDAC. In chemotherapy-exposed patients’ blood, cDC2s outnumber cDC1s by 10-fold. Therefore, therapeutic targeting of the cDC2-CD4^+^ T cell-IFNγ axis could be efficacious in PDAC.

## In brief

Kureshi et al. show that combining an innate immune agonist and immune checkpoint blockade effectively treats pancreatic tumors in mouse models. Tumor control depends on cDC2-primed CD4^+^ T cells and IFNγ. Human PDAC retains cDC2s and CD4^+^ T cells after chemotherapy, suggesting a potential therapeutic approach for PDAC.

## Graphical abstract

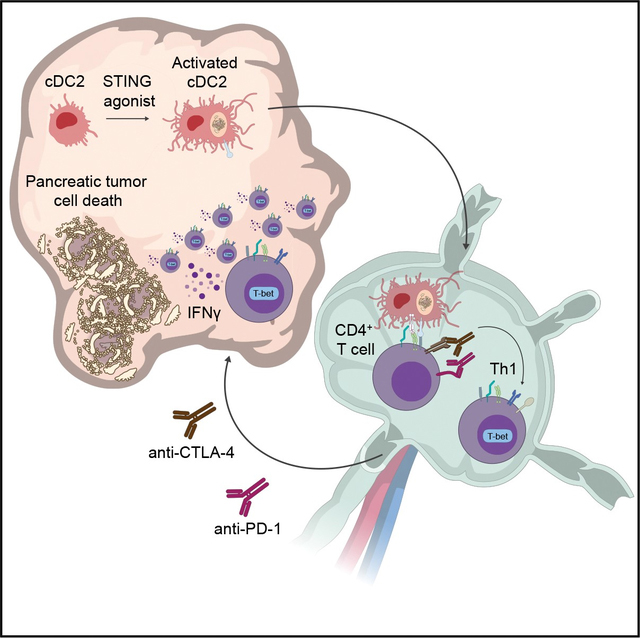

## INTRODUCTION

Pancreatic ductal adenocarcinoma (PDAC) remains one of the most intractable cancers, with a 13% five-year survival rate.^1^ PDAC cells produce myeloid cell-recruiting chemokines and growth factors, such as CXCL1 and GM-CSF, which establish a highly immunosuppressive tumor microenvironment^2,3^ and impact systemic metabolism.^4^ Although CD8^+^ T cells exist in most human PDAC, immune checkpoint blockade has only been effective in the approximately 1% of tumors with mismatch repair machinery defects (MMRd).^5–8^

Most current cancer immunotherapy approaches target CD8^+^ T cells, which can kill tumor cells directly and produce cytokines, such as interferon gamma (IFNγ) and tumor necrosis factor alpha (TNFα).^9,10^ In tumors, though, CD8^+^ T cells cannot fully execute their effector functions due to exhaustion. Anti-PD-1 (programmed cell death protein 1) therapy promotes renewal of TCF1^+^ CD8^+^ T cells, allowing a continuous supply of functional CD8^+^ T cells to the tumor.^11^ The mechanisms of anti-CTLA-4 (cytotoxic T-lymphocyte-associated protein 4) therapy include blocking CTLA-4 from competing with CD28 for CD80/86 costimulation. In mice, anti-CTLA-4 also depletes regulatory T cells (Tregs).^12^ Immune checkpoint blockade depends upon tumor cell-expressed major histocompatibility complex (MHC)-I; tumor mutations driving both primary and acquired resistance to checkpoint blockade converge upon MHC-I presentation pathways.^13–15^

CD4^+^ T cells help orchestrate immune responses. They recognize tumor antigens and produce cytokines, such as IFNγ and IL-2, to activate CD8^+^ T cells and sustain their proliferation.^16–18^ CD4^+^ T cells can also directly kill tumor cells that express MHC-II.^19–21^ Yet, most tumors do not express MHC-II, and CD4^+^ T cells thus primarily encounter tumor antigen presented by MHC-II on dendritic cells and macrophages.^22–24^ CD4^+^ T cells differentiate into effector subsets, which have distinct functions in PDAC; in mouse models, Th1 and Th2 cells are associated with positive outcomes, while Th17 cells are largely tumor-promoting.^25,26^ Effector Th1s produce copious IFNγ, which diffuses widely within the tumor microenvironment, activating macrophages and natural killer (NK) cells, and inducing tumor cytostasis and ischemia.^10,27–29^ CD4^+^ T cell help is essential for optimal CD8^+^ T cell tumor infiltration, cytotoxicity, and memory.^30–36^ Tumor vaccines inducing CD4^+^ T cell responses have demonstrated clinical benefit in melanoma, renal cell carcinoma, and other cancers.^37,38^

Classical dendritic cells (cDCs) prime naive T cells, and dendritic cells also present antigen and provide costimulation to effector T cells. cDC1s cross-present tumor antigen and activate CD8^+^ and CD4^+^ T cells. cDC1s are also importantly licensed by CD4^+^ T cells in cancer, and CD4^+^ T cell-dendritic cell interactions shape the response to immune checkpoint blockade.^39,40^ cDC2s less competently cross-present antigen, but cDC2s and other innate immune cells can present tumor antigen via unconventional mechanisms to CD8^+^ T cells.^39,41,42^ cDC2s from renal tumors can cross-present antigen to autologous CD8^+^ T cells *ex vivo*.^43^ Upon Treg depletion, cDC2s prime CD4^+^ T cells in B16 melanoma.^44^ In patients with pancreatic cancer, higher cDC2 abundance is associated with improved prognosis,^45^ and there are fewer cDC1s in pancreatic cancer models than there are in lung cancer models driven by the same oncogenes.^46^ Both cDC1s and cDC2s are relatively rare in pancreatic tumors, and a mechanistic role for cDC2s in poorly immunogenic tumors is unclear.^26,47^ Indeed, though emerging evidence suggests cDC2s^′^ potential role in immunogenic tumors, the dearth of appropriate antigen-presenting cells in poorly immunogenic tumors impedes immunotherapy’s success.

Innate immune agonists activate dendritic cells, augmenting antigen presentation, and T cell priming. Agonism of the cyclic GMP-AMP synthase/stimulator of interferon genes (cGAS/STING) DNA sensing pathway produces a strong type I IFN response, which can enhance T cell-innate immune cell interactions in human tumors.^36,48–50^ In preclinical models, STING agonists induce anti-tumor NK cell and CD8^+^ T cell responses and can repress metastasis in a tumor cell-intrinsic manner.^51–55^

Here, we show that the addition of an STING agonist to dual immune checkpoint blockade cures mice of poorly immunogenic, immune checkpoint blockade-refractory pancreatic cancer. We find that cDC2s prime IFNγ-producing CD4^+^ T cells (Th1s) to control tumors. CD8^+^ T cells and cDC1s are dispensable, as are tumor cell MHC-I expression and IFNγ sensing. In patients with PDAC, CD4^+^ T cells remain present, and cDC2s remain more abundant than cDC1s after chemotherapy treatment. Our results highlight that the addition of an innate immune agonist to existing T cell-directed therapies can transform immunotherapy-unresponsive tumors into responsive tumors. Importantly, they also show the critical ability of cDC2-primed CD4^+^ T cells to eradicate tumors that are unresponsive to CD8^+^ T cell killing.

## RESULTS

### Localized STING agonist delivery, combined with systemic anti-PD-1 and anti-CTLA-4, controls otherwise immune checkpoint blockade-resistant pancreatic tumors, including β2m−/− tumors

**Figure 1.**
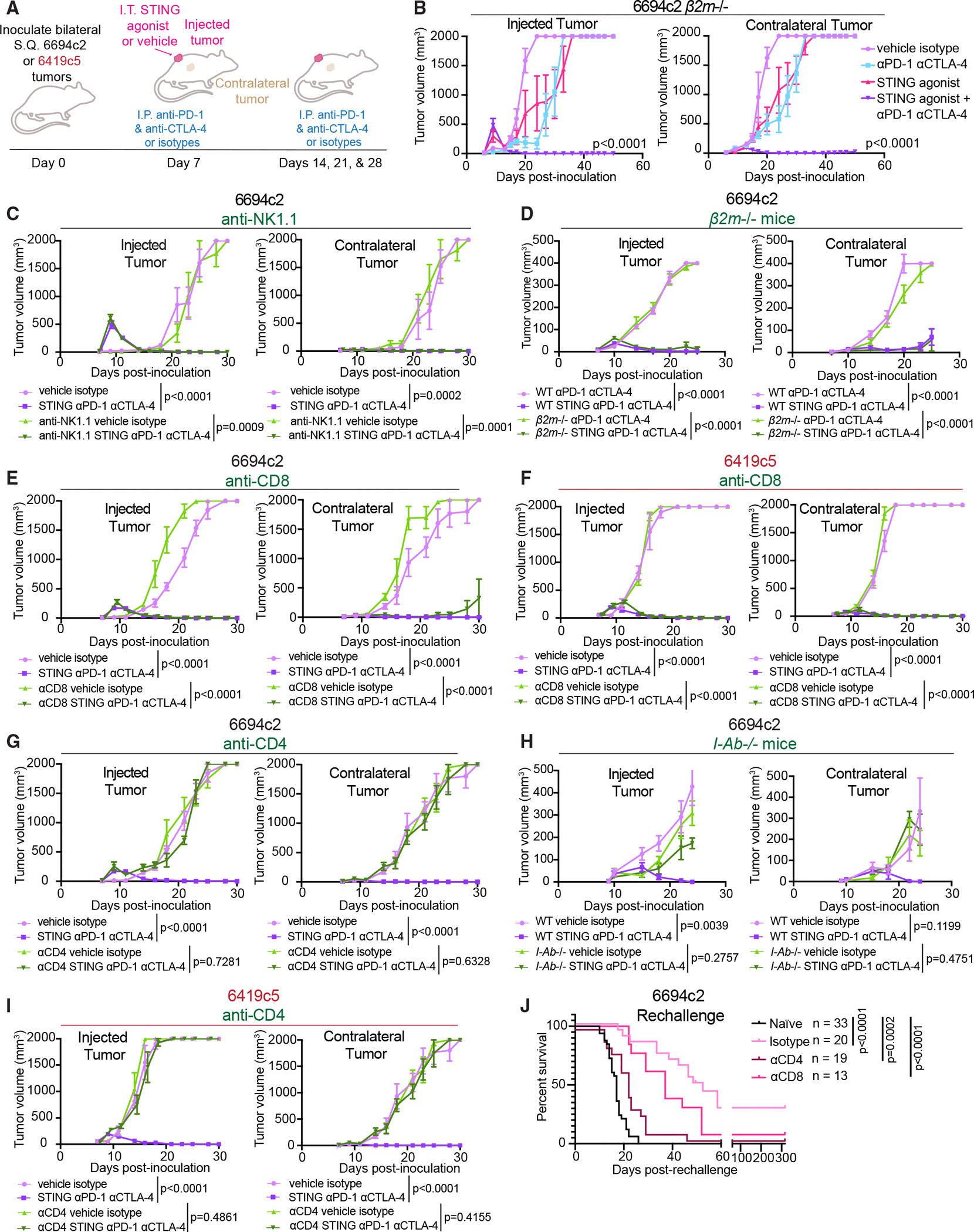
The addition of an adjuvant to anti-PD-1 and anti-CTLA-4 combination therapy confers CD4^+^ T cell-dependent tumor regression and memory in pancreatic cancer (A–I) Diagram of experiments shown in (B–I). Mice were inoculated with bilateral subcutaneous (B) 6694c2 *β2m*−/−, (C–E, G, and H) 6694c2, or (F and I) 6419c5 tumors. Seven days later, one tumor was injected with STING agonist or vehicle. On the same day and weekly thereafter, 10 mg/kg anti-PD-1 and 10 mg/kg anti-CTLA-4 (or isotypes) were administered intraperitoneally. Tumor growth was measured every 2–3 days. *N* = 5 in all groups except (D), WT STING anti-PD-1 anti-CTLA-4 (*N* = 10). (J) Survival curve of mice previously cured of 6694c2 WT tumors for >35 days that were rechallenged with 6694c2 tumors. Mice received two doses of 200 μg αCD8, αCD4, or isotype prior to rechallenge. Tumor growth curve statistical significance determined by calculating area under the curve (AUC) in GraphPad Prism and conducting *t* tests of areas (error bars report standard error of the mean [SEM]). Kaplan Meier statistical significance determined via the Log rank (Mantel-Cox) test and Bonferroni correction (Bonferroni-corrected α = 0.017). Illustration from NIAID NIH BioArt Source: bioart.niaid.nih.gov/bioart/591. See also Figures S1 and S2; Table S1.

Both primary and acquired resistance to immune checkpoint blockade can result from loss of MHC-I presentation of tumor antigens on the tumor cell surface.^13–15^ We sought to test whether the addition of an immune adjuvant, such as an STING agonist, could overcome the need for tumor cell-expressed MHC-I. Mice were inoculated with bilateral subcutaneous 6694c2 β-2 microglobulin (*β2m*)−/− PDAC tumors and treated with anti-PD-1 and anti-CTLA-4, a STING agonist, or all three (triple combination therapy).^2^ Immune checkpoint blockade therapy was delivered systemically, while the STING agonist was delivered intratumorally into one of the subcutaneous tumors, and tumor growth was measured in both tumors (Figure 1A). Consistent with previous reports of this model, neither checkpoint blockade nor the STING agonist alone effectively controlled tumor growth.^2,56,57^ However, the triple combination therapy induced complete tumor regressions on both sides of the mice (Figures 1B; Table S1). 6694c2 tumors were also cured when treatment was delayed until 14 days post-inoculation, when injected tumors averaged 200 mm^3^ (Figure S1A). We further evaluated STING agonist therapy in a spontaneously metastatic PDAC model (6694c2-met).^58^ 6694c2-met cells were implanted prior to surgical removal. Most control mice developed metastases after thirty-five days; when the primary tumor was injected with the STING agonist, zero mice developed metastases (Figures S1B–S1D).

### PDAC tumor cells are refractory to direct effects of STING agonism

Prior to investigating immune mechanisms of tumor rejection, we verified that the STING agonist was not itself toxic to tumor cells. *In vitro* treatment with high concentrations of the compound did not affect tumor growth kinetics (Figure S1E). To assess tumor cell response to the STING agonist, RNA sequencing was performed on 6694c2 PDAC and B16F10 melanoma cells treated *in vitro*. B16F10 cells upregulated interferon-response genes after STING agonist treatment, but 6694c2 PDAC cells showed minimal response to STING agonist treatment. This suggested that tumor regression following STING agonist treatment is not due to direct tumor cell-intrinsic effects (Figure S1F).

### CD4^+^ T cells are required for pancreatic tumor regression

The profound *in vivo* tumor control led us to investigate the immunologic mechanism of rejection. NK cells have been implicated in rejection of MHC-I-deficient tumors, but NK cells were not required for tumor rejection here (Figure 1C). Recombination activating 2 (*Rag2*)−/− and *Tcrα*−/− mice were inoculated with 6694c2 tumors, and mice treated with triple combination therapy failed to reject their tumors. Adaptive immunity, and specifically αβ T cells, were required (Figures S2A and S2B). μMT−/− mice rejected their tumors, indicating that B cells were not required (Figure S2C).^59,60^ Mice treated with anti-CD8 depleting antibody and *β2m*−/− mice (which lack conventional CD8^+^ T cells) rejected tumors following treatment; CD8^+^ T cells were dispensable for tumor rejection in multiple poorly immunogenic pancreatic tumor models (6694c2 and 6419c5) (Figures 1D–1F). Perforin-1, TNFα, and tumor cell-expressed caspase-8 were not required for tumor regression (Figures S2D–S2F, Data S1). Collectively, these data indicated that neither CD8^+^ T cells nor their cytolytic functions, including extrinsic death receptor activation, were necessary for tumor regressions.

By contrast, tumors in mice lacking CD4^+^ T cells did not respond to therapy, demonstrating that CD4^+^ T cells were required for tumor rejection in multiple pancreatic tumor models (Figures 1G–1I). To determine if MHC-II expressed on the tumor cell surface was required for tumor rejection, we generated 6694c2 class II transactivator (*Ciita)*−/− tumor cells to eliminate IFN-inducible MHC-II expression. *Ciita*−/− tumors were rejected by triple combination therapy, indicating that CD4^+^ T cells did not kill tumor cells via direct recognition of tumor cell-expressed MHC-II (Figure S2G).

Given the requirement for CD4^+^ T cells during the primary antitumor response, we investigated their role during the memory response. To do so, we rechallenged mice that were previously cured of 6694c2 tumors. These mice were protected from rechallenge, indicating immunologic memory. Although CD8^+^ T cell depletion modestly impaired protection, CD4^+^ T cell depletion abrogated memory to 6694c2 cells, indicating that CD4^+^ T cells were necessary for protection against rechallenge (Figure 1J). Similarly, rechallenge of mice that had cleared 6694c2 *β2m*−/− cells showed that mice survived even when depleted of CD8^+^ T cells, although that defense was lost when mice were additionally depleted of CD4^+^ T cells (Figure S2H).

**Figure 2.**
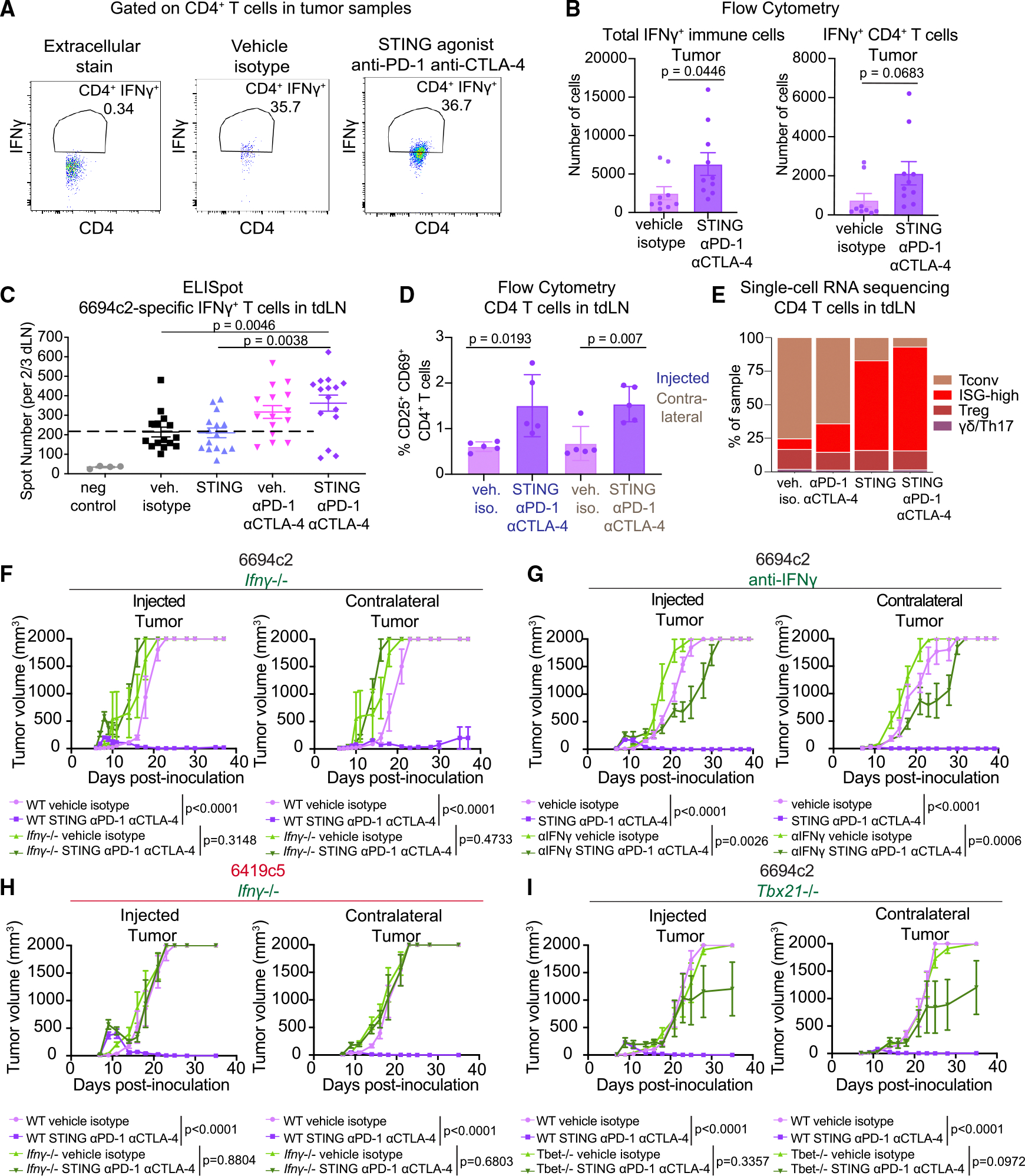
Anti-tumor immunity depends on IFNγ and the Th1 transcription factor T-bet (A). Representative flow cytometry plots from the experiment shown in (B). 6694c2-bearing mice were treated as described in Figure 1A. On day 15 post-inoculation, (B) tumors and (C) Tumor-draining lymph nodes (tdLNs) were harvested for analysis. (B) Total intratumoral IFNγ^+^ CD45^+^ cells and IFNγ^+^ CD4^+^ T cells. *N* = 5 mice, *N* = 10 tumors. (C) ELISpot was conducted at the midpoint of tumor growth. Lymphocytes from treated mice were plated with 6694c2 cells. *N* = 5. (D) CD25^+^ CD69^+^ CD4^+^ T cells as a percent of total CD4^+^ T cells. tdLNs were harvested on day 10 post-inoculation. *N* = 5 mice. (E) Relative frequences of CD4^+^ T cell subsets in tdLNs two days after triple combination therapy (day 9 post-inoculation) by scRNA-seq (Figure S3). (F–I) *In vivo* tumor growth curves from (F, G, and I) 6694c2-and (H) 6419c5-bearing *Ifnγ*−/−, *Tbx21*−/−, and WT mice. *N* = 5 mice per group, except in *Ifnγ*−/− vehicle isotype, where *N* = 4. Tumor growth curve statistical significance determined by calculating AUC in GraphPad Prism and conducting *t* tests of areas (error bars report SEM). Bar plot statistical significance determined via *t* tests (error bars report standard deviation [SD]). See also Figures S2–S4 and Table S1.

We next examined CD4^+^ T cell cytokine production during the anti-tumor immune response. Triple combination therapy increased the total number of IFNγ^+^ cells in mid-stage tumors, including IFNγ^+^ CD4^+^ T cells (Figures 2A and 2B). ELISpot analysis of lymphocytes from tumor-draining lymph nodes cocultured with 6694c2 cells indicated that T cells from both checkpoint blockade- and triple combination therapy-treated mice elicited increased IFNγ production in response to tumor antigens compared to T cells from control mice (Figure 2C). T cell activation occurred early; three days after triple combination therapy, there was a higher proportion of activated CD4^+^ T cells in triple combination-treated groups than there was in control groups in lymph nodes both ipsilateral and contralateral to the STING agonist-injected tumor, indicating a systemic CD4^+^ T cell response (Figure 2D).

To better characterize global immune cell changes upon combination therapy, we performed single-cell RNA sequencing (scRNA-seq) of tumor-draining lymph nodes 48 h post-treatment (Figure S3). Samples were sorted into ZsGreen^+^ and ZsGreen^−^ immune cells to capture tumor antigen uptake (Figures S3A–S3C). Lymph nodes of mice treated with STING agonist alone or with triple combination therapy were enriched for interferon-activated CD4^+^ T cells, dendritic cells, and CD8^+^ T cells (Figures S3D and S3E). Subclustering of CD4^+^ T cells revealed clusters of T_conv_, ISG-high T cells, Tregs, Th17s, and γδ T cells (Figures 2E and S3F). Fractions of ISG-high CD4^+^ T cells increased in STING agonist-treated mice compared to vehicle-treated mice, with an accompanying compensatory decrease in the fraction of T_conv_ (Figure 2E). Treatment did not appear to change the fraction of Tregs recovered (Figure 2E).

### IFNγ and Th1s are required for pancreatic tumor control

IFNγ produced in the tumor can spread throughout the tumor stroma, inducing tumor cell cytostasis.^27,61,62^ To determine whether IFNγ was responsible for tumor control, tumor growth was compared in WT and *Ifnγ*−/− mice. *Ifnγ*−/− mice were entirely unable to control tumors after triple combination therapy, and this finding was replicated in the 6419c5 tumor model and in WT mice treated with IFNγ blocking antibody (Figures 2F–2H). Th1s, differentiated CD4^+^ T cells that produce IFNγ, express and depend on the transcription factor T-bet (*Tbx21*).^63^ Therefore, we compared tumor control in *Tbx21*−/− mice to that in WT mice and found that *Tbx21*−/− mice lost tumor control, which phenocopied IFNγ- and CD4^+^ T cell-deficient models (Figure 2I).

Because IFNγ can induce cytostasis, we tested whether IFNγ affected 6694c2 cell growth *in vitro*. B16F10 cells grew slower in response to IFNγ in a dose-dependent manner, whereas 6694c2 cells maintained normal growth kinetics (Figure S4A). To determine whether IFNγ acted directly on the tumor cell *in vivo*, we generated *Ifnγr1*-deficient tumor cells. 6694c2 *Ifnγr1*−/− tumors were controlled by triple combination therapy; tumor-intrinsic IFNγ signaling was not required (Figure S4B).

IFNγ can activate other immune cells to clear pathogens and tumors.^10^ *In vivo* tumor growth experiments were conducted in ΔΔ*Gata1* mice (which lack eosinophils), WT mice treated with anti-Ly6G (depleted of neutrophils), *Mr1*−/− γδ−/− mice (which lack MAIT cells and γδ T cells), and inducible nitric oxide synthase (iNOS)−/− mice (which lack iNOS-mediated killing by immune cells, including macrophages and neutrophils) (Figures S4C–S4F). All models controlled tumor growth (Figures S4C–S4F).^64^ Therefore, eosinophils, neutrophils, mucosal-associated invariant T (MAIT) cells, γδ T cells, and iNOS were all not uniquely required for tumor rejection.

### Tumor-controlling CD4^+^ T cells are primed in the tumor-draining lymph node by activated, antigen-containing cDC2s

**Figure 3.**
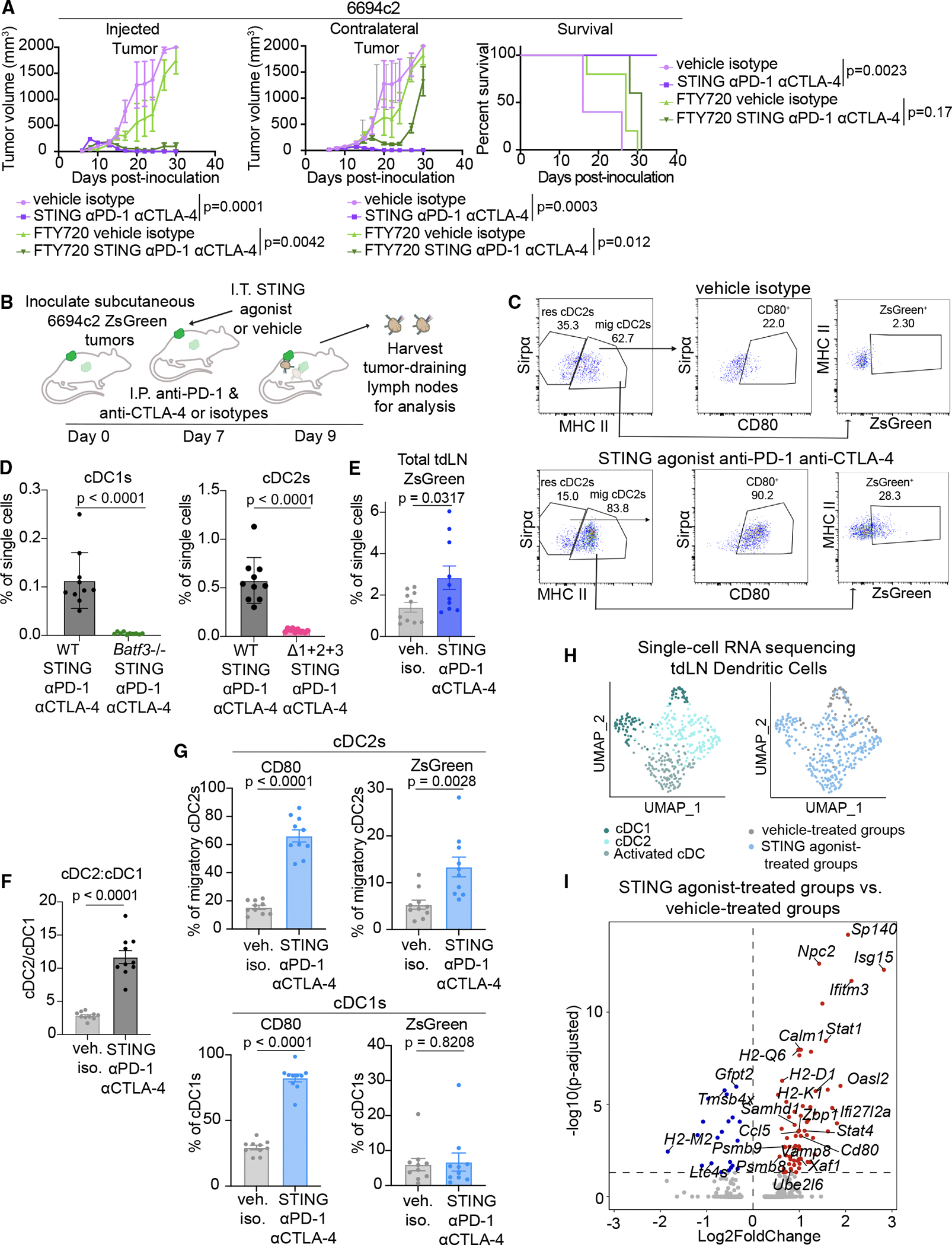
In the draining lymph node, CD4^+^ T cells are primed, and activated, antigen-containing cDC2s accumulate (A) *In vivo* tumor growth curves from 6694c2-bearing mice treated with FTY720 (green) or water (purple). Mice were treated as described in Figure 1A. Mice were bled to verify FTY720 function (Figure S5, Data S1). *N* = 5 per group. (B) Diagram of experiments in panels C–I. In brief, tdLNs from 6694c2-bearing mice were harvested two days post-triple combination treatment (day 9 post-inoculation) for (C–G) flow cytometry or (H and I) sorting and scRNA-seq. (C) Representative flow cytometry plots of cDC2s from vehicle-treated (top) and triple combination therapy-treated (bottom) mice. Populations were previously gated on single cells → F4/80^−^ Ly6C^−^ → CD11c^+^ → Sirpα^+^ XCR1^−^ cells (Figure S6). (D) Frequency of dendritic cell subsets as a percent of single cells from mice treated with triple combination therapy. cDC1s are shown in WT and *Batf3*−/− mice and cDC2s are shown in WT and Δ1+2+3 mice. Data are shown to confirm that gating captured cDC1s and cDC2s. (E) Total tdLN ZsGreen^+^ cells in vehicle-treated and triple combination therapy-treated WT mice, as a percent of single cells. (F) cDC2:cDC1 ratio in WT vehicle-treated and triple combination-treated mice. (G) Fractions of CD80^+^ and ZsGreen^+^ cDC2s (top) and cDC1s (bottom) out of total MHC-II^+^ cDC2s and cDC1s, respectively. (H) UMAP of total dendritic cells recovered from tdLNs on day 9 post-inoculation. Left UMAP shows dendritic cell subsets, and right UMAP shows cell recovery by treatment condition. (I) Differentially expressed genes between dendritic cells from STING agonist-treated and vehicle-treated groups (Figure S3). *N* = 5 mice unless otherwise stated. Tumor growth curve statistical significance determined by calculating AUC in GraphPad Prism and conducting *t* tests of areas (error bars report SEM). Kaplan Meier statistical significance determined via Log rank (Mantel-Cox) test. Bar plot significance determined via *t* tests (error bars report SD). Illustrations from NIAID NIH BioArt Source: bioart.niaid.nih.gov/bioart/591; bioart.niaid.nih.gov/bioart/304. See also Figures S5–S8 and Table S1.

Local priming of tumor-specific T cells by intratumoral dendritic cells has been suggested.^65^ To determine whether T cell activation in the tumor-draining lymph node was necessary for tumor control, mice were treated with FTY720, a sphingosine-1-phosphate receptor antagonist that blocks lymphocyte egress from lymph nodes (Figure S5; Data S1). Therapeutic efficacy was abolished in the contralateral tumor upon FTY720 treatment, confirming that T cell priming in lymph nodes is required for systemic CD4^+^ T cell tumor clearance, though perhaps local priming can compensate at the treated tumor site (Figure 3A).

cDC1s are often required for anti-tumor immune responses because of their superior ability to cross-present antigen to CD8^+^ T cells.^41,66,67^ Since CD8^+^ T cells were dispensable for pancreatic tumor rejection, we set out to find the antigen-presenting cell responsible for CD4^+^ T cell priming; we investigated which dendritic cell populations had adequate tumor antigen uptake and costimulatory molecule expression. Two days after treatment, tumor-draining lymph nodes were harvested from mice bearing ZsGreen^+^ tumors, and dendritic cell populations were analyzed via flow cytometry (Figure 3B; Figure S6A). Activated (CD80^+^), antigen-bearing (ZsGreen^+^), MHC-II^+^ cDC2s accumulated in tumor-draining lymph nodes of treated mice (Figures 3C–3G). cDC1s were lost in *Batf3*−/− mice and cDC2s were lost in Δ1+2+3 mice, thereby confirming correct identification of these subsets (Figure 3D; Figures S6B–S6E).^68^ Total tumor antigen was increased in tumor-draining lymph nodes of treated mice (Figure 3E). A high fraction of both cDC1s and cDC2s expressed CD80 after triple combination treatment, but only cDC2s contained increased tumor antigen. Activated, antigen-containing cDC2s were found in lymph nodes draining both the injected tumor and the contralateral tumor, suggesting that intratumorally injected STING agonist leads to systemic exposure. These data indicated that cDC2s have increased potential to activate antigen-specific T cells due to both greater numbers and enhanced tumor antigen uptake (Figures 3F and 3G).

We also examined tumor-draining lymph node dendritic cells via scRNA-seq. Dendritic cells expressed the highest interferon-stimulated gene (ISG) signatures (Figure S3D). Subclustering dendritic cells revealed cDC1s, cDC2s, and a cluster containing activated cDC1s and activated cDC2s (Figure 3H). cDC2s and activated dendritic cells were found primarily in STING agonist-treated mice (Figure 3H). Tumor-draining lymph nodes from STING agonist-treated mice were enriched in activated dendritic cells at the expense of cDC1s (Figures S3G and S3H). Comparing dendritic cells from STING agonist-treated mice to those from control-treated mice indicated that STING agonism strongly induced interferon-response genes (e.g., *Isg15*, *Ifitm3*, and *Cd80*) (Figure 3I).

In *Ccr2* (CC chemokine receptor 2)−/− mice, the same cDC2 accumulation, increase in costimulatory molecules, and tumor antigen uptake was seen, suggesting that the cells we identified as cDC2s were not monocytes/macrophages (Figure S7A). cDC2s can resemble macrophages via surface marker expression, so we examined tumor-draining lymph node macrophage populations after triple combination therapy (Figures S7B–S7D). F4/80^+^ macrophage frequencies remained consistent. Although F4/80^+^ macrophages expressed the costimulatory molecule CD80, there was no change in tumor antigen uptake (Figure S7B). In contrast, following triple combination therapy, we found fewer F4/80^−^ macrophages, and these cells had no changes in costimulatory molecule expression or tumor antigen uptake (Figure S7C). Subcapsular sinus macrophages strikingly disappeared from tumor-draining lymph nodes of treated mice, indicating that they were not responsible for anti-tumor CD4^+^ T cell priming (Figure S7D; Data S1).

Flow cytometry characterization of tumor-draining lymph node macrophages did not implicate macrophages as important antigen-presenting cells. Nevertheless, we evaluated the efficacy of triple combination therapy in the following monocyte- and macrophage-deficient mouse models: mice lacking monocyte-derived macrophages (*Ccr2*−/− mice and WT mice treated with anti-Ly6C), mice depleted of tissue-resident macrophages (WT mice treated with anti-CSF1R), and mice lacking both monocyte-derived and tissue-resident macrophages (*Ccr2*−/− mice treated with anti-CSF1R) (Figures S7E–S7H). In all four models of monocyte and/or macrophage deficiency, triple combination therapy was effective; we could not demonstrate that macrophages were required for tumor rejection.

### Type I IFN is not required for the cDC2 response or for therapeutic efficacy

STING agonism induces a strong type I IFN response.^48,49^ In our model, serum IFNα levels peaked 4 h after treatment and rapidly diminished thereafter (Figure S8A). To understand whether cDC2 activation and enrichment was due to increased type I IFN levels, tumor-draining lymph nodes from *Ifnar1*−/− mice were analyzed 48 h after triple combination treatment. The cDC2:cDC1 ratio and fraction of activated cDC2s remained highly elevated in *Ifnar1*−/− mice (Figure S8B). *Ifnar1*−/− mice responded to triple combination therapy, indicating no requirement for type I interferon signaling (Figures S8C and S8D). There was no tumor cell-intrinsic effect of type I IFN on 6694c2 tumor growth *in vitro* or *in vivo* (Figures S8E and S8F). Therefore, although treated mice had elevated serum type I IFN levels, type I IFN was not essential for either tumor control or cDC2 accumulation in tumor-draining lymph nodes. Examination of tumor-draining lymph nodes in *Ifnγ*−/− mice revealed that, although IFNγ was essential for tumor clearance, it was not essential for cDC2 accumulation and activation; cDC2 activation was upstream of IFNγ (Figure S8G).

### Orthotopic pancreatic tumors are controlled in a CD4^+^ T cell- and IFNγ-dependent manner, and activated cDC2s accumulate after triple combination therapy

**Figure 4.**
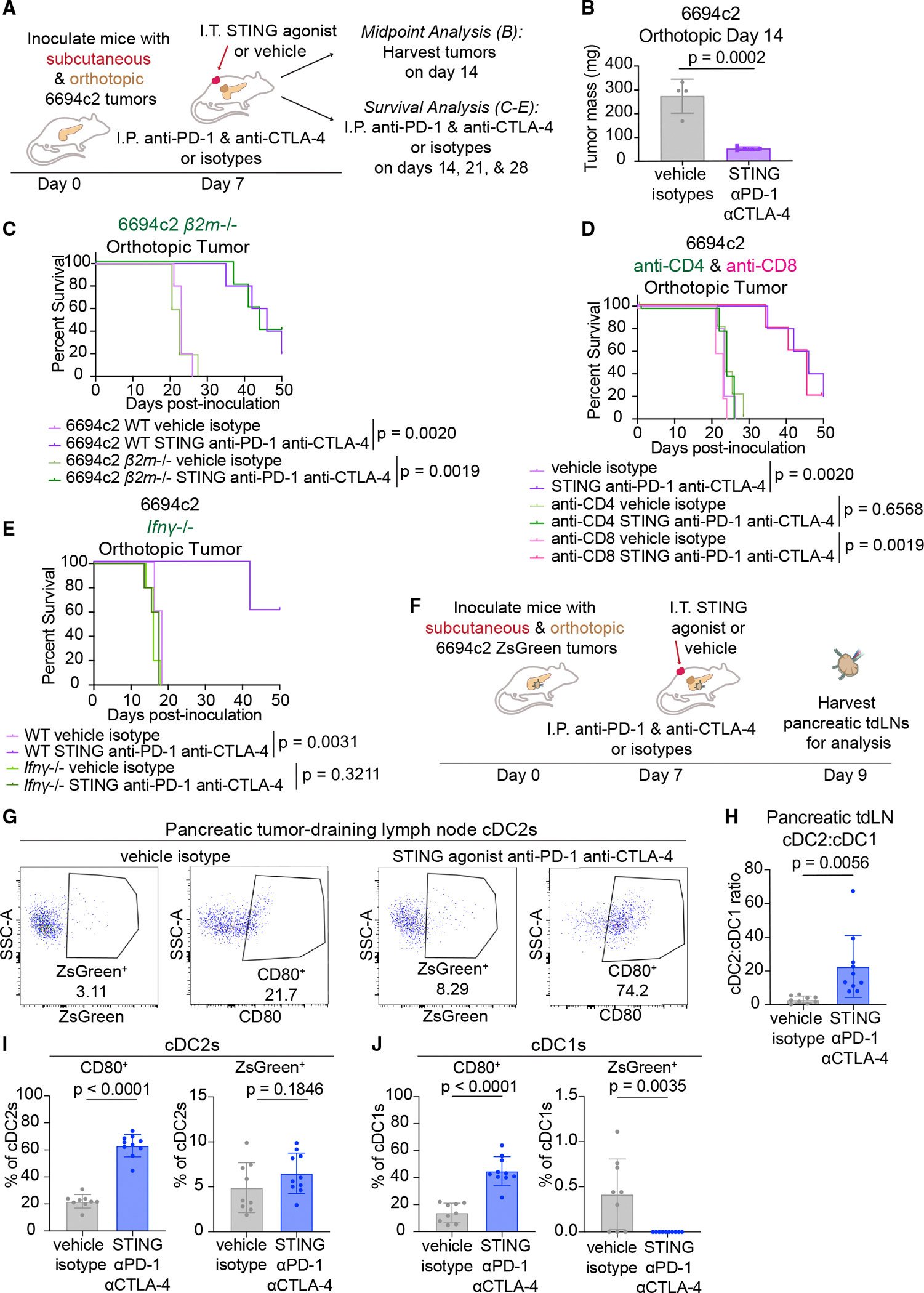
CD4^+^ T cells and IFNγ are required for clearance of orthotopic pancreatic tumors, and there is an accumulation of activated, antigen-containing cDC2s in pancreatic tumor-draining lymph nodes (A) Diagram of experiments in (B–E). Mice were inoculated with one orthotopic and one subcutaneous 6694c2 tumor. Seven days later, the subcutaneous tumor was injected with STING agonist or vehicle, and mice received IP anti-PD-1 and anti-CTLA-4 or isotypes. Anti-PD-1 and anti-CTLA-4 was repeated weekly for four doses. (B) Tumor mass of day 14 orthotopic tumors. (C–E) Mice were treated as described in (A) and survival measured. (F) Experimental design diagram for experiments shown in (G–J). Experiment was conducted as in (A–E), except mice were inoculated with 6694c2 ZsGreen tumors, and pancreatic tdLNs were harvested two days post-triple combination therapy. (G) Representative flow cytometry plots of cDC2s from pancreatic tdLNs (see Data S1). (H) cDC2-to-cDC1 ratio in pancreatic tdLNs in control and triple combination therapy-treated mice. (I and J) Fractions of CD80^+^ and ZsGreen^+^ (I) cDC2s and (J) cDC1s in pancreatic tdLNs in control and triple combination therapy-treated mice. Kaplan-Meier (panels B–E) statistical significance was determined via the Log rank (Mantel-Cox) test. Bar graph statistical significance determined via *t* tests (error bars report SD). *N* = 5 mice except (F–J) (*N* = 9 vehicle & *N* = 10 STING αPD-1 αCTLA-4). Illustrations from NIAID NIH BioArt Source: bioart.niaid.nih.gov/bioart/591; bioart.niaid.nih.gov/bioart/304; and https://bioart.niaid.nih.gov/bioart/239.

To better represent the human PDAC immune microenvironment, we examined treatment efficacy in mice bearing orthotopic pancreatic tumors (Figure 4). Mice were also inoculated with one subcutaneous tumor to mimic a distant metastasis. Seven days later, the subcutaneous tumor was injected with the STING agonist, and checkpoint blockade was delivered systemically (Figure 4A). Orthotopic tumors from triple combination therapy-treated mice were significantly smaller than control tumors (Figure 4B). Triple combination therapy conferred a significant survival benefit to mice bearing orthotopic 6694c2 WT or 6694c2 *β2m*−/− tumors (Figure 4C). Orthotopic tumor control and survival benefit was abolished upon CD4^+^ T cell depletion, but not upon CD8^+^ T cell depletion, indicating a reliance upon CD4^+^, but not CD8^+^, T cells (Figure 4D). *Ifnγ*−/− mice experienced no survival benefit from triple combination therapy, indicating that IFNγ was required (Figure 4E). Therefore, the CD4^+^ T cell- and IFNγ-dependent axis of immune control found in the subcutaneous setting was replicated in the orthotopic setting.

We next examined dendritic cell population changes in pancreatic-draining lymph nodes 48 h post-triple combination treatment (Figure 4F). Like in subcutaneous tumor-draining lymph nodes, the cDC2-to-cDC1 ratio and cDC2 activation increased in pancreatic tumor-draining lymph nodes after triple combination treatment (Figures 4G–4J, Data S1). Notably, cDC2s were the only dendritic cells containing tumor antigen (Figures 4I and 4J). Therefore, in tumors growing in the pancreas, cDC2s can be activated to deliver tumor antigens to pancreatic draining lymph nodes.

### cDC2s prime CD4^+^ T cells and are required for orthotopic tumor control

**Figure 5.**
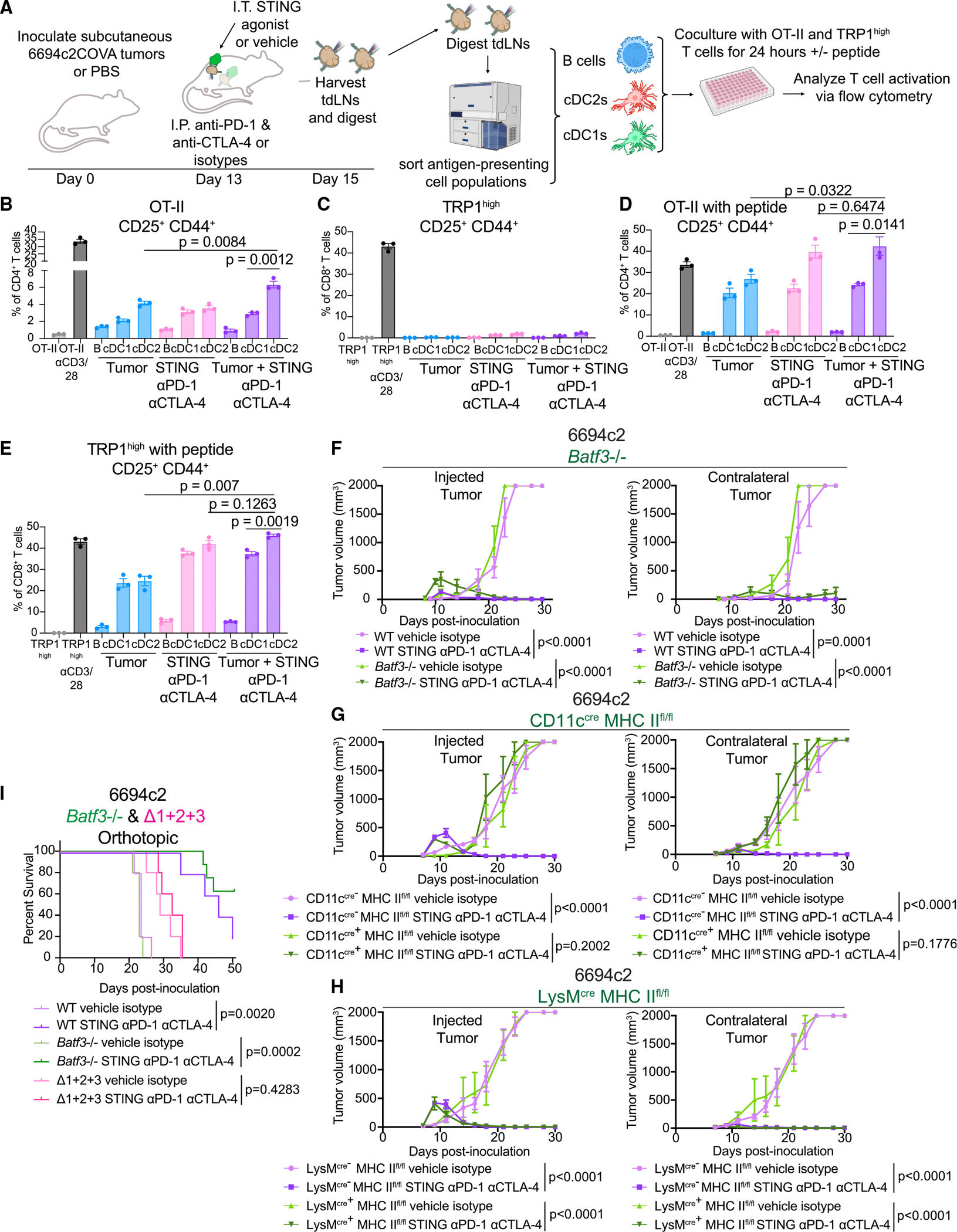
Dendritic cell priming of CD4^+^ T cells is required for pancreatic tumor rejection (A) Diagram of experiments in (B–E). Mice were inoculated with subcutaneous 6694c2COVA tumors. Seven days post-inoculation, intratumoral STING agonist or vehicle, and IP anti-PD-1 and anti-CTLA-4 or isotypes were injected. Another group of mice bearing sham tumors received subcutaneous STING agonist and IP anti-PD-1 anti-CTLA-4. *N* = 5. 48 h later, tdLNs were harvested and antigen-presenting cells sorted (Data S1). Antigen-presenting cells were co-cultured for 24 h with OT-II or TRP1^high^ T cells, either without (B and C) or with (D and E) additional cognate peptide. (B and C) Percentages of (B) OT-II T cells or (C) TRP1^high^ T cells activated *ex vivo* by tdLN antigen-presenting cells. (D and E) Percentages of (D) OT-II T cells or (E) TRP1^high^ T cells activated *ex vivo* by antigen-presenting cells when suboptimal (D) ovalbumin or (E) M9 peptide is added to coculture. (F–H) *In vivo* tumor growth curves from 6694c2-bearing (F) WT and *Batf3*−/− (G) CD11c^cre−^ MHC-II^fl/fl^ and CD11c^cre+^ MHC-II^fl/fl^, (H) LysM^cre−^ MHC-II^fl/fl^ and LysMc^cre+^ MHC-II^fl/fl^ mice. Experiment conducted as in Figure 1A. *N* = 5 except *N* = 4 in both *Batf3*−/− groups. (I) Overall survival of orthotopic 6694c2-bearing WT, *Batf3*−/−, and Δ1+2+3 mice. Experiment conducted as in Figures 4A and 4C–4E. *N* = 5 except *Batf3*−/− STING anti-PD-1 anti-CTLA-4 (*N* = 8). Bar plot significance (B–E) determined via *t* tests (error bars report SD). Tumor growth curve statistical significance (F–H) determined by calculating AUC in GraphPad Prism and comparing areas with *t* tests (error bars report SEM). Kaplan-Meier statistical significance determined via Log rank (Mantel-Cox) test (I). Illustrations from NIAID NIH BioArt Source: bioart.niaid.nih.gov/bioart/591; bioart.niaid.nih.gov/bioart/304; https://bioart.niaid.nih.gov/bioart/160; https://bioart.niaid.nih.gov/bioart/50; https://bioart.niaid.nih.gov/bioart/116; and https://bioart.niaid.nih.gov/bioart/7. See also Figure S9 and Table S1.

To determine whether cDC2s from treated mice could activate CD4^+^ T cells, mice were inoculated with ovalbumin-expressing 6694c2 (6694c2COVA) tumors or sham tumors and treated with triple combination therapy two weeks later. Mice were sorted into three treatment groups: mice bearing only 6694c2COVA tumors, mice bearing sham tumors treated with triple combination therapy, and mice bearing 6694c2COVA tumors treated with triple combination therapy. Tumor-draining lymph nodes were harvested 48 h later. Putative tumor-draining lymph node antigen-presenting cell populations (B cells, cDC1s, and cDC2s) were sorted and *ex vivo* cocultured with ovalbumin-specific OT-II T cells (CD4^+^ T cells) (Figure 5A and Data S1). TRP1^high^ T cells, which recognize the tyrosinase-related protein 1 (Tyrp1) melanoma antigen, were used as a negative control.^69,70^ We included a T cell that does not recognize ovalbumin to determine whether responses seen were antigen-specific, or whether they constituted nonspecific activation due to antigen-presenting cell costimulatory ligand expression and cytokine production. Out of all the groups, cDC2s isolated from mice bearing 6694c2COVA tumors and treated with triple combination therapy were best able to activate OT-II T cells *ex vivo* (Figure 5B). As expected, none of the antigen-presenting cells activated TRP1^high^ T cells *ex vivo*, indicating that T cell activation required recognition of cognate tumor antigen (Figure 5C). To test whether each of the antigen-presenting cell populations expressed functionally adequate MHC and costimulatory ligands, we included conditions where low amounts of exogenous ovalbumin or Tyrp1 peptides were added. When minimal exogenous antigen was provided, cDC1s and cDC2s from all groups were able to activate OT-II and TRP1^high^ T cells, though optimal activation came from cDC2s sourced from mice treated with combination therapy (Figures 5D and 5E).^71^ Collectively, it appeared that the main advantage of cDC2s over cDC1s was superior tumor antigen uptake.

We then wanted to ascertain whether dendritic cells were required for therapeutic efficacy, and if so, which populations were required. *Batf3*−/− mice, which lack cDC1s, were inoculated with tumors and treated with triple combination or control therapy. *Batf3*−/− mice rejected their tumors; cDC1s were not required for tumor control, likely because cross-presentation to CD8^+^ T cells was not required (Figures 5F and S9A). To determine whether dendritic cell priming of CD4^+^ T cells was required for therapeutic efficacy, we crossed CD11c^cre^ mice to MHC-II^fl/fl^ mice to conditionally delete MHC-II on dendritic cells. Tumor control was abrogated in these mice, showing that dendritic cell-expressed MHC-II was required (Figure 5G). In contrast, triple combination therapy cured LysM^cre^ MHC-II^fl/fl^ mice, which conditionally lack MHC-II on macrophages, indicating that macrophage expression of MHC-II was not required (Figure 5H). Finally, we examined the survival of *Batf3*−/− (cDC1-deficient) and Δ1+2+3 (cDC2-deficient) mice implanted with orthotopic 6694c2 tumors.^68^ *Batf3*−/− mice controlled their tumors, indicating a lack of requirement for cDC1s (Figure 5I). Tumor control was abrogated in Δ1+2+3 mice; cDC2s were required for pancreatic tumor control (Figure 5I). Curiously, Δ1+2+3 mice did control subcutaneous tumors, suggesting a more rigorous cDC2 requirement in the pancreas. We hypothesize that a Ly6C^+^ cell may functionally compensate for cDC2s in the subcutaneous setting (Figures S9A–S9C and Data S1).

### Human pancreatic cancer contains CD4^+^ T cells and cDC2s, even after multiagent chemotherapy treatment

**Figure 6.**
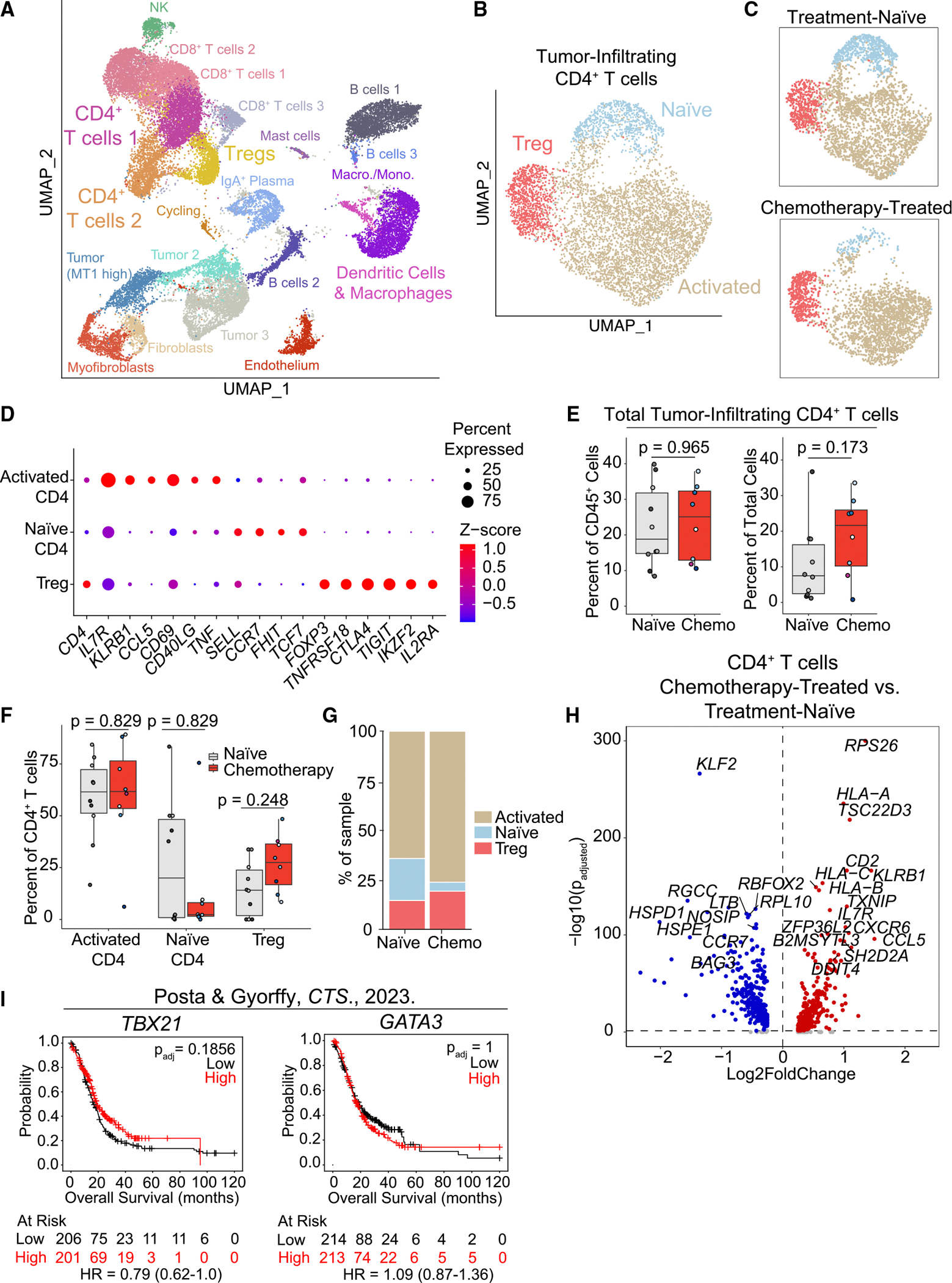
CD4^+^ T cells are present in human PDAC, and intratumoral CD4^+^ T cell frequencies do not change significantly with chemotherapy treatment (A) UMAP of all cells recovered from PDAC tumors. Dendritic cells/macrophage/monocyte cluster analyzed in Figures 7G–7J. (B) Subclustered CD4^+^ T cells. (C) CD4^+^ T cells split by chemotherapy treatment status. (D) Differentially expressed genes between CD4^+^ T cell clusters. (E) Frequency of tumor-infiltrating CD4^+^ T cells out of immune cells (left) and total cells (right). (F) Relative frequencies of the CD4^+^ T cell subsets in chemotherapy-naive and chemotherapy-treated patients. Box-and-whisker plot significance determined by Wilcoxon rank-sum test, followed by Benjamini-Hochberg correction. Magenta dot signifies FOLFOX-treated patient; all other chemotherapy-treated patients received FOLFIRINOX. (G) Relative frequencies of CD4^+^ T cell subsets. (H) Differentially expressed genes between CD4^+^ T cells from chemotherapy-treated and naive patients. (I) Kaplan Meier comparing high versus low *TBX21* (left) or *GATA3* (right) expression in patients with PDAC. Data from Posta & Gyorffy.^73^ See also Figure S10, Tables 1, and S2.

To understand how these findings may translate to patients with PDAC, we conducted scRNA-seq of surgically resected PDAC from treatment-naive and multiagent FOLFIRINOX- (5-FU, irinotecan, and oxaliplatin) or FOLFOX- (5-FU and oxaliplatin) treated patients (Tables 1 and S2).^72^ scRNA-seq of resected tumor samples revealed excellent recovery of immune cells, including CD4^+^ T cells, cDC1s, and cDC2s (Figure 6A; Figure S10). Sub-clustering of CD4^+^ T cells revealed naive, activated, and regulatory subsets (Figures 6B–6D). Total frequencies of CD4^+^ T cells did not change in chemotherapy-treated patients, either as a percent of immune cells or out of total cells recovered (Figure 6E). CD4^+^ T cell subset distributions also did not significantly change with chemotherapy treatment (Figures 6F and 6G). Differential gene expression of total CD4^+^ T cells in chemotherapy-treated versus naive tumors showed increased expression of chemokines/chemokine receptors important for dendritic cell and T cell trafficking (*CCL5* and *CXCR6*) but overall transcriptional similarity (Figure 6H). To determine whether differentiated CD4^+^ T cell subsets were associated with a survival benefit in patients with PDAC, we used an online tool from a published dataset.^73^ We found that neither of the canonical Th1 or Th2 transcription factors (*TBX21* and *GATA3)* were associated with a survival benefit (Figure 6I). We hypothesize that, although the endogenously primed CD4^+^ T cell response does not adequately control tumors, enhancing CD4^+^ T cell priming could still be beneficial.

**Figure 7.**
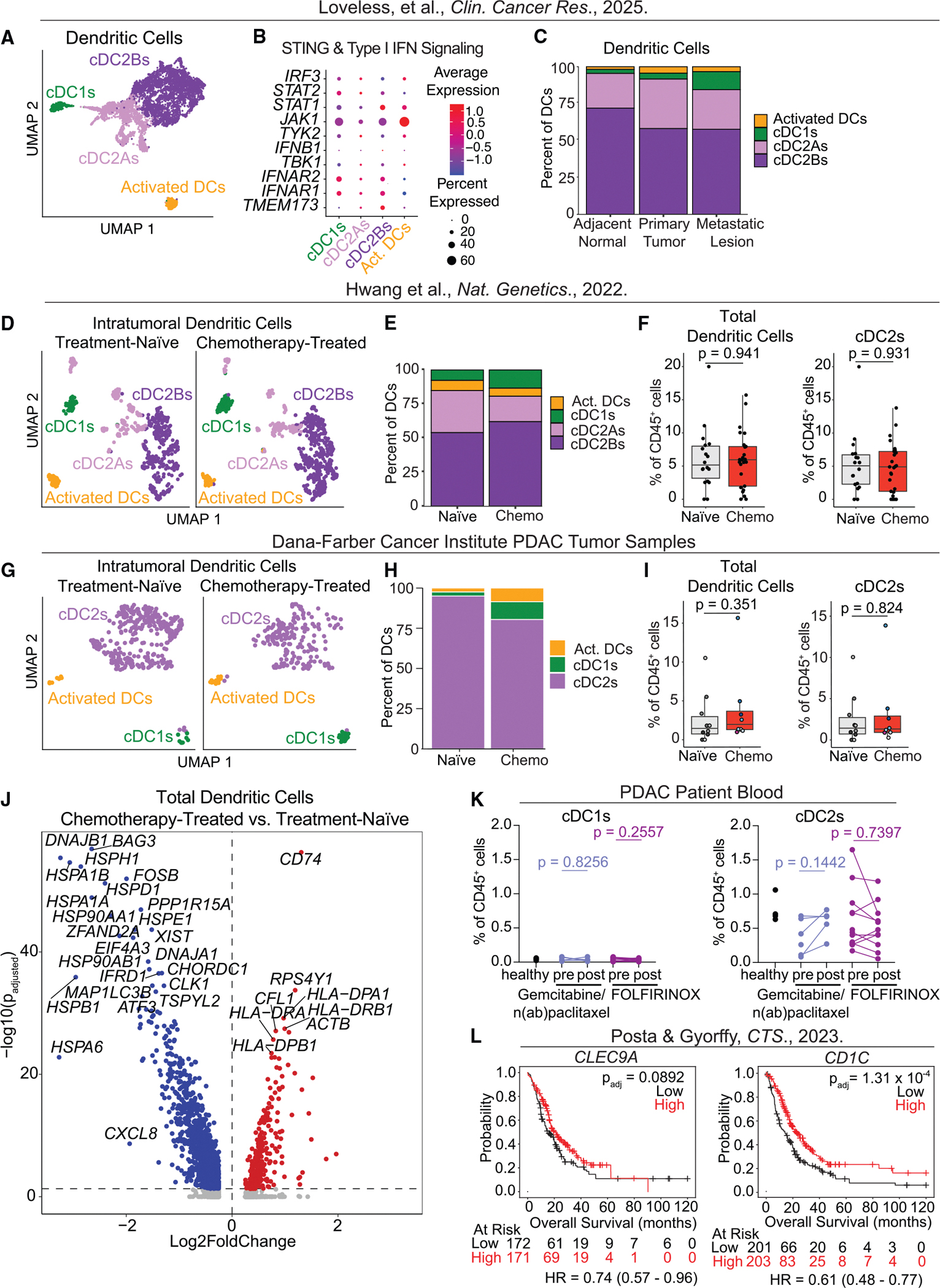
cDC2s are abundant in tumors and blood of patients with PDAC, and frequencies largely do not change with chemotherapy treatment (A–C) Human PDAC infiltrates reanalyzed from Loveless and colleagues.^74^ Patient samples were taken before chemotherapy treatment (Figure S11). (A) UMAP of dendritic cell subsets. (B) Type I IFN signaling and STING (*TMEM173*) mRNA expression in dendritic cell subsets. (C) Relative proportions of dendritic cell subsets in primary tumors, metastatic tumors, and adjacent normal tissue. (D–F) Human PDAC infiltrates reanalyzed from Hwang, Jagadeesh, Guo, & Hoffman and colleagues (Figure S12).^75^ (D) UMAP of intratumoral dendritic cell subsets split by chemotherapy treatment status. (E) Intratumoral dendritic cell subset relative frequencies in treatment-naive and chemotherapy-treated patients. (F) Total intratumoral dendritic cell (left) and cDC2 (right) frequencies in treatment-naı¨ve and chemotherapy-treated patients. (G–K) Dana-Farber Cancer Institute PDAC cohort (Figure 6; Figure S10). (G) UMAP of intratumoral dendritic cell subsets split by chemotherapy treatment status. (H) Dendritic cell subset relative frequencies in chemotherapy-naive and chemotherapy-treated patients. (I) Total dendritic cell (left) and cDC2 (right) frequencies. Magenta dot signifies FOLFOX-treated patient; all other chemotherapy-treated patients received FOLFIRINOX. (J) Differentially expressed genes between dendritic cells from chemotherapy-treated and chemotherapy-naı¨ve patients. (K) Patient-matched fractions of cDC1s and cDC2s (out of total CD45^+^ cells) in blood pre- and post-one cycle of FOLFIRINOX or gemcitabine(nab)paclitaxel. Analyzed by flow cytometry (Data S1). (L) Kaplan Meiers comparing high versus low *CLEC9A* (left) or *CD1C* (right) expression in patients with PDAC. Data from Posta & Gyorffy.^73^ Box-and-whisker plot significance determined by Wilcoxon rank-sum test. See also Figures S10–S12, Tables 1, S2, and Data S1.

Patients with PDAC have relatively few circulating cDC1s and cDC2s, and elevated blood cDC2 levels are correlated with improved survival.^45^ However, how cDC2 frequencies change in tumor and blood upon treatment with standard-of-care chemotherapies has not been reported. We investigated intratumoral dendritic cell frequencies in published datasets as well as in our own dataset. We reanalyzed data from Loveless et al. and identified dendritic cells by high *FLT3* and *FCER1A* expression (Figures 7A–7C; Figure S11^74^). Sub-clustering revealed four dendritic cell clusters: cDC1s, activated DCs, cDC2As, and cDC2Bs (Figure S11C; Figure 7A). Because interferon signaling can activate dendritic cells, we examined members of interferon signaling pathways, as well as *TMEM173* (STING), expression; all were expressed at comparable levels across subsets (Figure 7B). Dendritic cell subset relative frequencies did not change between normal tissue, primary tumor, and metastatic tumor sites, except for a slight increase in cDC1s at metastatic sites (Figure 7C). cDC2s comprised the vast majority of dendritic cells in all three sites (Figure 7C).

To evaluate dendritic cell population changes after chemotherapy treatment, we analyzed nuclear sequencing data from Hwang, Jagadeesh, Guo, & Hoffman et al. (Figure S12).^75^ Immune cell frequencies largely did not change after chemotherapy treatment (Figures S12A–S12C). Sub-clustering of myeloid cells revealed dendritic cells (Figures S12D–S12F). DCs were further sub-clustered to reveal populations of cDC1s, activated DCs, cDC2As, and cDC2Bs (Figures 7D and S12G). Neither total dendritic cell nor cDC2 frequencies changed after chemotherapy treatment (Figures 7E and 7F). Differentially expressed gene analysis indicated few changes between dendritic cells before and after chemotherapy treatment, although chemotherapy-treated dendritic cells had increased expression of *AREG* (Figure S12H).

In our own scRNA-seq of 18 human PDAC tumors, we subclustered the macrophage and macrophage/DC clusters (purple and magenta, Figure S10A) and removed non-dendritic cell populations, including neutrophils, monocytes, and macrophages (Figures S10B–S10F). After integrating dendritic cell, cDC1, and activated DC clusters, sub-clustering revealed cDC1s, activated DCs, cDC2As, and cDC2Bs (Figure 7G). Total dendritic cell frequencies, cDC2 frequencies, and relative frequencies of dendritic cell populations all did not change with chemotherapy treatment (Figures 7H and 7I). Differentially expressed genes between dendritic cells from chemotherapy-treated tumors compared to those from treatment-naive tumors revealed an increase in MHC-II presentation genes (*CD74*, *HLA-DPA1*, and *HLA-DRB1*) (Figure 7J). Together, these data indicate that dendritic cells, including cDC2s, remain an abundant therapeutic target after chemotherapy treatment.

To examine systemic effects of chemotherapy treatment on dendritic cell frequencies, peripheral blood mononuclear cells (PBMCs) were analyzed from patients with PDAC both before and after one cycle of FOLFIRINOX or gemcitabine/n(*ab*)paclitaxel therapy. Like healthy controls, patients with PDAC had roughly 10 times as many cDC2s as they did cDC1s in peripheral blood, and frequencies remained consistent after treatment with one cycle of either gemcitabine/n(*ab*)paclitaxel (28 days) or FOLFIRINOX (14 days) (Figures 7K and Data S1). Using a published dataset, we then asked whether intratumoral cDC1s or cDC2s might be associated with a survival benefit in patients with PDAC.^73^ While *CLEC9A* (expressed by cDC1s) expression was not associated with a survival benefit, *CD1C* (expressed by cDC2s) expression was associated with improved survival (Figure 7L). Taken together, these data indicate that cDC2s are more abundant than cDC1s in patients with PDAC, their numbers are not reduced by multiagent chemotherapy, and they present an attractive target for future therapies.

## DISCUSSION

Pancreatic cancer is notoriously unresponsive to immune checkpoint blockade and other therapies. Here, we showed that the addition of an immune adjuvant to immune checkpoint blockade induced durable remissions and protective immunologic memory in mice. More importantly, we showed that the anti-tumor immune response can be skewed toward an atypical, effective pathway requiring cDC2s, CD4^+^ T cells, and IFNγ. Migratory cDC2s primed IFNγ-producing CD4^+^ T cells in tumor-draining lymph nodes of treated mice. In multiple PDAC patient cohorts, CD4^+^ T cells and cDC2s persisted after multiagent chemotherapy treatment.

cDC2s are roughly 10 times more abundant than cDC1s in patients with PDAC. In cancer, cDC1s are considered to be the more effective antigen-presenting cell, yet, by sheer number, cDC2s provide more opportunities to prime anti-tumor T cells. Activating cDC2s in poorly immunogenic cancers could potentially generate new, productive anti-tumor T cell responses against previously elusive antigens. Because current cancer immunotherapies rely upon CD8^+^ T cells, they also rely upon cDC1 cross-presentation.^76^ We showed that immune checkpoint blockade-resistant tumors can be cleared by leveraging cDC2 priming of CD4^+^ T cells, thus circumventing requirements for cDC1s and CD8^+^ T cells.

In other cancer types, cDC1s uptake the most tumor antigen.^41,66^ It is unclear why cDC1s are particularly ineffectual in PDAC, though we hypothesize that it is at least partially due to cDC1 scarcity; we find far fewer cDC1s in our models than in other tumor models, consistent with other reports.^41,66^ In melanoma models, anti-CTLA-4 depletes intratumoral Tregs, releasing cDC2s to prime CD4^+^ T cells. Here, anti-CTLA-4, alone or combined with anti-PD-1, did not alter tumor growth kinetics, and no changes were found in Treg frequencies.^20,44,57^ Others found that increased cDC2 hematopoiesis is correlated with improved survival; our study shows additional beneficial functions of cDC2s in PDAC. Although multiagent chemotherapy and its associated supportive care often weakens CD8^+^ T cell responses, the preservation of MHC-II-presenting cDC2s and CD4^+^ T cells suggests that immunotherapies targeting the cDC2-CD4^+^ T cell axis could provide benefit even after multiagent chemotherapy.^77,78^

Primary and acquired resistance to immune checkpoint blockade is chiefly from downregulating tumor cell MHC-I expression, and here we describe a tumor rejection pathway that is agnostic to tumor cell MHC-I expression, MHC-II expression, and interferon response.^14^ Blockade of immune suppressive pathways in PDAC has been ineffective^58,79^; immune activation may be more productive in PDAC and other poorly immunogenic tumors. Adding an innate immune agonist to immune checkpoint blockade potently activates dendritic cells, produces a strong interferon response, and circumvents main resistance mechanisms, even in an otherwise unresponsive tumor model. STING agonist clinical trials have not yet seen success, in part due to variable drug design and difficulties identifying effective, safe delivery routes and therapeutic windows. STING agonists are only one tool that activates strong cDC2 and Th1 responses; we predict that other adjuvants and therapeutics that activate cDC2s to prime CD4^+^ T cells could be effective in patients with PDAC.

Although CD8^+^ T cells are traditionally considered to be responsible for tumor cytotoxicity, CD4^+^ T cells can kill tumor cells via direct and indirect mechanisms, including via activation of tumoricidal granulocytes and macrophages.^18^ Activated CD4^+^ T cells express CD40L, and the success of agonistic anti-CD40 in PDAC models and early-stage clinical trials suggests that activation of CD40 may provide another alternative pathway of tumor rejection.^80–82^ Multiple groups reporting effective anti-tumor immunity in PDAC models linked tumor regression to CD4^+^ T cells or IFNγ.^28,83^ Therapeutic induction of robust Th1 responses in PDAC is promising.

Why endogenously generated CD8^+^ T cell responses have thus far been insufficient for durable clinical benefit is unclear.^84,85^ One possibility is that antigens are inadequate for tumor rejection; provision of the strong antigen ovalbumin to poorly immunogenic PDAC models cleared tumors.^86^ In humans, MMRd PDAC responds well to immune checkpoint blockade, and tumor-specific memory CD8^+^ T cells persist in rare long-term survivors; antigen quality strongly determines anti-tumor immunity.^8,85^ How we can prime CD8^+^ T cells that recognize potent tumor antigens is unclear, nor is it clear that such antigens even exist in most patients with PDAC. Tumor-specific CD4^+^ T cells present an untapped opportunity, and mRNA and lipophilic peptide clinical trials suggest that CD4^+^ T cell antigens exist.^87,88^ Targeting cDC2s and leveraging MHC-II-presented peptides could reveal tumor antigens that yield productive anti-tumor CD4^+^ T cell responses in PDAC and other poorly immunogenic tumors.

## RESOURCE AVAILABILITY

### Lead contact

Further information and requests for resources should be directed to and will be fulfilled by the lead contact, Stephanie K. Dougan (stephanie_dougan@dfci.harvard.edu).

### Materials availability

Mouse and cell lines generated in this study are available upon request. There are restrictions to STING agonist availability because of lack of current supply.

### Data and code availability

The human scRNA-seq data generated in this study are publicly available in NIH dbGAP at phs004508 and phs004257. Mouse single-cell and bulk RNA-seq data are publicly available in GEO at GSE325195. Deposited data are available as of publication date. Other data generated in this study are available upon request. The paper does not report original code. Any additional information required to reanalyze the data reported in this paper is available from the lead contact upon request.

## STAR★METHODS

### EXPERIMENTAL MODEL AND STUDY PARTICIPANT DETAILS

#### Ethics statement

This study involves human participants and was conducted in accordance with the Declaration of Helsinki and the US Common Rule. Approval for #03–189 and #14–408 was granted by the DFCI IRB (IORG #0000035, FWA #00001121). Written informed consent was obtained from participants enrolled in #03–189 and #14–408 prior to sample collection. All animal protocols were approved by Dana-Farber Cancer Institute’s Institutional Animal Care and Use Committee (IACUC) (protocol #14–019 and 14–037). They are all in compliance with the NIH/NCI ethical guidelines for tumor-bearing animals.

#### Human samples

Whole blood and surgically resected tumor samples were collected from patients with PDAC receiving gemcitabine/n(*ab*)-paclitaxel, FOLFIRINOX, or FOLFOX at DFCI under protocol #03–189 and #14–408. Healthy donor peripheral blood mononuclear cells were obtained from deidentified leukapheresis cones from the Kraft Blood Donor Center. Information regarding patient number and demographics can be found in Tables 1 and S2. Analyses of how sex, gender, ancestry, race, ethnicity, and socioeconomic status affected our results were not performed, and thus limit the generalizability of the results. Additional demographic (age, sex, and race) and diagnostic information found in Tables 1 and S2. *N* = 18 patients.

#### Animal strains

All animal protocols were approved by the Institutional Animal Care and Use Committee (IACUC) of the Dana-Farber Cancer Institute (DFCI) (protocol #14–019, 14–037, 10–055) and are in compliance with the NIH/NCI ethical guidelines for tumor-bearing animals. When possible, 6–12-week-old female mice were used. Experiments were age- and sex-matched, and littermate controls were used whenever possible. Mice had not undergone previous procedures. Mice were group-housed and randomized both before tumor inoculation and after inoculation but before first therapeutic dosing. Mice were randomized by combining into one cage then separating blindly. All mice were purchased from the Jackson Laboratory: C57BL/6 (Stock No. 000664), *μMT*−/− (002249), *Batf3*−/− (013755), *TCRα*−/− (002116), *β2M*−/− (002087), *I-Ab*−/− (005589), *Rag2*−/− (008449), *Perforin*−/− (002407), *Ifnar1*−/− (028288), *Ccr2*−/− (004999), iNOS−/− (002609), Δ1+2+3 (037704), OT-II (004194), OT-I (003831), CD11c^cre^ (008068), MHC-II^fl/fl^ (013181), LysM^cre^ (004781), and Tbet−/− (Jackson Labs # 004648). TRP1^high^ mice were bred in-house.^69^ MR1−/− mice were generated via CRISPR Cas9 insertion into a fertilized zygote. They were then crossed to TCRδ−/− mice from Jackson Laboratory (002120) to generate MR1−/− TCRδ−/−.^95^ CD11c^cre^ mice and LysM^cre^ mice were bred to MHC-II^fl/fl^ mice in-house. *N* = 5 mice (10 tumors) per group unless otherwise stated. Studies were sex-matched, and analyses of the influence of sex on the results were not performed, which limits the study’s generalizability. However, most key experiments were conducted in both male and female mice.

#### Cell lines

6694c2 and 6419c5 cells were derived from LSL-KrasG12D;p53^+^/floxed, Pdx-cre, YFP-floxed female mice and were gifted by Ben Stanger (University of Pennsylvania).^2^ Cells were cultured at 37°C in RPMI media (Life Technologies) supplemented with 10% (v/v) inactivated fetal bovine serum, 2 mmol/L L-glutamine (Gibco), 1% (v/v) penicillin/streptomycin (Gibco), 1% (v/v) MEM non-essential amino acids (Gibco), 1 mmol/L sodium pyruvate (Gibco), and 0.1 mmol/L β-mercaptoethanol (Sigma). Cells were passaged upon reaching 70% confluence.

### METHOD DETAILS

#### Human blood processing

Whole blood was processed within 24 h of collection for PBMC isolation by gradient centrifugation using Ficoll-paqueplus (Cytiva, #45001749). Cells were frozen in 90% FBS (Gibco)/10% DMSO (Dimethyl sulfoxide) (Sigma-Aldrich) at a maximum concentration of 5 × 10^6^ cells/mL and stored in liquid nitrogen.

#### CRISPR-Cas9 cell line generation

Various genetic knockout cell lines were engineered via CRISPR-Cas9. For the *Caspase 8*−/−, *Ciita*−/−, and *Stat1*−/− lines, CRISPR modifications were made using the lentiCRISPRv2 plasmid (Addgene #52961) digested with *BsmBI* (NEB). Single guide RNAs were cloned into the vector with T4 DNA ligase (NEB), and lentivirus was made and transfected into cells as previously described.^28^ In brief, lentivirus was generated by HEK293T cell (from American Type Culture Collection) transfection with lentiCRISPRv2 (Addgene #52961), psPAX2 (Addgene #12260), and pVSV-G (Addgene #8454) plasmids. Lentiviral supernatants were collected 48 and 72 h post transfection, passed through a 0.45μM filter, and used to transduce tumor cells; puromycin was used to select cells. Western blot was performed to confirm knockout using the following primary antibodies: anti-caspase 8 antibody (Cell Signaling #4927) and anti-GAPDH (Cell Signaling #3683 S).

The 6694c2COVA cell line was created via lipofectamine transfecting the eGFP-ova-c1 plasmid (Addgene #163524) into 6694c2 cells, followed by geneticin selection and sorting. The 6694c2 ZsGreen cell line was generated by transfecting the pUBC-ZsGreen plasmid into 6694c2 cells. Cells were selected with hygromycin (Invivogen) and ZsGreen^+^ cells were sorted. 6694c2 IFNγR1−/− cells were generated with the pSpCas9(BB)-2 A-Puro (PX459) V2.0 (Addgene #62988) plasmid. Single-guide RNAs were introduced into the plasmid with BbsI (NEB) and T4 ligase (NEB). Plasmids were transfected into cells via Lipofectamine (ThermoFisher) transfection and selected with puromycin (2 μg/mL) (Fisher Scientific). 6694c2 *Stat1*−/−, 6694c2 *IFNγR1*−/−, and 6694c2 *Ciita*−/− were confirmed via treatment with IFNγ/IFNα for 24 h, followed by flow cytometry analysis of MHC-I/MHC-II and PD-L1.

#### Immunoblotting

Tumor cells were grown in culture and then trypsinized (Life Technologies #25200114). Then, cells were lysed in 0.5% NP-40, 50mM HEPES, 50mM NaCl, protease and phosphatase inhibitor (Sigma Aldrich #11836170001). A bicinchoninic acid (BCA) assay was performed to determine lysate concentrations, and samples were boiled at 95°C. Equal lysate amounts were loaded to a 4 to 20% SDS-polyacrylamide gel. After gel electrophoresis, transfer to a PVDF membrane was conducted via the Bio-Rad *Trans*-blot Turbo Transfer System. The membrane was then incubated with primary anti-caspase 8 antibody (Cell Signaling #4927) and anti-GAPDH (Cell Signaling #3683 S) in 3% BSA in TBST (tris, NaCl, and Tween 20). Membranes were incubated with HRP-conjugated secondary antibody and then with chemiluminescence substrate prior to imaging (Data S1).

#### Subcutaneous tumor inoculations

250,000 6694c2 or 6419c5 cells were subcutaneously injected into animals’ right and left flanks in sterile HBSS (Gibco). Mice received bilateral tumors. Tumor size was measured 2–3 times weekly, and tumor volume calculated by multiplying the three dimensions of the tumor. Mice were euthanized by CO_2_ asphyxiation when tumors reached >2000 mm^3^ or had gross ulceration. Tumor volumes are plotted as mean ± SEM. Timepoints start at day 0 = day of inoculation unless otherwise noted.

#### Orthotopic tumor inoculations

250,000 6694c2 cells were subcutaneously injected into animals’ right flank in sterile HBSS (Gibco). On the same day, 50,000 6694c2 cells in Matrigel (Corning) suspension were injected into the pancreas via a survival surgery, as previously described.^56,57^ Tumor size was measured 2–3 times weekly, and tumor volume calculated by multiplying the three dimensions of the tumor. Mice were euthanized by CO_2_ asphyxiation when orthotopic tumors reached >2000 mm^3^. Midpoint harvest measurements were conducted 14 days post-inoculation. Timepoints start at day 0 = day of inoculation unless otherwise noted.

#### *In vivo* treatments

When subcutaneous tumors became palpable (day 6–7 post-inoculation), one tumor was injected with 100 μg of STING agonist (BMS-986301) in 30 μL endotoxin-free sterile PBS (Millipore Sigma).^96^ On the same day as STING agonist injection, mice were treated intraperitoneally with 10 mg/kg anti-PD-1 (RMP1–14) and 10 mg/kg anti-CTLA-4 (9D9). While mice only received a single dose of STING agonist therapy, checkpoint blockade was dosed weekly for a total of four doses. Control mice were given intratumoral PBS and intraperitoneal doses of each isotype control. Mice were treated with a total of four doses of checkpoint blockade for each experiment (three doses following the first dose coincident with the STING agonist). Depleting antibodies were all purchased from BioXCell and delivered at the same dose (150 μg/mouse, delivered intraperitoneally in 150 μL endotoxin-free sterile PBS every 2 days). Depleting/blocking antibodies used in these studies included: anti-CD4 (#BE0003–1), anti-CD8 (#BE0061), anti-NK1.1 (#BE0036), anti-IFNγ (#BE0054), anti-TNFα (BE#0058), anti-Ly6G (#BE0075), anti-Ly6C (#BE0203), and anti-CSF1R (#BE0213). Depletion was generally started 3–7 days prior to tumor inoculation. FTY720 (Sigma Aldrich #SML0700) was delivered via oral gavage treatment, and each mouse received 75 μg FTY720 in 100 μL water every day, starting on day 6 post-inoculation and continuing daily thereafter. Mice were bled at endpoint to verify FTY720 function (Data S1). “Cure rates” (number of mice tumor-free at day 30–35) for mice that received triple combination therapy are reported in Table S1 and in supplemental figure legends (Table S1).

#### Metastasis model

180,000 6694c2-met cells were subcutaneously injected into animals’ dorsal surface in sterile HBSS (Gibco). Seven days later, 100 μg STING agonist or vehicle was injected into the subcutaneous tumor in 30 μL ET-free PBS. Four days after that (11 days post-inoculation), the tumor was surgically removed, as previously described.^58^ 6694c2-met cells were *in vivo* passaged to select for lung and lymph node metastatic tropism, so 35 days later, mice were euthanized by CO_2_ asphyxiation and lungs and lymph nodes harvested to examine for metastases. Metastases were counted.

#### Draining lymph node harvest

Tumor-draining lymph nodes were harvested from mice two days post-STING agonist therapy. Briefly, lymph nodes were placed in 2 mL of digestion media in 6 well plates. Digestion media was composed of RPMI 1640 1× supplemented with 0.8 mg/mL dispase (Life Technologies #17105041), 0.2 mg/mL collagenase P (Sigma #11249002001), and 0.1 mg/mL DNAse I (Sigma #10104159001). Lymph nodes were pierced, incubated at 37°C for 30 min, and then manually digested via pipetting. Supernatants were transferred to 15 mL conical tubes with 2 mL ice-cold FACS buffer, and any remaining fragments were digested with an additional 2 mL of digestion media. Supernatants were washed with more ice-cold FACS buffer, and, following the digestion of remaining fragments, the supernatants were combined between digestions. Digestion supernatants were centrifuged at 300 g for 4 min and again washed with ice-cold FACS buffer. Cells were then ready for use in flow cytometry or coculture experiments (Data S1). Protocol was adapted from Fletcher et al.^97^

#### Bulk RNA sequencing

6694c2 and B16F10 tumor cells were treated with IFNγ (100 ng/mL) (Peprotech #315–05), IFNβ (1000 U/mL) (BioLegend #581302), and STING agonist (225 μg/mL) in culture for 24 h. Cells were harvested and RNA isolated with a QIAGEN RNAeasy Plus Mini Kit (#74134). RNA was sent to Genewiz for paired-end RNA sequencing. In brief, FASTQ files were put through quality control (fastqc 0.11.9 & multiQC v1.5 for collating fastqc results), adaptor trimming (cutadapt v.1.14), genomic alignment (STAR v2.7.9a), and read count analyses (Rsubread) via the Harvard O2 cluster. Filtering for genes with less than 10 reads combined across samples was conducted, and analysis of differential gene expression was conducted with DESeq2.^94^ Plots were generated with ggplot2 in R.

#### Lymph node dendritic cell flow cytometry

Lymph nodes were harvested from vehicle- and STING anti-PD-1 anti-CTLA-4-treated mice and digested. The lymph node single-cell suspension was washed in ice-cold FACS buffer and then stained in FACS buffer (PBS with 2% IFS and 2mM EDTA). Stains were composed of Sirpα PE Dazzle 594 (BioLegend #144016), CD11b Pacific Blue (BioLegend #101224), XCR1 Brilliant Violet 421 (BioLegend #148216), CD103 Brilliant Violet 605 (BioLegend #121433), CD11c APC (BioLegend #117310), IA/IE Brilliant Violet 510 (BioLegend #107636), CD80 PE (BioLegend #104708), CD8α Brilliant Violet 785 (BioLegend #100750), Ly6C PE-Cy7 (BioLegend #128018), F4/80 Brilliant Violet 605 (BioLegend #123133) or F4/80 PE Dazzle 594 (BioLegend #123146). Tumor cells expressed ZsGreen (6694c2 ZsGreen).^98^

#### Antigen-presenting cell-T cell coculture

C57BL6/J mice were inoculated subcutaneously with four flank tumors of 250,000 6694c2COVA cells, and a separate group of mice received sham inoculations. On day 11 post-inoculation, mice were randomized into two groups: one group with 6694c2COVA tumors received intratumoral STING agonist and IP anti-PD-1 and anti-CTLA-4, and the other group received vehicle and isotype. The sham-inoculated group received STING agonist anti-PD-1 and anti-CTLA-4. Two days post-treatments, tumor-draining lymph nodes (axillary and inguinal) were harvested and digested. Lymph node digests were processed with a CD90.2 positive selection kit (STEMCELL #18951) to remove T cells, but rather than keeping the T cells, the non-T cell supernatants were reserved. T cells were removed to enrich for antigen-presenting cells in the lymph node digests and to speed up sorting time. The T cell-depleted lymph node digests were sorted via FACS into three antigen-presenting cell populations: B cells, cDC1s, and cDC2s (see Data S1 for gating scheme). The following antibodies were used: CD11b Pacific Blue (BioLegend #101224), CD11c APC (BioLegend #117310), IA/IE Brilliant Violet 510 (BioLegend #107636), CD19 PE (BioLegend #152408), Gr1 PE-Cy7 (BioLegend #108416). In the meantime, CD8^+^ T cells were isolated from TRP1^high^ mice, and CD4^+^ T cells were isolated from OT-II mice. T cells were resuspended in hIL2 (PeproTech #200–02) media and plated in a 96-well V-bottom plate at 40,000 T cells per well. Post-sorting, antigen-presenting cells were washed and plated at a 1:3 ratio with T cells, such that each antigen-presenting cell from each treatment group was cocultured with each other kind of T cell in triplicate. T cells were also cocultured with their cognate peptide (ISQAVHAAHAEINEAGR for OT-II, and TAPDNLGYM for TRP1^high 69^). After 48 h of coculture, cells were harvested and stained for flow cytometry analysis with the following panel: CD8α Brilliant Violet 785 (BioLegend #100750), CD4 Brilliant Violet 510 (BioLegend #100559) CD69 PE (BioLegend #104508), PD-1 APC (BioLegend #135210), CD44 Brilliant Violet 421 (BioLegend #103039), and CD25 Brilliant Violet 711 (BioLegend #102049).

#### Tumor cell growth kinetics

Tumor cells were plated at 5,000 cells per well in 96-well Greiner Bio-One (#655090) plates. Cells were plated in 200 μL of media supplemented with vehicle, IFNγ, IFNα, or STING agonist at noted concentrations. 12 h post-plating, tumor cell confluence was measured and analyzed on a Celigo Imaging Cytometer (Nexcelom Bioscience). Confluence was measured and analyzed every 24 h thereafter, until cells reached 100% confluence in control conditions.

#### Intracellular and serum cytokine analysis

Mice were inoculated with 6694c2 tumors, and on day 15, tumors and tumor-draining lymph nodes were harvested. A few hours prior to harvest, mice were injected intraperitoneally with 5 μg brefeldin A (Sigma #B7651). Tumors were macerated and digested in collagenase IV (Sigma #C5138) and soybean trypsin inhibitor (Life Technologies #17075029). Tumor chunks were removed via filtration with a 70 μM filter, and the resulting single cell suspension was washed with PBS 1×. Tumor-draining lymph nodes were digested as described above in “Draining Lymph Node Harvest,” and washed with 1 mL PBS. Cells were stained with 100 μL extracellular stain for 10 min at room temperature: CD45 Brilliant Violet 711 (BioLegend #103147), CD69 PE (BioLegend #104508), CD4 Brilliant Violet 510 (BioLegend #100553), CD25 PE-Cy7 (BioLegend #102016), and CD8 APC (BioLegend #100712). Cells were then washed with 900 μL PBS 1× and centrifuged at 500 g for 5 min. After discarding supernatants, cells were incubated in 200 μL Fixation Buffer (BioLegend #420801) for 20 min at room temperature. Cells were centrifuged at 700 g for 5 min. Supernatants were discarded and cells resuspended in 1 mL of permeabilization/wash buffer (BioLegend #421002). Cells were centrifuged at 700 g for 5 min. This step was repeated, and then cells were stained with the intracellular stain: IFNγ Brilliant Violet 421 (BioLegend #505830). Cells were stained overnight in 100 μL of intracellular stain, and then 200 μL permeabilization/wash buffer was added prior to running samples on the Sony SP6800 flow cytometer. For serum cytokine analysis, tumor-bearing mice were bled retro-orbitally, and serum was isolated from blood via centrifugation. Serum was then used to conduct an IFNα ELISA, following manufacturer’s instructions (Life Technologies #BMS6027).

#### ELISpot

C57BL6/J mice were treated with vehicle, anti-PD-1 and anti-CTLA-4, STING agonist, and STING agonist anti-PD-1 and anti-CTLA-4 therapy as described above. The ELISpot plate was pre-treated with filtered 70% ethanol before it was washed with sterile PBS. It was then incubated with IFNγ capture antibody (BD Biosciences #551881) overnight at 4°C. Anti-CD3ε antibody was added to positive control wells (BioLegend #100340). After washing with PBS 1×, the plate was blocked with 10% FBS in PBS overnight at 4°C. IFNγ-stimulated 6694c2 cells were plated in all wells, minus the positive control wells. One day after the second anti-PD-1 and anti-CTLA-4 doses, tumor-draining lymph nodes were harvested from treated, tumor-bearing mice with sterile technique. Lymph nodes were macerated, and cells were plated on top of the pre-plated tumor cells. Human IL-2 (PeproTech #200–02) was added to the media, and positive control wells received anti-CD28 as well (BioLegend #102116). The ELISpot assay was incubated at 37°C for 24 h before being washed with sterile water and PBST, and IFNγ detection antibody (BD Biosciences #551881) was added. Assay was then incubated in detection antibody for 2 h at room temperature. Following washes with PBST, streptavidin-HRP (BD Biosciences) was added and incubated for one hour at room temperature. After washing with PBS and PBS, AEC chromagen substrate (BD Biosciences) was added, and plate developed, dried, and analyzed.

#### Murine scRNA-seq tissue preparation

Mice bearing ZsGreen-expressing 6694c2 tumors were treated as described above. 48 h after treatment, tumors, spleens, and tumor-draining lymph nodes were harvested from mice. Spleens and lymph nodes were macerated. Tumors were macerated and digested in collagenase IV (Sigma #C5138) and soybean trypsin inhibitor (Life Technologies #17075029). Tumor chunks were removed via filtration with a 40 μM filter, and the resulting single cell suspension was washed with PBS. Part of the tumor, lymph node, and spleen sample was taken for flow cytometry analysis. The rest of the samples were hashed, sorted based on ZsGreen expression on a BD FACS Aria, and analyzed via single-cell sequencing analysis.

#### Murine scRNA-seq analysis

Sample data were analyzed in R with *Seurat*.^99^ The 10× Cellranger pipeline (v7.0.0) was used to align reads to the Mm10 *Mus musculus* genome (from 10× Genomics). CITE-seq-Count was used to count hashtag reads. Hashtag reads were used to demultiplex samples with the *Seurat* ‘HTODemux’ function using the default settings. Only cells classified as a “singlet”, but not as a “doublet” or “negative” by hashtag demultiplexing were kept. Following hash demultiplexing, cells were further filtered out if they contained more than 20% mitochondrial (*mt*-) reads. ZsGreen^−^ and ZsGreen^+^ tumor-draining lymph node samples were integrated together and clusters identified in a similar manner as human samples (see below). Three clusters of lower quality cells or doublets were removed after cluster identification. Genes from the Molecular Signatures Database (MSigDB) “Hallmark Interferon Gamma Response” and “Hallmark Interferon Alpha Response” (v7.2) were z-scaled and summed to visualize an IFN score.

The three CD4^+^ T cell clusters from the main tumor-draining lymph node dataset were subset, and new variable features were discovered. The dataset was scaled, and top 15 PCs used for UMAP embedding and clusters identified. Following cluster identification, two clusters consisting of dendritic cells and B cells were removed, and the process of generating the UMAP and clustering was repeated. The “DCs” cluster from the main tdLN dataset were subset, new variable features identified, a new UMAP generated from the top 15 PCs, and clusters identified as above. This new sub-clustered object had 2 clusters comprised of either T cells or B cells; these clusters were subsequently removed as well as any cell with *Cd79b* expression. This filtered, subclustered dataset was reprocessed with the above steps. Differentially expressed genes between treatment groups were identified with the ‘FindMarkers’ function.

#### Human peripheral blood dendritic cell analysis

Patients diagnosed with pancreatic ductal adenocarcinoma had blood drawn prior to starting their chemotherapy regimen and after they had started their chemotherapy. Blood peripheral blood mononuclear cells were stained: Zombie NIR (BioLegend #423105), Human CD45 PE-CF594 (BD Biosciences #562279), CD34 BV510 (BioLegend #343528), CD20 Pacific Blue (BioLegend #302328), CD3 Pacific Blue (BioLegend #300418), CD14 Pacific Blue (BioLegend #301828), CD16 Pacific Blue (BioLegend #302032), CD19 Pacific Blue (BioLegend #302232), HLA-DR BV711 (BioLegend #307644), CD45RA PE-Cy7 (BioLegend #304126), CD33 FITC (BioLegend #366620), CD123 BV605 (BD Biosciences #740412), CADM1 Alexa Fluor 647 (MBL International #CM004-A64), and CD1c PE (BioLegend #331506). Samples were analyzed on a Sony SP6800 flow cytometer (Data S1).

#### Human scRNA-seq tissue processing

Viably frozen patient pancreatic tumor tissue from surgical resections was thawed in a 37°C water bath and then manually dissociated with a sterile razor. Dissected tissue was transferred to pre-warmed 37°C digestion enzyme mix in RPMI (Miltenyi tumor dissociation kit #130–095-929). While in a 37°C water bath, the digestion mixture was agitated every 2 min and pipetted up and down every 10 min for a total of 30 min. Digested tissue was filtered through a 70 μM strainer and washed with RPMI containing 10% FBS, and the single-cell suspension was resuspended in PBS 1×. Cells were stained with Zombie-NIR and anti-CD45. The CD45^+^, Zombie^−^ cells were captured in 20% FBS in RPMI on a BD FACS Aria II at the Dana Farber Flow Sorting Core.

Cells were counted on a hemacytometer and resuspended to a concentration of 1000 cells/μL in PBS with 0.05% ultrapure BSA. Cells were then loaded onto a Chromium X with the Single Cell K Chip (PN-2000182), the Chromium Next GEM Single Cell 5ʹ GEM Kit v2 reagents, and beads (PN-1000244). Library preparation was performed with the Library Construction Kit (PN-1000190). Libraries were pooled and sequenced on an Illumina NovaSeq 6000 instrument with 2 × 150bp sequencing (Azenta Life Sciences).

#### Human Kaplan-Meier plots

Graphs were prepared using https://server2.kmplot.com/pancreas, selecting PDAC only, and comparing Q1 and Q4. Adjusted *p* value was calculated by performing Bonferroni correction for multiple hypotheses on the *p* values provided.

#### Dana-Farber Cancer Institute scRNA-seq analysis

The 10× Cellranger pipeline (v7.0.0) was used to align reads to the hg38 *Homo sapiens* genome (from 10× Genomics). Count matrices from Cellranger were loaded into *R* using the *Seurat* package (v4.3.0). Using the *DropletUtils* package (v1.18.1), empty droplets were excluded with an FDR less than 0.01 and genes expressed in fewer than 5 cells were removed.^93^ High quality cells with greater than 750 genes and less than 20% mitochondrial (*MT*-) reads were kept. Three lower-quality samples were filtered to just those cells with 1,000 genes and less than 10% mitochondrial reads. Following log-normalization of each individual Seurat object, 2,000 integration anchors were identified to combine patient samples together and minimize batch effects.^100^ The integrated dataset was scaled, and the 20 top principal components (PCs) were used for Uniform Manifold Approximation and Projection (UMAP) embedding. Clusters were identified with a Shared Nearest Neighbor (SNN) graph based on each cell’s 20-nearest neighbors and then applying modularity refinement with the Louvain algorithm. Markers for each cluster were identified using the ‘FindAllMarkers’ function.

From the integrated dataset, cells were subset from the clusters identified as “Mac/Mono” and “Mac/DC” with normalized expression of *CD3D* and *MS4A1* below 1. The myeloid subset object had a new UMAP embedding and clusters identified, from which contaminating clusters expressing *COL4A1, CD3E,* or *C1QC* were removed. A new myeloid/DC subcluster UMAP and clusters were identified after scaling while regressing out mitochondrial and ribosomal (*RP[SL]*) reads. A new UMAP embedding was calculated using the top 15 PCs and clusters identified as described above.

From this myeloid/DC subset, “Dendritic cells”, “cDC1s”, and “Activated DCs” clusters were further isolated and a contaminating *ZBTB46*/*FLT3*-low cluster was removed from the “Dendritic cells” cluster to generate the final UMAP embedding for DCs.

CD4^+^ T cells were subclustered from the total integrated dataset by isolating the clusters “T cells 1”, “T cells 4”, and “Tregs” as well as normalized expression of *CD8A* and CD8*B* at zero. New variable features (3000) were calculated and used for integration of a CD4^+^ T cell object that was re-scaled with regression of mitochondrial and ribosomal reads. The top 10 PCs were used to generate clusters and a new UMAP embedding.

Percentages of dendritic cells from CD45^+^ cells were calculated by finding the number of DCs per patient and dividing by CD45^+^ cells (cells from T cell, NK cell, B cell, Plasma cell, Macro/Mono, Macro/DC, and Mast cell clusters). Percentages were compared with a Wilcoxon rank-sum test.

Differentially expressed genes were calculated with the ‘FindMarkers’ function.

#### Published dataset human scRNA-seq analysis

Loveless et al. Clin. Cancer Res. 2025.

Files were loaded from https://zenodo.org/records/14199536. Data were analyzed as written above, and the *harmony* package (v1.2.3) was used for batch correction/integration in conjunction with the Seurat pipeline (v5.3.0).^92^ “MYELOID” and “CYCLING MYELOID” cells were subclustered from the main dataset. Cluster markers were identified using ‘FindAllMarkers’. Based on high *FLT3* expression, the dendritic cell cluster was defined. Subclustering dendritic cells revealed other myeloid contaminants, which were excluded. A new dendritic cell UMAP was calculated and clusters identified.

Hwang, Jagadeesh, Guo, & Hoffman et al., Nat. Genetics., 2022.

GSE202051 was loaded. Data were analyzed as written above, and the harmony package was used for batch correction/integration in conjunction with the Seurat pipeline (v5.3.0). Immune cells were identified using ‘FindAllMarkers’. The myeloid cell cluster was identified and subclustering performed. A new UMAP of myeloid cells was created. Based on high *FLT3* expression, a dendritic cell cluster was defined. Subclustering dendritic cells revealed other myeloid contaminants, which were excluded. A new dendritic cell UMAP was calculated and clusters identified.

### QUANTIFICATION AND STATISTICAL ANALYSIS

#### Data analysis

Pairwise, groups comparisons, and correlation analyses were performed using the Wilcoxon matched-pairs signed rank test, one-way ANOVA test, and Pearson coefficient calculation. GraphPad Prism version 10 was used for statistical analysis. RNA sequencing data were analyzed in R. Statistical details are found in figure legends and in appropriate methods sections. N = number of animals unless otherwise stated.

## Supplementary Material

Supplemental Figures

Supplemental data file

Supplemental information can be found online at https://doi.org/10.1016/j.ccell.2026.04.005.

## Figures and Tables

**Table 1.** Patient cohort demographics

Characteristic	*N* = 38

**Age, median (Range), years**	67 (29–80)

**Sex, n (%)**	

Female	19 (50%)
Male	19 (50%)

**Race, n (%)**	

White	36 (95%)
Black	1 (3%)
Other	1 (3%)

**Diabetes, n (%)**	

No	25 (66%)
Yes	13 (34%)

**Stage, n (%)**	

I-III	24 (63%)
IV	14 (37%)

**Liver metastases at diagnosis, n (%)**	

No	25 (66%)
Yes	13 (34%)

**Chemotherapy regimen, n (%)**	

FOLFIRINOX	21 (55%)
Gemcitabine, nab-paclitaxel	6 (16%)
FOLFOX	1 (3%)
None	10 (26%)

Patient demographics. See also Table S2.

**KEY RESOURCES TABLE:** 

REAGENT or RESOURCE	SOURCE	IDENTIFIER

Antibodies		

anti-caspase 8	Cell Signaling	Cat# 4927; RRID: AB_2068301
anti-GAPDH	Cell Signaling	Cat# 3683 S; RRID: AB_1642205
Anti-Rabbit IgG, HRP	Cell Signaling	Cat# 7074; RRID: AB_2099233
Anti-H2-D^b^/K^b^ AF647	BioLegend	Cat# 114612; RRID: AB_492931
Anti-PD-L1 PE	BioLegend	Cat# 155403; RRID: AB_2728222
anti-PD-1	Bristol Myers Squibb	Clone: RMP1-14
anti-CTLA-4	Bristol Myers Squibb	Clone: 9D9
anti-CD4	BioXCell	Cat# BE0003-1; RRID: AB_1107636
anti-CD8	BioXCell	Cat# BE0061; RRID: AB_1125541
anti-NK1.1	BioXCell	Cat #BE0036; RRID: AB_1107737
anti-Ly6G	BioXCell	Cat #BE0075-1; RRID: AB_1107721
Anti-Ly6C	BioXCell	Cat# BE0203; RRID: AB_2687696
Anti-CSF1R	BioXCell	Cat# BE0213; RRID: AB_2687699
Anti-IFNγ	BioXCell	Cat# BE0054; RRID: AB_1107692
Anti-TNFα	BioXCell	Cat# BE0058; RRID: AB_1107764
anti-Sirpα PE Dazzle 594	BioLegend	Cat# 144016; RRID: AB_2565280
anti-CD11b Pacific Blue	BioLegend	Cat# 101224; RRID: AB_755986
anti-XCR1 Brilliant Violet 421	BioLegend	Cat# 148216; RRID: AB_2565230
anti-CD103 Brilliant Violet 605	BioLegend	Cat# 121433; RRID: AB_2629724
anti-CD11c APC	BioLegend	Cat# 117310; RRID: AB_313779
anti-IA/IE Brilliant Violet 510	BioLegend	Cat#107636; RRID: AB_2734168
anti-CD80 PE	BioLegend	Cat# 104708; RRID: AB_313129
anti-CD8α Brilliant Violet 785	BioLegend	Cat# 100750; RRID: AB_2562610
anti-Ly6C PE-Cy7	BioLegend	Cat# 128018; RRID: AB_1732082
anti-F4/80 Brilliant Violet 605	BioLegend	Cat# 123133; RRID: AB_2562305
anti-F4/80 PE Dazzle 594	BioLegend	Cat# 123146; RRID: AB_2564133
anti-CD19 PE	BioLegend	Cat# 152408; RRID: AB_2629817
anti-Gr1 PE-Cy7	BioLegend	Cat# 108416; RRID: AB_313381
anti-CD4 Brilliant Violet 510	BioLegend	Cat# 100559; RRID: AB_2562608
anti-CD69 PE	BioLegend	Cat# 104508; RRID: AB_313111
anti-PD-1 APC	BioLegend	Cat# 135210; RRID: AB_2159183
anti-CD44 Brilliant Violet 421	BioLegend	Cat# 103039; RRID: AB_10895752
anti-CD25 Brilliant Violet 711	BioLegend	Cat# 102049; RRID: AB_2564130
anti-CD45 Brilliant Violet 711	BioLegend	Cat# #103147; RRID: AB_2564383
anti-CD8 APC	BioLegend	Cat# 100712; RRID: AB_312751
anti-IFNγ Brilliant Violet 421	BioLegend	Cat# 505830; RRID: AB_2563105
anti-CD3ε	BioLegend	Cat# 100340; RRID: AB_11149115
IFNγ capture antibody	BD Biosciences	Cat# 551881; RRID: AB_2868948
anti-CD28	BioLegend	Cat# 102116; RRID: AB_11147170
Anti-CD169 PE	BioLegend	Cat# 142403; RRID: AB_10915470
Anti-CD11b BV785	BioLegend	Cat# 101243; RRID: AB_2561373
Anti-CD80 BV711	BioLegend	Cat# 104743; RRID: AB_2810338
Anti-IFNAR1 PE	BioLegend	Cat# 127312; RRID: AB_2248800
Zombie NIR	BioLegend	Cat# 423105
anti-CD45 PE-CF594	BD Biosciences	Cat# 562279; RRID: AB_11154577
anti-CD34 Brilliant Violet 510	BioLegend	Cat# 343528; RRID: AB_2563856
anti-CD20 Pacific Blue	BioLegend	Cat# 302328; RRID: AB_1595435
anti-CD3 Pacific Blue	BioLegend	Cat# 300418; RRID: AB_493095
anti-CD14 Pacific Blue	BioLegend	Cat# 301828; RRID: AB_2275670
anti-CD16 Pacific Blue	BioLegend	Cat# 302032; RRID: AB_2104003
anti-CD19 Pacific Blue	BioLegend	Cat# 302232; RRID: AB_2073118
anti-HLA-DR Brilliant Violet 711	BioLegend	Cat# 307643; RRID: AB_11218794
anti-CD45RA PE-Cy7	BioLegend	Cat#304126; RRID: AB_10708879
anti-CD33 FITC	BioLegend	Cat# 366620; RRID: AB_2566422
anti-CD123 Brilliant Violet 605	BD Biosciences	Cat# 740412; RRID: AB_2740142
anti-CADM1 Alexa Fluor 647	MBL International	Cat# CM004-A64; RRID: AB_3107120
anti-CD1c PE	BioLegend	Cat# 331506; RRID: AB_1088999

Bacterial and virus strains		

DH5α	Life Technologies	Cat# 18265017

Biological samples		

Human PDAC Tissue	This paper	
Human PDAC patient blood	This paper	

Chemicals, peptides, and recombinant proteins		

STING agonist	Bristol Myers Squibb	BMS-986301
BsmBI	NEB	Cat# R0739S
BbsI	NEB	Cat# R3539S
T4 DNA ligase	NEB	Cat# M0202L
Lipofectamine	ThermoFisher	Cat# 13778075
OptiMEM	Life Technologies	Cat# 31985062
Trypsin	Life Technologies	Cat# 25200114
Protease and phosphatase inhibitor	Sigma	Cat# 11836170001
BCA Assay kit	Life Technologies	Cat# 23225
FTY720	Sigma	Cat# SML0700
Dispase	Life Technologies	Cat# 17105041
Collagenase P	Sigma	Cat# 11249002001
DNase I	Sigma	Cat# 10104159001
IFNγ	Peprotech	Cat# 315-05
IFNβ	BioLegend	Cat# 581302
IFNα	BioLegend	Cat# 752804
Qiagen RNAeasy Plus Mini Kit	Qiagen	Cat# 74134
hIL-2	Peprotech	Cat# 200-02
ISQAVHAAHAEINEAGR	in-house generated	
TAPDNLGYM (M9)	Clancy-Thompson et al.^70^	
STEMCELL EasySep CD4 Negative Selection Kit	STEMCELL	Cat# 19852
STEMCELL EasySep CD8 Negative Selection Kit	STEMCELL	Cat# 19853
Brefeldin A	Sigma	Cat# B7651
Collagenase IV	Sigma	Cat# C5138
Soybean trypsin inhbitor	Life Technologies	Cat# 17075029
Tumor dissociation kit	Miltenyi	Cat# 130-095-929

Critical commercial assays		

Micro BCA Kit	Fisher Scientific	Cat# PI23235
Mouse IFNγ ELISpot	Fisher Scientific	Cat# BDB551083
Mouse IFNα ELISA	Life Technologies	Cat# BMS6027

Deposited data		

Loveless et al. data	Loveless et al.^74^	https://zenodo.org/records/14199536
Hwang et al. data	Hwang et al.^75^	GEO# GSE202051
DFCI human single-cell RNA sequencing	This paper	dbGAP: phs004508 dbGAP: phs004257
Mouse single-cell and bulk RNA sequencing	This paper	GEO: GSE325195

Experimental models: Cell lines		

Mouse: 6694c2	Ben Stanger (University of Pennsylvania)	RRID: CVCL_YM31
Mouse: 6419c5	Ben Stanger (University of Pennsylvania)	RRID: CVCL_YM21
Mouse: 6694c2 *β2m*−/−	Roehle et al.^56^	
Mouse: 6694c2 *Ciita*−/−	This paper	
Mouse: 6694c2 *Caspase-8*−/−	This paper	
Mouse: 6694c2COVA	This paper	
Mouse 6694c2 *Stat1*−/−	This paper	
Mouse: 6694c2 *Ifnγr1*−/−	This paper	
Mouse: 6694c2-met	Qiang et al.^58^	

Experimental models: Organisms/strains		

Mouse: C57BL6/J	The Jackson Laboratory	Cat# 000664
Mouse: μMT −/−	The Jackson Laboratory	Cat# 002249
Mouse: *Batf3* −/−	The Jackson Laboratory	Cat# 013755
TCRα −/−	The Jackson Laboratory	Cat# 002116
*β2M* −/−	The Jackson Laboratory	Cat# 002087
*I-Ab* −/−	The Jackson Laboratory	Cat# 005589
*Rag2* −/−	The Jackson Laboratory	Cat# 008449
*Ifnγ* −/−	The Jackson Laboratory	Cat# 002287
*Perforin-1* −/−	The Jackson Laboratory	Cat# 002407
*Ifnar1* −/−	The Jackson Laboratory	Cat# 028288
*Ccr2* −/−	The Jackson Laboratory	Cat# 004999
iNOS −/−	The Jackson Laboratory	Cat# 002609
ΔΔ*Gata1*	The Jackson Laboratory	Cat# 005653
Δ1+2 + 3	The Jackson Laboratory	Cat# 037704
OT-II	The Jackson Laboratory	Cat# 004194
TRP1^high^	Dana-Farber Cancer Institute (bred in-house)	RRID:IMSR_JAX:030958
TCRδ−/−	The Jackson Laboratory	Cat# 002120
*Mr1*−/− TCRδ−/−	Dana-Farber Cancer Institute (bred in-house)	
CD11c^cre^	The Jackson Laboratory	Cat# 008068
LysM^cre^	The Jackson Laboratory	Cat# 004781
MHC-II^fl/fl^	The Jackson Laboratory	Cat# 037709
CD11c^cre^ MHC-II^fl/fl^	Dana-Farber Cancer Institute (bred in-house)	
LysM^cre^ MHC-II^fl/fl^	Dana-Farber Cancer Institute (bred in-house)	
*Tbx21*−/−	The Jackson Laboratory	Cat# 004648

Oligonucleotides		

*Caspase-8* sgRNA	Doench et al.^89^	CAAGAAGCAGGAGACCATCG
*Caspase-8* sgRNA	Doench et al.^89^	ATGATCAGACAGTATCCCCG
*Ciita* sgRNA	Doench et al.^89^	CACCGAGGTCCTTGATTATATCGTG
*Ciita* sgRNA	Doench et al.^89^	CACCGTCCAGTGTCCTAATCTACCA
*Ciita* sgRNA	Doench et al.^89^	CACCGAGCAGGCCAAGACTTACATG
*Ifnγr* sgRNA	Doench et al.^89^	TATGTGGAGCATAACCGGAG
*Ifnγr* sgRNA	Doench et al.^89^	GGTATTCCCAGCATACGACA
*Stat1* sgRNA	Doench et al.^89^	CACCGTTAATGACGAGCTCGTGGAG
*Stat1* sgRNA	Doench et al.^89^	CACCGGGATAGACGCCCAGCCACTG
*Stat1* sgRNA	Doench et al.^89^	CACCGTGTGATGTTAGATAAACAGA
*Stat1* sgRNA	Doench et al.^89^	GAAAAGCAAGCGTAATCTCC

Recombinant DNA		

eGFP-ova-c1 plasmid	Addgene	Cat# 163524
lentiCRIPSPRv2 plasmid	Addgene	Cat# 52961
psPAX2	Addgene	Cat# 12260
pVSV-G	Addgene	Cat# 8454
px459	Addgene	Cat# 62988

Software and algorithms		

R	R Project	RRID:SCR_001905
Cellranger pipeline (v7.0.0)	10× Genomics	RRID:SCR_023221
*Seurat* package (v4.3.0 & v5.3.0)	Hao et al.^90,91^	RRID:SCR_016341
*Harmony* package (v1.2.3)	Korsunsky et al.^92^	RRID:SCR_022206
*DropletUtils* package (v1.18.1)	Griffiths et al.^93^	BioConductor
RStudio		RRID:SCR_000432
ggplot2		RRID:SCR_014601
DESeq2	Love et al.^94^	BioConductor
FlowJo		RRID:SCR_008520
Prism	GraphPad	RRID:SCR_002798

## Highlights

- A triple combination immunotherapy cures pancreatic cancer in mice
- Tumor control is independent of cDC1s, CD8^+^ T cells, and tumor cell MHC expression
- cDC2s prime IFNγ-producing CD4^+^ T cells to control pancreatic tumors
- Human PDAC contains cDC2s and CD4^+^ T cells before and after chemotherapy treatment

## References

[1] Rahib L, Smith BD, Aizenberg R, Rosenzweig AB, Fleshman JM, and Matrisian LM (2014). Projecting cancer incidence and deaths to 2030: the unexpected burden of thyroid, liver, and pancreas cancers in the United States. Cancer Res. 74, 2913–2921. 10.1158/0008-5472.CAN-14-0155. 24840647

[2] Li J, Byrne KT, Yan F, Yamazoe T, Chen Z, Baslan T, Richman LP, Lin JH, Sun YH, Rech AJ, (2018). Tumor Cell-Intrinsic Factors Underlie Heterogeneity of Immune Cell Infiltration and Response to Immunotherapy. Immunity 49, 178–193.e7. 10.1016/j.immuni.2018.06.006. 29958801 PMC6707727

[3] Bayne LJ, Beatty GL, Jhala N, Clark CE, Rhim AD, Stanger BZ, and Vonderheide RH (2012). Tumor-derived granulocyte-macrophage colony-stimulating factor regulates myeloid inflammation and T cell immunity in pancreatic cancer. Cancer Cell 21, 822–835. 10.1016/j.ccr.2012.04.025. 22698406 PMC3575028

[4] Lenehan PJ, Cirella A, Uchida AM, Crowley SJ, Sharova T, Boland G, Dougan M, Dougan SK, and Heckler M (2021). Type 2 immunity is maintained during cancer-associated adipose tissue wasting. Immunother. Adv. 1, ltab011. 10.1093/immadv/ltab011. 34291232 PMC8286632

[5] O’Reilly EM, Oh DY, Dhani N, Renouf DJ, Lee MA, Sun W, Fisher G, Hezel A, Chang SC, Vlahovic G, (2019). Durvalumab With or Without Tremelimumab for Patients With Metastatic Pancreatic Ductal Adenocarcinoma: A Phase 2 Randomized Clinical Trial. JAMA Oncol. 5, 1431–1438. 10.1001/jamaoncol.2019.1588. 31318392 PMC6647002

[6] Brahmer JR, Tykodi SS, Chow LQM, Hwu WJ, Topalian SL, Hwu P, Drake CG, Camacho LH, Kauh J, Odunsi K, (2012). Safety and activity of anti-PD-L1 antibody in patients with advanced cancer. N. Engl. J. Med. 366, 2455–2465. 10.1056/NEJMoa1200694. 22658128 PMC3563263

[7] Royal RE, Levy C, Turner K, Mathur A, Hughes M, Kammula US, Sherry RM, Topalian SL, Yang JC, Lowy I, and Rosenberg SA (2010). Phase 2 trial of single agent Ipilimumab (anti-CTLA-4) for locally advanced or metastatic pancreatic adenocarcinoma. J. Immunother. 33, 828–833. 10.1097/CJI.0b013e3181eec14c. 20842054 PMC7322622

[8] Le DT, Durham JN, Smith KN, Wang H, Bartlett BR, Aulakh LK, Lu S, Kemberling H, Wilt C, Luber BS, (2017). Mismatch repair deficiency predicts response of solid tumors to PD-1 blockade. Science 357, 409–413. 10.1126/science.aan6733. 28596308 PMC5576142

[9] Dougan M, Dranoff G, and Dougan SK (2019). Cancer Immunotherapy: Beyond Checkpoint Blockade. Annu. Rev. Cell Biol. 3, 55–75. 10.1146/annurev-cancerbio-030518-055552. PMC10400018 37539076

[10] Kureshi CT, and Dougan SK (2025). Cytokines in cancer. Cancer Cell 43, 15–35. 10.1016/j.ccell.2024.11.011. 39672170 PMC11841838

[11] Im SJ, Hashimoto M, Gerner MY, Lee J, Kissick HT, Burger MC, Shan Q, Hale JS, Lee J, Nasti TH, (2016). Defining CD8+ T cells that provide the proliferative burst after PD-1 therapy. Nature 537, 417–421. 10.1038/nature19330. 27501248 PMC5297183

[12] Simpson TR, Li F, Montalvo-Ortiz W, Sepulveda MA, Bergerhoff K, Arce F, Roddie C, Henry JY, Yagita H, Wolchok JD, (2013). Fc-dependent depletion of tumor-infiltrating regulatory T cells co-defines the efficacy of anti-CTLA-4 therapy against melanoma. J. Exp. Med. 210, 1695–1710. 10.1084/jem.20130579. 23897981 PMC3754863

[13] Gao J, Shi LZ, Zhao H, Chen J, Xiong L, He Q, Chen T, Roszik J, Bernatchez C, Woodman SE, (2016). Loss of IFN-gamma Pathway Genes in Tumor Cells as a Mechanism of Resistance to Anti-CTLA-4 Therapy. Cell 167, 397–404.e9. 10.1016/j.cell.2016.08.069. 27667683 PMC5088716

[14] Zaretsky JM, Garcia-Diaz A, Shin DS, Escuin-Ordinas H, Hugo W, Hu-Lieskovan S, Torrejon DY, Abril-Rodriguez G, Sandoval S, Barthly L, (2016). Mutations Associated with Acquired Resistance to PD-1 Blockade in Melanoma. N. Engl. J. Med. 375, 819–829. 10.1056/NEJMoa1604958. 27433843 PMC5007206

[15] Shin DS, Zaretsky JM, Escuin-Ordinas H, Garcia-Diaz A, Hu-Lieskovan S, Kalbasi A, Grasso CS, Hugo W, Sandoval S, Torrejon DY, (2017). Primary Resistance to PD-1 Blockade Mediated by JAK1/2 Mutations. Cancer Discov. 7, 188–201. 10.1158/2159-8290.CD-16-1223. 27903500 PMC5296316

[16] Bos R, and Sherman LA (2010). CD4+ T-cell help in the tumor milieu is required for recruitment and cytolytic function of CD8+ T lymphocytes. Cancer Res. 70, 8368–8377. 10.1158/0008-5472.CAN-10-1322. 20940398 PMC2970736

[17] Mumberg D, Monach PA, Wanderling S, Philip M, Toledano AY, Schreiber RD, and Schreiber H (1999). CD4(+) T cells eliminate MHC class II-negative cancer cells in vivo by indirect effects of IFNgamma. Proc. Natl. Acad. Sci. USA 96, 8633–8638. 10.1073/pnas.96.15.8633. 10411927 PMC17568

[18] Hung K, Hayashi R, Lafond-Walker A, Lowenstein C, Pardoll D, and Levitsky H (1998). The central role of CD4(+) T cells in the antitumor immune response. J. Exp. Med. 188, 2357–2368. 10.1084/jem.188.12.2357. 9858522 PMC2212434

[19] Hunder NN, Wallen H, Cao J, Hendricks DW, Reilly JZ, Rodmyre R, Jungbluth A, Gnjatic S, Thompson JA, and Yee C (2008). Treatment of metastatic melanoma with autologous CD4+ T cells against NY-ESO-1. N. Engl. J. Med. 358, 2698–2703. 10.1056/NEJMoa0800251. 18565862 PMC3277288

[20] Quezada SA, Simpson TR, Peggs KS, Merghoub T, Vider J, Fan X, Blasberg R, Yagita H, Muranski P, Antony PA, (2010). Tumor-reactive CD4(+) T cells develop cytotoxic activity and eradicate large established melanoma after transfer into lymphopenic hosts. J. Exp. Med. 207, 637–650. 10.1084/jem.20091918. 20156971 PMC2839156

[21] Tran E, Turcotte S, Gros A, Robbins PF, Lu YC, Dudley ME, Wunderlich JR, Somerville RP, Hogan K, Hinrichs CS, (2014). Cancer immunotherapy based on mutation-specific CD4+ T cells in a patient with epithelial cancer. Science 344, 641–645. 10.1126/science.1251102. 24812403 PMC6686185

[22] Alspach E, Lussier DM, Miceli AP, Kizhvatov I, DuPage M, Luoma AM, Meng W, Lichti CF, Esaulova E, Vomund AN, (2019). MHC-II neoantigens shape tumour immunity and response to immunotherapy. Nature 574, 696–701. 10.1038/s41586-019-1671-8. 31645760 PMC6858572

[23] Corthay A, Skovseth DK, Lundin KU, Røsjø E, Omholt H, Hofgaard PO, Haraldsen G, and Bogen B (2005). Primary antitumor immune response mediated by CD4+ T cells. Immunity 22, 371–383. 10.1016/j.immuni.2005.02.003. 15780993

[24] Perez-Diez A, Joncker NT, Choi K, Chan WFN, Anderson CC, Lantz O, and Matzinger P (2007). CD4 cells can be more efficient at tumor rejection than CD8 cells. Blood 109, 5346–5354. 10.1182/blood-2006-10-051318. 17327412 PMC1890845

[25] Chandra V, Li L, Le Roux O, Zhang Y, Howell RM, Rupani DN, Baydogan S, Miller HD, Riquelme E, Petrosino J, (2024). Gut epithelial Interleukin-17 receptor A signaling can modulate distant tumors growth through microbial regulation. Cancer Cell 42, 85–100.e6. 10.1016/j.ccell.2023.12.006. 38157865 PMC11238637

[26] Hegde S, Krisnawan VE, Herzog BH, Zuo C, Breden MA, Knolhoff BL, Hogg GD, Tang JP, Baer JM, Mpoy C, (2020). Dendritic Cell Paucity Leads to Dysfunctional Immune Surveillance in Pancreatic Cancer. Cancer Cell 37, 289–307.e9. 10.1016/j.ccell.2020.02.008. 32183949 PMC7181337

[27] Thibaut R, Bost P, Milo I, Cazaux M, Lemaıˆtre F, Garcia Z, Amit I, Breart B, Cornuot C, Schwikowski B, and Bousso P (2020). Bystander IFN-gamma activity promotes widespread and sustained cytokine signaling altering the tumor microenvironment. Nat. Cancer 1, 302–314. 10.1038/s43018-020-0038-2. 32803171 PMC7115926

[28] Walsh MJ, Stump CT, Kureshi R, Lenehan P, Ali LR, Dougan M, Knipe DM, and Dougan SK (2023). IFNgamma is a central node of cancer immune equilibrium. Cell Rep. 42, 112219. 10.1016/j.celrep.2023.112219. 36881506 PMC10214249

[29] Kammertoens T, Friese C, Arina A, Idel C, Briesemeister D, Rothe M, Ivanov A, Szymborska A, Patone G, Kunz S, (2017). Tumour ischaemia by interferon-gamma resembles physiological blood vessel regression. Nature 545, 98–102. 10.1038/nature22311. 28445461 PMC5567674

[30] Sun JC, and Bevan MJ (2003). Defective CD8 T cell memory following acute infection without CD4 T cell help. Science 300, 339–342. 10.1126/science.1083317. 12690202 PMC2778341

[31] Ahrends T, Spanjaard A, Pilzecker B, Bąbała N, Bovens A, Xiao Y, Jacobs H, and Borst J (2017). CD4(+) T Cell Help Confers a Cytotoxic T Cell Effector Program Including Coinhibitory Receptor Downregulation and Increased Tissue Invasiveness. Immunity 47, 848–861.e5. 10.1016/j.immuni.2017.10.009. 29126798

[32] Shedlock DJ, and Shen H (2003). Requirement for CD4 T cell help in generating functional CD8 T cell memory. Science 300, 337–339. 10.1126/science.1082305. 12690201

[33] Bourgeois C, Rocha B, and Tanchot C (2002). A role for CD40 expression on CD8+ T cells in the generation of CD8+ T cell memory. Science 297, 2060–2063. 10.1126/science.1072615. 12242444

[34] Janssen EM, Lemmens EE, Wolfe T, Christen U, von Herrath MG, and Schoenberger SP (2003). CD4+ T cells are required for secondary expansion and memory in CD8+ T lymphocytes. Nature 421, 852–856. 10.1038/nature01441. 12594515

[35] Zander R, Schauder D, Xin G, Nguyen C, Wu X, Zajac A, and Cui W (2019). CD4(+) T Cell Help Is Required for the Formation of a Cytolytic CD8(+) T Cell Subset that Protects against Chronic Infection and Cancer. Immunity 51, 1028–1042.e4. 10.1016/j.immuni.2019.10.009. 31810883 PMC6929322

[36] Kruse B, Buzzai AC, Shridhar N, Braun AD, Gellert S, Knauth K, Pozniak J, Peters J, Dittmann P, Mengoni M, (2023). CD4(+) T cell-induced inflammatory cell death controls immune-evasive tumours. Nature 618, 1033–1040. 10.1038/s41586-023-06199-x. 37316667 PMC10307640

[37] Braun DA, Moranzoni G, Chea V, McGregor BA, Blass E, Tu CR, Vanasse AP, Forman C, Forman J, Afeyan AB, (2025). A neoantigen vaccine generates antitumour immunity in renal cell carcinoma. Nature 639, 474–482. 10.1038/s41586-024-08507-5. 39910301 PMC11903305

[38] Ott PA, Hu Z, Keskin DB, Shukla SA, Sun J, Bozym DJ, Zhang W, Luoma A, Giobbie-Hurder A, Peter L, (2017). An immunogenic personal neoantigen vaccine for patients with melanoma. Nature 547, 217–221. 10.1038/nature22991. 28678778 PMC5577644

[39] Ferris ST, Durai V, Wu R, Theisen DJ, Ward JP, Bern MD, Davidson JT 4th, Bagadia P, Liu T, Briseño CG, (2020). cDC1 prime and are licensed by CD4(+) T cells to induce anti-tumour immunity. Nature 584, 624–629. 10.1038/s41586-020-2611-3. 32788723 PMC7469755

[40] Cohen M, Giladi A, Barboy O, Hamon P, Li B, Zada M, Gurevich-Shapiro A, Beccaria CG, David E, Maier BB, (2022). The interaction of CD4(+) helper T cells with dendritic cells shapes the tumor microenvironment and immune checkpoint blockade response. Nat. Cancer 3, 303–317. 10.1038/s43018-022-00338-5. 35241835

[41] Broz ML, Binnewies M, Boldajipour B, Nelson AE, Pollack JL, Erle DJ, Barczak A, Rosenblum MD, Daud A, Barber DL, (2014). Dissecting the Tumor Myeloid Compartment Reveals Rare Activating Antigen-Presenting Cells Critical for T Cell Immunity. Cancer Cell 26, 938. 10.1016/j.ccell.2014.11.010. 28898680

[42] Duong E, Fessenden TB, Lutz E, Dinter T, Yim L, Blatt S, Bhutkar A, Wittrup KD, and Spranger S (2022). Type I interferon activates MHC class I-dressed CD11b(+) conventional dendritic cells to promote protective anti-tumor CD8(+) T cell immunity. Immunity 55, 308–323.e9. 10.1016/j.immuni.2021.10.020. 34800368 PMC10827482

[43] Prokhnevska N, Cardenas MA, Valanparambil RM, Sobierajska E, Barwick BG, Jansen C, Reyes Moon A, Gregorova P, delBalzo L, Greenwald R, (2023). CD8(+) T cell activation in cancer comprises an initial activation phase in lymph nodes followed by effector differentiation within the tumor. Immunity 56, 107–124.e5. 10.1016/j.immuni.2022.12.002. 36580918 PMC10266440

[44] Binnewies M, Mujal AM, Pollack JL, Combes AJ, Hardison EA, Barry KC, Tsui J, Ruhland MK, Kersten K, Abushawish MA, (2019). Unleashing Type-2 Dendritic Cells to Drive Protective Antitumor CD4(+) T Cell Immunity. Cell 177, 556–571.e16. 10.1016/j.cell.2019.02.005. 30955881 PMC6954108

[45] James CA, Baer JM, Zou C, Panni UY, Knolhoff BL, Hogg GD, Kingston NL, Kang LI, Lander VE, Luo J, (2023). Systemic Alterations in Type-2 Conventional Dendritic Cells Lead to Impaired Tumor Immunity in Pancreatic Cancer. Cancer Immunol. Res. 11, 1055–1067. 10.1158/2326-6066.CIR-21-0946. 37229629 PMC10524961

[46] Lin JH, Huffman AP, Wattenberg MM, Walter DM, Carpenter EL, Feldser DM, Beatty GL, Furth EE, and Vonderheide RH (2020). Type 1 conventional dendritic cells are systemically dysregulated early in pancreatic carcinogenesis. J. Exp. Med. 217, e20190673. 10.1084/jem.20190673. 32453421 PMC7398166

[47] Burrack AL, Schmiechen ZC, Patterson MT, Miller EA, Spartz EJ, Rollins MR, Raynor JF, Mitchell JS, Kaisho T, Fife BT, and Stromnes IM (2022). Distinct myeloid antigen-presenting cells dictate differential fates of tumor-specific CD8+ T cells in pancreatic cancer. JCI Insight 7, e151593. 10.1172/jci.insight.151593. 35393950 PMC9057584

[48] Ishikawa H, and Barber GN (2008). STING is an endoplasmic reticulum adaptor that facilitates innate immune signalling. Nature 455, 674–678. 10.1038/nature07317. 18724357 PMC2804933

[49] Ishikawa H, Ma Z, and Barber GN (2009). STING regulates intracellular DNA-mediated, type I interferon-dependent innate immunity. Nature 461, 788–792. 10.1038/nature08476. 19776740 PMC4664154

[50] Elewaut A, Estivill G, Bayerl F, Castillon L, Novatchkova M, Pottendorfer E, Hoffmann-Haas L, Schönlein M, Nguyen TV, Lauss M, (2025). Cancer cells impair monocyte-mediated T cell stimulation to evade immunity. Nature 637, 716–725. 10.1038/s41586-024-08257-4. 39604727 PMC7617236

[51] Jing W, McAllister D, Vonderhaar EP, Palen K, Riese MJ, Gershan J, Johnson BD, and Dwinell MB (2019). STING agonist inflames the pancreatic cancer immune microenvironment and reduces tumor burden in mouse models. J. Immunother. Cancer 7, 115. 10.1186/s40425-019-0573-5. 31036082 PMC6489306

[52] Marcus A, Mao AJ, Lensink-Vasan M, Wang L, Vance RE, and Raulet DH (2018). Tumor-Derived cGAMP Triggers a STING-Mediated Interferon Response in Non-tumor Cells to Activate the NK Cell Response. Immunity 49, 754–763.e4. 10.1016/j.immuni.2018.09.016. 30332631 PMC6488306

[53] Nicolai CJ, Wolf N, Chang IC, Kirn G, Marcus A, Ndubaku CO, McWhirter SM, and Raulet DH (2020). NK cells mediate clearance of CD8(+) T cell-resistant tumors in response to STING agonists. Sci. Immunol. 5, eaaz2738. 10.1126/sciimmunol.aaz2738. 32198222 PMC7228660

[54] Vonderhaar EP, Barnekow NS, McAllister D, McOlash L, Eid MA, Riese MJ, Tarakanova VL, Johnson BD, and Dwinell MB (2021). STING Activated Tumor-Intrinsic Type I Interferon Signaling Promotes CXCR3 Dependent Antitumor Immunity in Pancreatic Cancer. Cell. Mol. Gastroenterol. Hepatol. 12, 41–58. 10.1016/j.jcmgh.2021.01.018. 33548597 PMC8081932

[55] Hu J, Sánchez-Rivera FJ, Wang Z, Johnson GN, Ho YJ, Ganesh K, Umeda S, Gan S, Mujal AM, Delconte RB, (2023). STING inhibits the reactivation of dormant metastasis in lung adenocarcinoma. Nature 616, 806–813. 10.1038/s41586-023-05880-5. 36991128 PMC10569211

[56] Roehle K, Qiang L, Ventre KS, Heid D, Ali LR, Lenehan P, Heckler M, Crowley SJ, Stump CT, Ro G, (2021). cIAP1/2 antagonism eliminates MHC class I-negative tumors through T cell-dependent reprogramming of mononuclear phagocytes. Sci. Transl. Med. 13, eabf5058. 10.1126/scitranslmed.abf5058. 34011631 PMC8406785

[57] Stump CT, Roehle K, Manjarrez Orduno N, and Dougan SK (2021). Radiation combines with immune checkpoint blockade to enhance T cell priming in a murine model of poorly immunogenic pancreatic cancer. Open Biol. 11, 210245. 10.1098/rsob.210245. 34784792 PMC8595997

[58] Qiang L, Hoffman MT, Ali LR, Castillo JI, Kageler L, Temesgen A, Lenehan P, Wang SJ, Bello E, Cardot-Ruffino V, (2023). Transforming Growth Factor-beta Blockade in Pancreatic Cancer Enhances Sensitivity to Combination Chemotherapy. Gastroenterology 165, 874–890.e10. 10.1053/j.gastro.2023.05.038. 37263309 PMC10526623

[59] Li S, Mirlekar B, Johnson BM, Brickey WJ, Wrobel JA, Yang N, Song D, Entwistle S, Tan X, Deng M, (2022). STING-induced regulatory B cells compromise NK function in cancer immunity. Nature 610, 373–380. 10.1038/s41586-022-05254-3. 36198789 PMC9875944

[60] Mirlekar B, Wang Y, Li S, Zhou M, Entwistle S, De Buysscher T, Morrison A, Herrera G, Harris C, Vincent BG, (2022). Balance between immunoregulatory B cells and plasma cells drives pancreatic tumor immunity. Cell Rep. Med. 3, 100744. 10.1016/j.xcrm.2022.100744. 36099917 PMC9512696

[61] Chin YE, Kitagawa M, Su WC, You ZH, Iwamoto Y, and Fu XY (1996). Cell growth arrest and induction of cyclin-dependent kinase inhibitor p21 WAF1/CIP1 mediated by STAT1. Science 272, 719–722. 10.1126/science.272.5262.719. 8614832

[62] Braumüller H, Wieder T, Brenner E, Aßmann S, Hahn M, Alkhaled M, Schilbach K, Essmann F, Kneilling M, Griessinger C, (2013). T-helper-1-cell cytokines drive cancer into senescence. Nature 494, 361–365. 10.1038/nature11824. 23376950

[63] Lord GM, Rao RM, Choe H, Sullivan BM, Lichtman AH, Luscinskas FW, and Glimcher LH (2005). T-bet is required for optimal proinflammatory CD4+ T-cell trafficking. Blood 106, 3432–3439. 10.1182/blood-2005-04-1393. 16014561 PMC1895048

[64] de Vries NL, van de Haar J, Veninga V, Chalabi M, Ijsselsteijn ME, van der Ploeg M, van den Bulk J, Ruano D, van den Berg JG, Haanen JB, (2023). gammadelta T cells are effectors of immunotherapy in cancers with HLA class I defects. Nature 613, 743–750. 10.1038/s41586-022-05593-1. 36631610 PMC9876799

[65] Fransen MF, Schoonderwoerd M, Knopf P, Camps MG, Hawinkels LJ, Kneilling M, van Hall T, and Ossendorp F (2018). Tumor-draining lymph nodes are pivotal in PD-1/PD-L1 checkpoint therapy. JCI Insight 3, e124507. 10.1172/jci.insight.124507. 30518694 PMC6328025

[66] Roberts EW, Broz ML, Binnewies M, Headley MB, Nelson AE, Wolf DM, Kaisho T, Bogunovic D, Bhardwaj N, and Krummel MF (2016). Critical Role for CD103(+)/CD141(+) Dendritic Cells Bearing CCR7 for Tumor Antigen Trafficking and Priming of T Cell Immunity in Melanoma. Cancer Cell 30, 324–336. 10.1016/j.ccell.2016.06.003. 27424807 PMC5374862

[67] Salmon H, Idoyaga J, Rahman A, Leboeuf M, Remark R, Jordan S, Casanova-Acebes M, Khudoynazarova M, Agudo J, Tung N, (2016). Expansion and Activation of CD103(+) Dendritic Cell Progenitors at the Tumor Site Enhances Tumor Responses to Therapeutic PD-L1 and BRAF Inhibition. Immunity 44, 924–938. 10.1016/j.immuni.2016.03.012. 27096321 PMC4980762

[68] Liu TT, Kim S, Desai P, Kim DH, Huang X, Ferris ST, Wu R, Ou F, Egawa T, Van Dyken SJ, (2022). Ablation of cDC2 development by triple mutations within the Zeb2 enhancer. Nature 607, 142–148. 10.1038/s41586-022-04866-z. 35732734 PMC10358283

[69] Dougan SK, Dougan M, Kim J, Turner JA, Ogata S, Cho HI, Jaenisch R, Celis E, and Ploegh HL (2013). Transnuclear TRP1-specific CD8 T cells with high or low affinity TCRs show equivalent antitumor activity. Cancer Immunol. Res. 1, 99–111. 10.1158/2326-6066.CIR-13-0047. 24459675 PMC3895912

[70] Clancy-Thompson E, Devlin CA, Tyler PM, Servos MM, Ali LR, Ventre KS, Bhuiyan MA, Bruck PT, Birnbaum ME, and Dougan SK (2018). Altered Binding of Tumor Antigenic Peptides to MHC Class I Affects CD8(+) T Cell-Effector Responses. Cancer Immunol. Res. 6, 1524–1536. 10.1158/2326-6066.CIR-18-0348. 30352798 PMC6290996

[71] Wang X, Huang Z, Xing L, Shang L, Jiang J, Deng C, Yu W, Peng L, Yang H, Zheng X, (2025). STING agonist-based ER-targeting molecules boost antigen cross-presentation. Nature 641, 202–210. 10.1038/s41586-025-08758-w. 40140567 PMC12043507

[72] Sohal DPS, Kennedy EB, Khorana A, Copur MS, Crane CH, Garrido-Laguna I, Krishnamurthi S, Moravek C, O’Reilly EM, Philip PA, (2018). Metastatic Pancreatic Cancer: ASCO Clinical Practice Guideline Update. J. Clin. Oncol. 36, 2545–2556. 10.1200/JCO.2018.78.9636. 29791286 PMC7504972

[73] Posta M, and Györffy B (2023). Analysis of a large cohort of pancreatic cancer transcriptomic profiles to reveal the strongest prognostic factors. Clin. Transl. Sci. 16, 1479–1491. 10.1111/cts.13563. 37260110 PMC10432876

[74] Loveless IM, Kemp SB, Hartway KM, Mitchell JT, Wu Y, Zwernik SD, Salas-Escabillas DJ, Brender S, George M, Makinwa Y, (2025). Human Pancreatic Cancer Single-Cell Atlas Reveals Association of CXCL10+ Fibroblasts and Basal Subtype Tumor Cells. Clin. Cancer Res. 31, 756–772. 10.1158/1078-0432.CCR-24-2183. 39636224 PMC11831110

[75] Hwang WL, Jagadeesh KA, Guo JA, Hoffman HI, Yadollahpour P, Reeves JW, Mohan R, Drokhlyansky E, Van Wittenberghe N, Ashenberg O, (2022). Single-nucleus and spatial transcriptome profiling of pancreatic cancer identifies multicellular dynamics associated with neoadjuvant treatment. Nat. Genet. 54, 1178–1191. 10.1038/s41588-022-01134-8. 35902743 PMC10290535

[76] Hildner K, Edelson BT, Purtha WE, Diamond M, Matsushita H, Kohyama M, Calderon B, Schraml BU, Unanue ER, Diamond MS, (2008). Batf3 deficiency reveals a critical role for CD8alpha+ dendritic cells in cytotoxic T cell immunity. Science 322, 1097–1100. 10.1126/science.1164206. 19008445 PMC2756611

[77] Cardot-Ruffino V, Bollenrucher N, Delius L, Wang SJ, Brais LK, Remland J, Keheler CE, Sullivan KM, Abrams TA, Biller LH, (2023). G-CSF rescue of FOLFIRINOX-induced neutropenia leads to systemic immune suppression in mice and humans. J. Immunother. Cancer 11, e006589. 10.1136/jitc-2022-006589. 37344102 PMC10314699

[78] Dias Costa A, Väyrynen SA, Chawla A, Zhang J, Väyrynen JP, Lau MC, Williams HL, Yuan C, Morales-Oyarvide V, Elganainy D, (2022). Neoadjuvant Chemotherapy Is Associated with Altered Immune Cell Infiltration and an Anti-Tumorigenic Microenvironment in Resected Pancreatic Cancer. Clin. Cancer Res. 28, 5167–5179. 10.1158/1078-0432.CCR-22-1125. 36129461 PMC9999119

[79] Oberstein PE, Dias Costa A, Kawaler EA, Cardot-Ruffino V, Rahma OE, Beri N, Singh H, Abrams TA, Biller LH, Cleary JM, (2024). Blockade of IL1beta and PD1 with Combination Chemotherapy Reduces Systemic Myeloid Suppression in Metastatic Pancreatic Cancer with Heterogeneous Effects in the Tumor. Cancer Immunol. Res. 12, 1221–1235. 10.1158/2326-6066.CIR-23-1073. 38990554 PMC11369625

[80] Beatty GL, Chiorean EG, Fishman MP, Saboury B, Teitelbaum UR, Sun W, Huhn RD, Song W, Li D, Sharp LL, (2011). CD40 agonists alter tumor stroma and show efficacy against pancreatic carcinoma in mice and humans. Science 331, 1612–1616. 10.1126/science.1198443. 21436454 PMC3406187

[81] Long KB, Gladney WL, Tooker GM, Graham K, Fraietta JA, and Beatty GL (2016). IFNgamma and CCL2 Cooperate to Redirect Tumor-Infiltrating Monocytes to Degrade Fibrosis and Enhance Chemotherapy Efficacy in Pancreatic Carcinoma. Cancer Discov. 6, 400–413. 10.1158/2159-8290.CD-15-1032. 26896096 PMC4843521

[82] Van Laethem JL, Borbath I, Prenen H, Geboes KP, Lambert A, Mitry E, Cassier PA, Blanc JF, Pilla L, Batlle JF, (2024). Combining CD40 agonist mitazalimab with mFOLFIRINOX in previously untreated metastatic pancreatic ductal adenocarcinoma (OPTIMIZE-1): a single-arm, multicentre phase 1b/2 study. Lancet Oncol. 25, 853–864. 10.1016/S1470-2045(24)00263-8. 38834087

[83] Morrison AH, Diamond MS, Hay CA, Byrne KT, and Vonderheide RH (2020). Sufficiency of CD40 activation and immune checkpoint blockade for T cell priming and tumor immunity. Proc. Natl. Acad. Sci. USA 117, 8022–8031. 10.1073/pnas.1918971117. 32213589 PMC7149500

[84] Ali LR, Lenehan PJ, Cardot-Ruffino V, Dias Costa A, Katz MHG, Bauer TW, Nowak JA, Wolpin BM, Abrams TA, Patel A, (2024). PD-1 Blockade Induces Reactivation of Nonproductive T-Cell Responses Characterized by NF-kappaB Signaling in Patients with Pancreatic Cancer. Clin. Cancer Res. 30, 542–553. 10.1158/1078-0432.CCR-23-1444. 37733830 PMC10831338

[85] Balachandran VP, Łuksza M, Zhao JN, Makarov V, Moral JA, Remark R, Herbst B, Askan G, Bhanot U, Senbabaoglu Y, (2017). Identification of unique neoantigen qualities in long-term survivors of pancreatic cancer. Nature 551, 512–516. 10.1038/nature24462. 29132146 PMC6145146

[86] Evans RA, Diamond MS, Rech AJ, Chao T, Richardson MW, Lin JH, Bajor DL, Byrne KT, Stanger BZ, Riley JL, (2016). Lack of immunoediting in murine pancreatic cancer reversed with neoantigen. JCI Insight 1, e88328. 10.1172/jci.insight.88328. 27642636 PMC5026128

[87] Pant S, Wainberg ZA, Weekes CD, Furqan M, Kasi PM, Devoe CE, Leal AD, Chung V, Basturk O, VanWyk H, (2024). Lymph-node-targeted, mKRAS-specific amphiphile vaccine in pancreatic and colorectal cancer: the phase 1 AMPLIFY-201 trial. Nat. Med. 30, 531–542. 10.1038/s41591-023-02760-3. 38195752 PMC10878978

[88] Rojas LA, Sethna Z, Soares KC, Olcese C, Pang N, Patterson E, Lihm J, Ceglia N, Guasp P, Chu A, (2023). Personalized RNA neoantigen vaccines stimulate T cells in pancreatic cancer. Nature 618, 144–150. 10.1038/s41586-023-06063-y. 37165196 PMC10171177

[89] Doench JG, Fusi N, Sullender M, Hegde M, Vaimberg EW, Donovan KF, Smith I, Tothova Z, Wilen C, Orchard R, (2016). Optimized sgRNA design to maximize activity and minimize off-target effects of CRISPR-Cas9. Nat. Biotechnol. 34, 184–191. 10.1038/nbt.3437. 26780180 PMC4744125

[90] Hao Y, Hao S, Andersen-Nissen E, Mauck WM 3rd, Zheng S, Butler A, Lee MJ, Wilk AJ, Darby C, Zager M, (2021). Integrated analysis of multimodal single-cell data. Cell 184, 3573–3587.e29. 10.1016/j.cell.2021.04.048. 34062119 PMC8238499

[91] Hao Y, Stuart T, Kowalski MH, Choudhary S, Hoffman P, Hartman A, Srivastava A, Molla G, Madad S, Fernandez-Granda C, and Satija R (2024). Dictionary learning for integrative, multimodal and scalable single-cell analysis. Nat. Biotechnol. 42, 293–304. 10.1038/s41587-023-01767-y. 37231261 PMC10928517

[92] Korsunsky I, Millard N, Fan J, Slowikowski K, Zhang F, Wei K, Baglaenko Y, Brenner M, Loh PR, and Raychaudhuri S (2019). Fast, sensitive and accurate integration of single-cell data with Harmony. Nat. Methods 16, 1289–1296. 10.1038/s41592-019-0619-0. 31740819 PMC6884693

[93] Griffiths JA, Richard AC, Bach K, Lun ATL, and Marioni JC (2018). Detection and removal of barcode swapping in single-cell RNA-seq data. Nat. Commun. 9, 2667. 10.1038/s41467-018-05083-x. 29991676 PMC6039488

[94] Love MI, Huber W, and Anders S (2014). Moderated estimation of fold change and dispersion for RNA-seq data with DESeq2. Genome Biol. 15, 550. 10.1186/s13059-014-0550-8. 25516281 PMC4302049

[95] Maruyama T, Dougan SK, Truttmann MC, Bilate AM, Ingram JR, and Ploegh HL (2015). Increasing the efficiency of precise genome editing with CRISPR-Cas9 by inhibition of nonhomologous end joining. Nat. Biotechnol. 33, 538–542. 10.1038/nbt.3190. 25798939 PMC4618510

[96] Cherney EC, Zhang L, Lo J, Huynh T, Wei D, Ahuja V, Quesnelle C, Schieven GL, Futran A, Locke GA, (2022). Discovery of Non-Nucleotide Small-Molecule STING Agonists via Chemotype Hybridization. J. Med. Chem. 65, 3518–3538. 10.1021/acs.jmedchem.1c01986. 35108011

[97] Fletcher AL, Malhotra D, Acton SE, Lukacs-Kornek V, Bellemare-Pelletier A, Curry M, Armant M, and Turley SJ (2011). Reproducible isolation of lymph node stromal cells reveals site-dependent differences in fibroblastic reticular cells. Front. Immunol. 2, 35. 10.3389/fimmu.2011.00035. 22566825 PMC3342056

[98] Kureshi R, Bello E, Kureshi CTS, Walsh MJ, Lippert V, Hoffman MT, Dougan M, Longmire T, Wichroski M, and Dougan SK (2023). DGKalpha/zeta inhibition lowers the TCR affinity threshold and potentiates antitumor immunity. Sci. Adv. 9, eadk1853. 10.1126/sciadv.adk1853. 38000024 PMC10672170

[99] Satija R, Farrell JA, Gennert D, Schier AF, and Regev A (2015). Spatial reconstruction of single-cell gene expression data. Nat. Biotechnol. 33, 495–502. 10.1038/nbt.3192. 25867923 PMC4430369

[100] Stuart T, Butler A, Hoffman P, Hafemeister C, Papalexi E, Mauck WM, Hao Y, Stoeckius M, Smibert P, and Satija R (2019). Comprehensive Integration of Single-Cell Data. Cell 177, 1888–1902.e21. 10.1016/j.cell.2019.05.031. 31178118 PMC6687398

