# Supplementary material for: Dendritic cell redundancy enables priming of anti-tumor CD4+ T cells in pancreatic cancer
Source: Cancer Cell. Author manuscript; Available in PMC 2026 Aug 3. (PMC13430408; doi:10.1016/j.ccell.2026.04.005)
Supplement: Supplemental Figures [file NIHMS2192170-supplement-Supplemental_Figures.pdf]

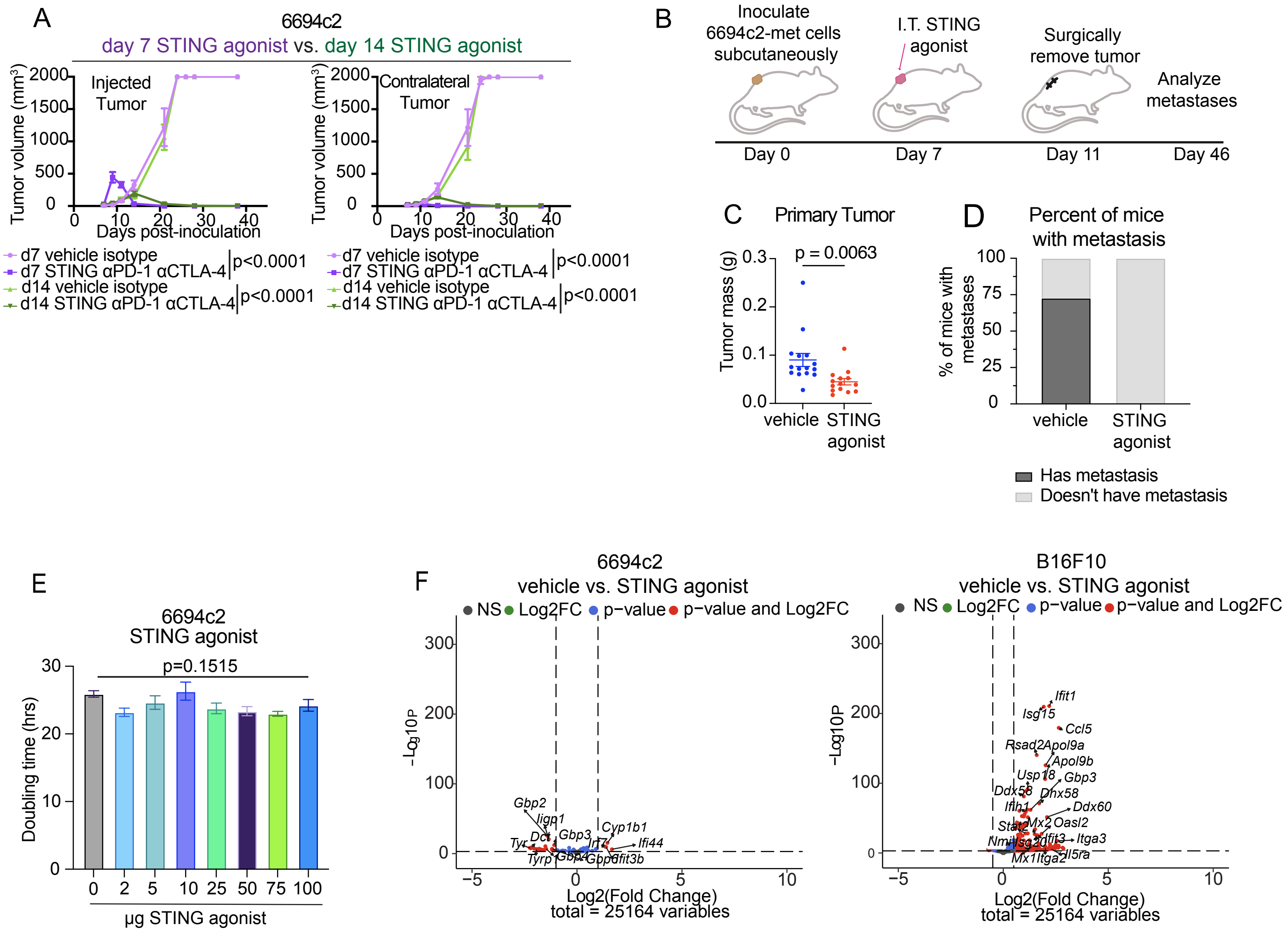

Figure S1. Triple combination therapy effectively controls large tumors and prevents metastasis, and the STING agonist does not act in a tumor cell-intrinsic manner, related to Figure 1.

(A) In vivo tumor growth curves of WT mice inoculated with bilateral subcutaneous 6694c2 tumors injected with STING agonist or vehicle on day 7 post-inoculation (purple) or day 14 post-inoculation (green). N=6 for vehicle-treated groups and N=7 for STING agonist anti-PD-1 anti-CTLA-4-treated groups. 7/7 mice cured in both triple combination therapy-treated groups. (B) Experimental design of the experiments shown in panels C-D. Mice were inoculated with subcutaneous 6694c2-met tumors. Seven days later, the tumor was injected with 100 µg STING agonist or vehicle. Four days later, the tumor was surgically removed in a survival surgery. Thirty-five days later, mice were sacrificed and lungs and lymph nodes were harvested to examine and count metastases. (C) Bar graph showing primary tumor mass of tumors removed on day 11 post-inoculation (four days post-STING agonist). (D) Bar graph showing percent of mice with (black) and without (grey) metastases. N=15 vehicle- and N=14 STING agonist-treated mice. Statistical significance of tumor growth curves determined by calculating area under the curve in GraphPad Prism and conducting T-tests of areas (error bars report SEM). Other p values were calculated by performing T-tests (error bars report SD). (E) Bar graph of 6694c2 doubling time at varying STING agonist concentrations. 6694c2 cells were incubated with varying concentrations of STING agonist at 37°C over five days. Tumor cell confluence was measured with a Celigo imaging cytometer. P value was determined via T-test (error bars report SD). (F) Volcano plot showing differentially expressed genes in 6694c2 cells and B16F10 cells cultured with vehicle and STING agonist. Tumor cells were cultured with 225 µg/mL STING agonist or vehicle for 24 hours. RNA was isolated and bulk RNA sequencing conducted. Illustration from NIAID NIH BioArt Source: bioart.niaid.nih.gov/bioart/591.

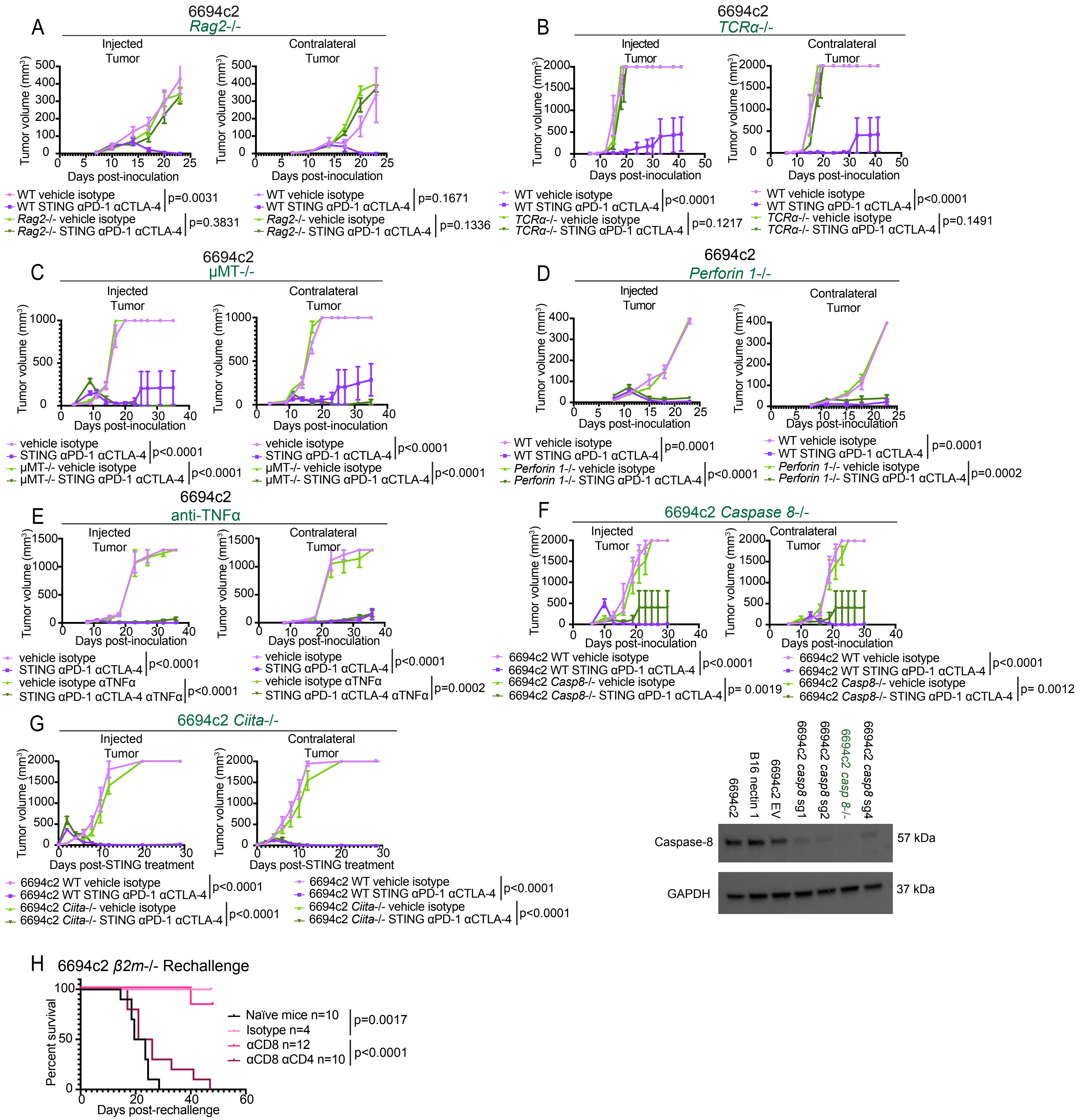

Figure S2. T cells, but not conventional CD8+ T cell cytolytic mechanisms, are required for in vivo pancreatic tumor control, related to Figures 1-2. 6694c2 tumor growth in WT (purple) and (A) *Rag2*-/-, (B) *Tcrα*-/-, (C) µMT, and (D) *Perforin 1*-/- (green) mice. Mice were inoculated with bilateral 6694c2 tumors, and seven days later one tumor was injected with intratumoral STING agonist or vehicle. Anti-PD-1 and anti-CTLA-4 or isotypes were delivered systemically (as in Figure 1A). Tumor growth was measured every 2-3 days. (E) 150 µg anti-TNFα antibody was administered to WT mice every 2-3 days starting on day 5 after inoculation. N=5 mice per group except *perforin 1*-/- vehicle group, where N=4. (A) 5/5 WT mice cured, 0/5 *Rag2*-/- mice cured. (B) 3/5 WT mice cured, 0/5 *Tcrα*-/- mice cured. (C) 1/5 WT mice and 3/5 µMT mice cured. (D) 1/5 WT and 0/5 *Perforin-1*-/- mice cured. (E) 1/5 control and 0/5 anti-TNFα-treated mice cured. (F) 4/5 6694c2 WT- and 4/5 6694c2 *caspase-8*-/--bearing mice cured. (G) 5/5 mice bearing 6694c2 *Ciita*-/- and 3/5 mice bearing 6694c2 tumors cured. (F, bottom) 6694c2 WT, B16 nectin-1, 6694c2 empty vector, and four 6694c2 lines transduced with caspase-8 single-guide RNAs were lysed. Cell line colored green is used in experiments. (H) Survival curve of rechallenged mice that had previously rejected 6694c2 *β2m*-/- tumors. Mice received 200 µg anti-CD8 +/- anti-CD4 or isotype twice (separated by 3 days in between doses) prior to rechallenge. Significance determined via the Log-Rank (Mantel-Cox) test. Tumor growth curve significance was determined by calculating AUC in GraphPad Prism and conducting T-tests of areas (error bars report SEM).

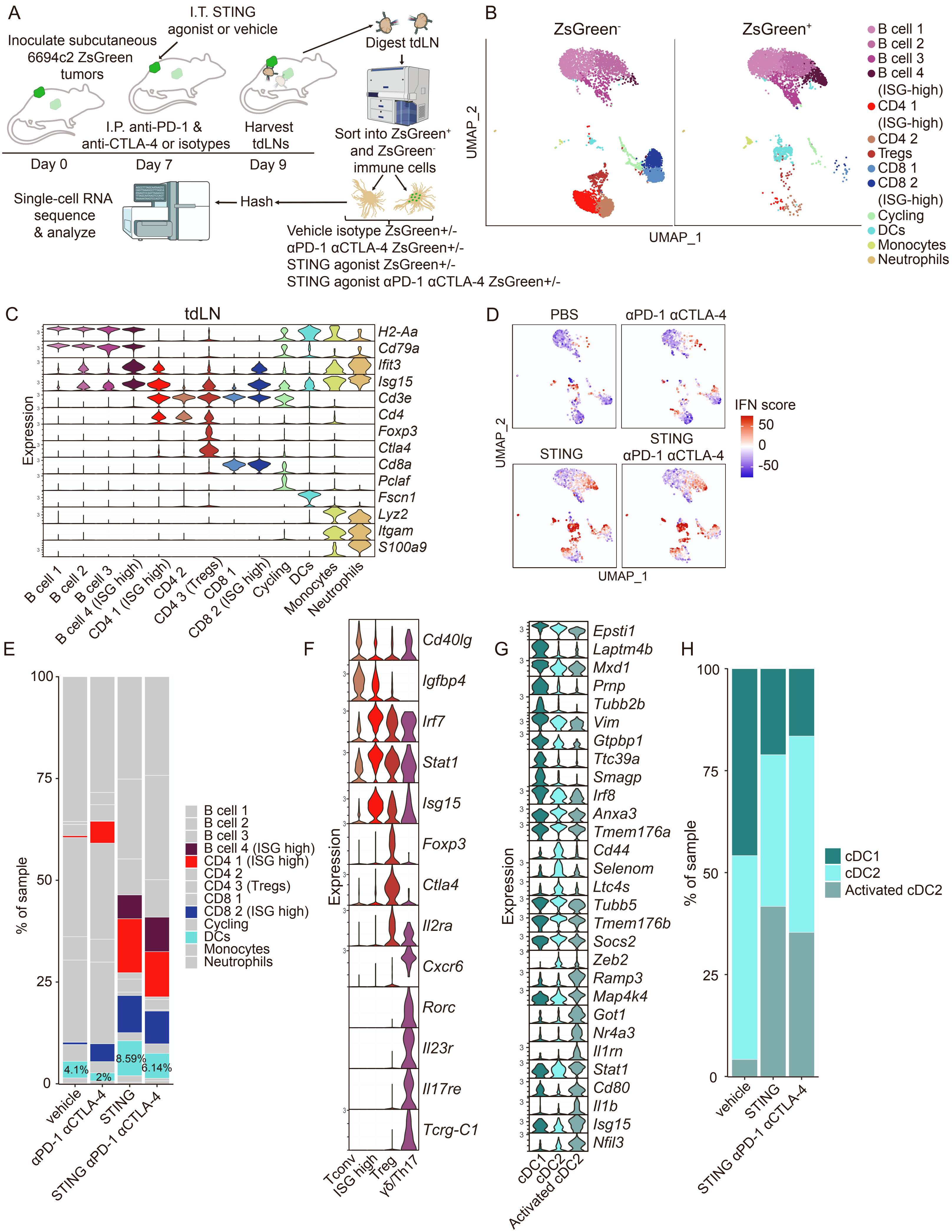
A
I.T. STING agonist or vehicle
Inoculate subcutaneous 6694c2 ZsGreen tumors
Digest tdLN
I.P. anti-PD-1 & anti-CTLA-4 or isotypes
Harvest tdLNs
Sort into ZsGreen+ and ZsGreen- immune cells
Day 0
Day 7
Day 9
Single-cell RNA sequence & analyze
Hash
Vehicle isotype ZsGreen+/-
αPD-1 αCTLA-4 ZsGreen+/-
STING agonist ZsGreen+/-
STING agonist αPD-1 αCTLA-4 ZsGreen+/-
B
ZsGreen-
ZsGreen+
UMAP_2
UMAP_1
B cell 1
B cell 2
B cell 3
B cell 4 (ISG-high)
CD4 1 (ISG-high)
CD4 2
Tregs
CD8 1
CD8 2 (ISG-high)
Cycling
DCs
Monocytes
Neutrophils
C
tdLN
Expression
H2-Aa
Cd79a
Ifit3
Isg15
Cd3e
Cd4
Foxp3
Ctla4
Cd8a
Pclaf
Fscn1
Lyz2
Itgam
S100a9
B cell 1
B cell 2
B cell 3
B cell 4 (ISG high)
CD4 1 (ISG high)
CD4 2
CD4 3 (Tregs)
CD8 1
CD8 2 (ISG high)
Cycling
DCs
Monocytes
Neutrophils
D
PBS
αPD-1 αCTLA-4
STING
STING αPD-1 αCTLA-4
UMAP_2
UMAP_1
IFN score
50
0
-50
E
% of sample
100
75
50
25
0
4.1%
2%
8.59%
6.14%
vehicle
αPD-1 αCTLA-4
STING
STING αPD-1 αCTLA-4
B cell 1
B cell 2
B cell 3
B cell 4 (ISG high)
CD4 1 (ISG high)
CD4 2
CD4 3 (Tregs)
CD8 1
CD8 2 (ISG high)
Cycling
DCs
Monocytes
Neutrophils
F
Expression
Cd40lg
Igfbp4
Irf7
Stat1
Isg15
Foxp3
Ctla4
Il2ra
Cxcr6
Rorc
Il23r
Il17re
Tcrg-C1
Tconv
ISG high
Treg
γδ/Th17
G
Expression
Epsti1
Laptm4b
Mxd1
Prnp
Tubb2b
Vim
Gtpbp1
Ttc39a
Smagp
Irf8
Anxa3
Tmem176a
Cd44
Selenom
Ltc4s
Tubb5
Tmem176b
Socs2
Zeb2
Ramp3
Map4k4
Got1
Nr4a3
Il1rn
Stat1
Cd80
Il1b
Isg15
Nfil3
cDC1
cDC2
Activated cDC2
H
% of sample
100
75
50
25
0
vehicle
STING
STING αPD-1 αCTLA-4
cDC1
cDC2
Activated cDC2

Figure S3. CD4$^+$ T cells and dendritic cells are present and express interferon-response signatures in tumor-draining lymph nodes of responding mice, related to Figures 2 and 3.

(A) Experimental design of panels B-H, and in Figures 2E and 3H-I. Mice were inoculated with 6694c2 ZsGreen tumor cells. Seven days post-inoculation, mice were treated with STING agonist anti-PD-1 and anti-CTLA-4, STING agonist and isotypes, vehicle and anti-PD-1 and anti-CTLA-4, or vehicle and isotypes. 48 hours post-treatment, tumor-draining lymph nodes were harvested and digested to a single-cell suspension. They were then sorted into ZsGreen$^+$ and ZsGreen$^-$ immune cells, hashed, and analyzed via single-cell RNA sequencing. N=5 mice per group. (B) UMAP of analyzed cells from the tumor-draining lymph nodes, split by flow cytometry ZsGreen positivity. (C) Violin plots showing cluster-defining genes among the cells recovered from the tumor-draining lymph nodes. (D) Interferon score of the different cell types recovered from the lymph node on the UMAP. (E) Proportions of the different interferon-stimulated cell types in the lymph node by treatment. Cells with interferon-response signatures are colored. (F) Violin plot examining the gene expression of CD4 T cell populations found in the tumor-draining lymph node. (G) Violin plot showing gene expression profiles of dendritic cell populations found the tumor-draining lymph nodes. (H) Proportions of different dendritic cell populations in the lymph node by treatment. Illustrations from NIAID NIH BioArt Source: bioart.niaid.nih.gov/bioart/591; bioart.niaid.nih.gov/bioart/304; https://bioart.niaid.nih.gov/bioart/160; https://bioart.niaid.nih.gov/bioart/116; and https://bioart.niaid.nih.gov/bioart/386.

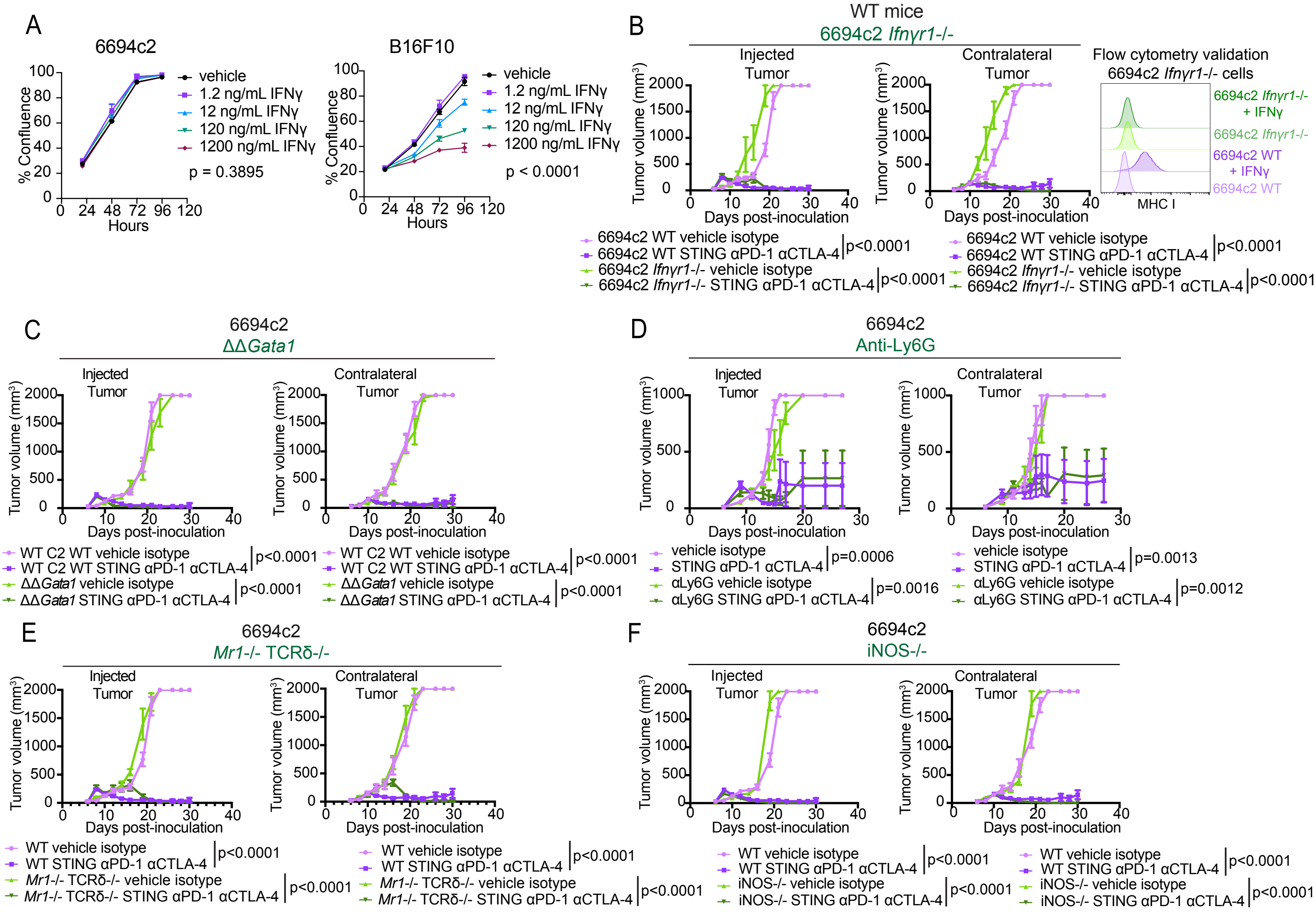

Figure S4. Tumor cell-expressed IFNγR, eosinophils, neutrophils, MAIT cells, γδ T cells, and inducible nitric oxide synthase are not required for tumor clearance in vivo, related to Figure 2.
(A) Confluence of 6694c2 (left) and B16F10 (right) cells when cultured with varying concentrations of IFNγ for 5 days. Tumor confluence was measured every 24 hours with a Celigo imaging cytometer. N=4 replicates. P values compare AUC of vehicle- and 1200 ng/mL IFNγ-treated groups via T-test (error bars report SEM). (B) Tumor growth curves of 6694c2 *Ifnγr1-/-* tumor cells in vivo. WT mice were inoculated with bilateral 6694c2 *Ifnγr1-/-* (green) or 6694c2 WT (purple) tumor cells. Validation of 6694c2 *Ifnγr1-/-* cells shown on the right. 6694c2 WT (purple) and 6694c2 *Ifnγr1-/-* (green) cells were treated with or without 20 ng/mL IFNγ for 24 hours. MHC-I levels were analyzed via flow cytometry. (C) ΔΔ*Gata1*, (D) WT mice treated with anti-Ly6G, (E) *Mr1-/-* TCRδ-/- and (F) iNOS-/- mice inoculated with 6694c2 tumors (green) compared to WT controls (purple). Seven days after inoculation, mice were intratumorally injected with STING agonist or vehicle and intraperitoneally injected with anti-PD-1 and anti-CTLA-4. Tumor growth was measured every 2-3 days. N=5. (B) 3/5 6694c2 WT-bearing and 4/5 6694c2 *Ifnγr1-/-*-bearing mice cured. (C) 3/5 WT and 3/5 ΔΔ*Gata1* mice cured. (D) Mice received 150 µg anti-Ly6G antibody starting 7 days prior to tumor inoculation and continuing every 2-3 days thereafter. 1/5 control and 0/4 anti-Ly6G-treated mice cured. (E) 4/5 *Mr1-/-* TCRδ-/- and 3/5 WT mice cured. (F) 3/5 WT mice and 4/5 iNOS-/- mice cured. N=5. Tumor growth curve significance was determined by calculating AUC in GraphPad Prism and conducting T-tests of areas (error bars report SEM).

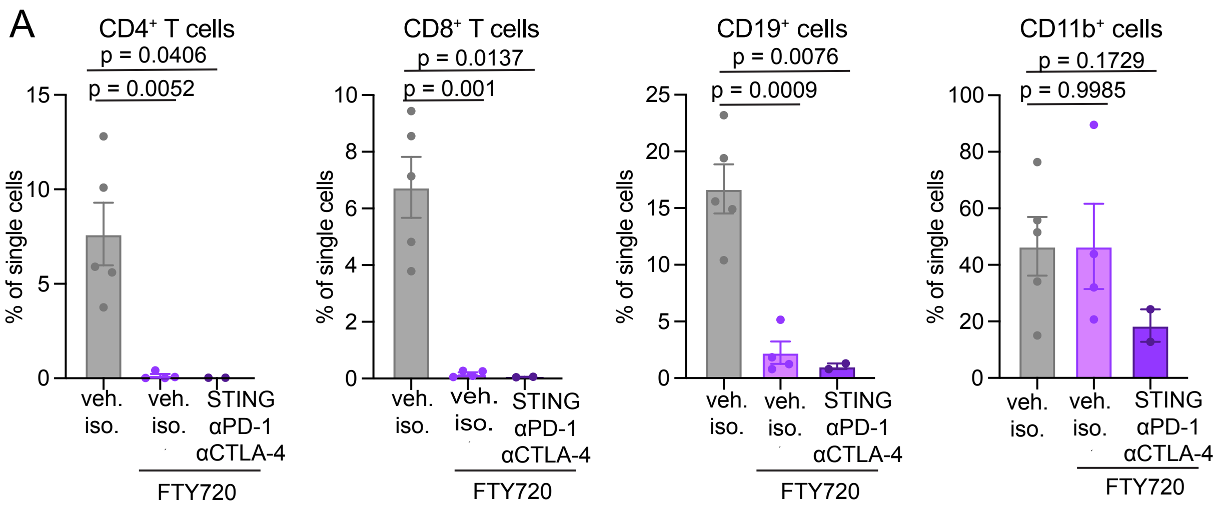

Figure S5. FTY720 prevents lymphocyte egress from lymph nodes, related to Figure 3. WT mice were inoculated with 6694c2 tumors, and 7 days post-inoculation, were treated with triple combination or control therapy. Also on day 7, daily oral gavage with FTY720 or water commenced. Mice were bled at endpoint prior to euthanasia. Blood was stained for flow cytometry analysis. (A) Bar graphs of $CD4^+$ T cells, $CD8^+$ T cells, $CD19^+$ cells, and $CD11b^+$ cells in the blood of mice treated with and without FTY720. N=5 vehicle, N=4 FTY720, N=2 STING αPD-1 αCTLA-4 FTY720 bled.

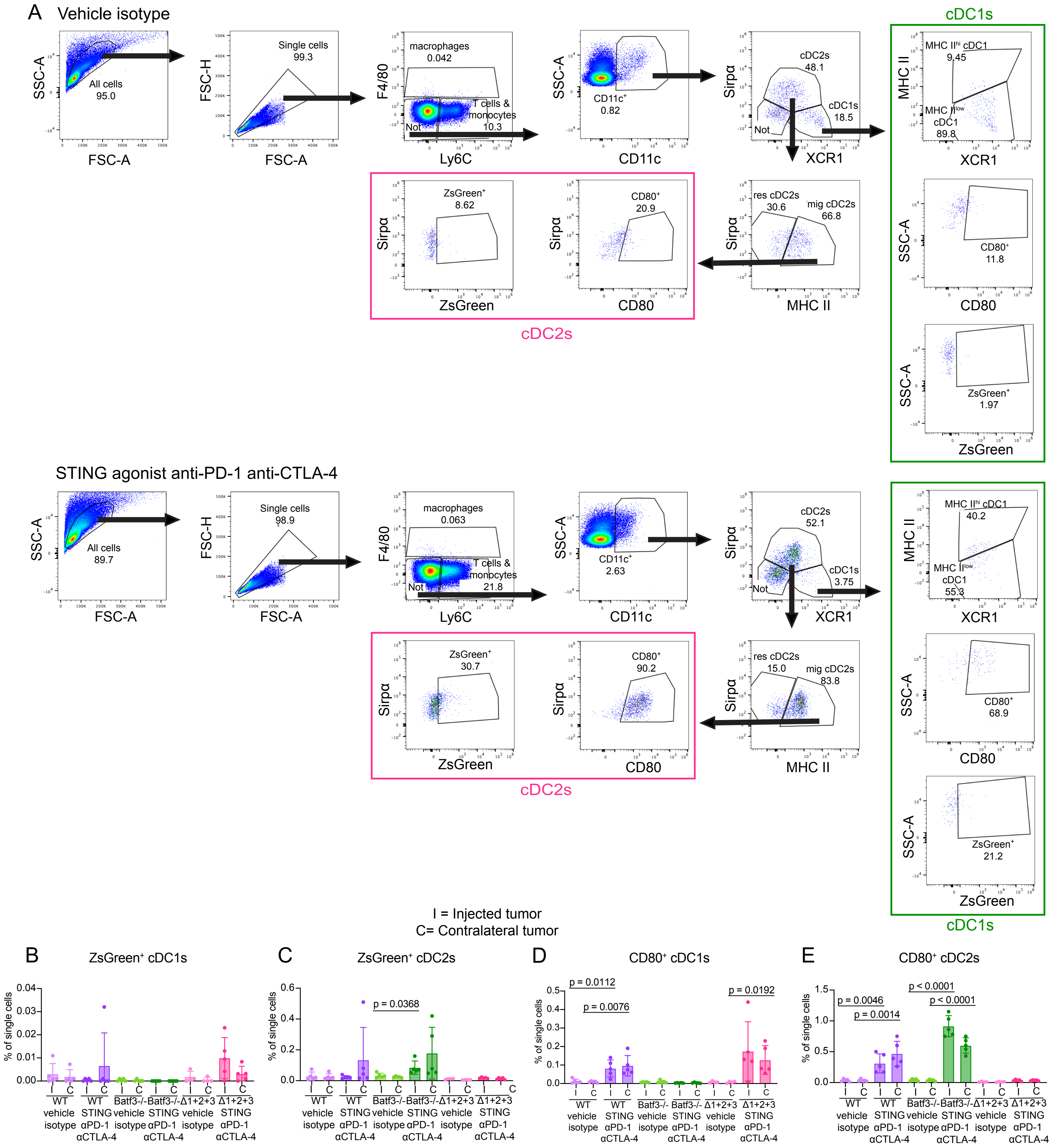

Figure S6. Dendritic cell population gating and verification, related to Figure 3. Mice were inoculated with 6694c2 ZsGreen tumors, and 7 days post-inoculation, they were treated with STING agonist anti-PD-1 and anti-CTLA-4 or vehicle isotype therapy. 48 hours later, tumor-draining lymph nodes were harvested, digested, and analyzed via flow cytometry. (A) (top) Representative flow plots from tumor-draining lymph nodes of vehicle isotype-treated mice. (bottom) Representative flow plots from tumor-draining lymph nodes of STING agonist anti-PD-1 and anti-CTLA-4-treated mice. (B-E) Bar graphs showing the percent of (B) ZsGreen^{+} cDC1s (C) ZsGreen^{+} cDC2s, (D) CD80^{+} cDC1s, (E) CD80^{+} cDC2s, out of single cells from tumor-draining lymph nodes of WT, *Batf3-/-*, and Δ1+2+3 mice treated with STING agonist anti-PD-1 and anti-CTLA-4 or vehicle and isotypes. I = injected side (lymph node draining the tumor that received STING agonist treatment), C = contralateral side (contralateral lymph node). N=5. Statistical significance was determined by conducting T-tests (error bars report SD).

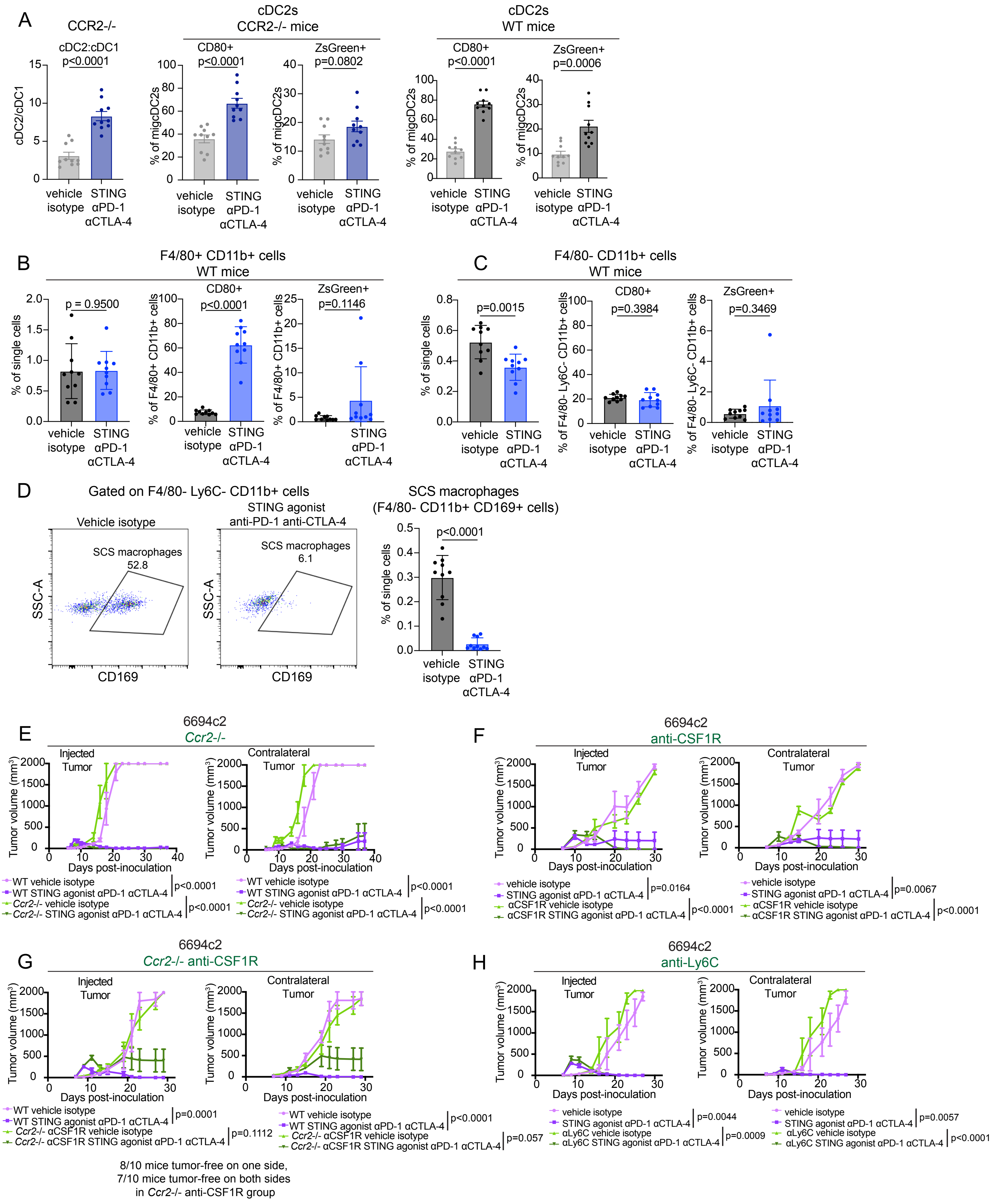
A
CCR2-/-
cDC2:cDC1
p<0.0001
cDC2/cDC1
vehicle isotype
STING αPD-1 αCTLA-4
cDC2s
CCR2-/- mice
CD80+
p<0.0001
% of migcDC2s
ZsGreen+
p=0.0802
cDC2s
WT mice
CD80+
p<0.0001
ZsGreen+
p=0.0006
B
F4/80+ CD11b+ cells
WT mice
p = 0.9500
% of single cells
CD80+
p<0.0001
% of F4/80+ CD11b+ cells
ZsGreen+
p=0.1146
C
F4/80- CD11b+ cells
WT mice
p=0.0015
CD80+
p=0.3984
% of F4/80- Ly6C- CD11b+ cells
ZsGreen+
p=0.3469
D
Gated on F4/80- Ly6C- CD11b+ cells
Vehicle isotype
SCS macrophages
52.8
STING agonist
anti-PD-1 anti-CTLA-4
SCS macrophages
6.1
SSC-A
CD169
SCS macrophages
(F4/80- CD11b+ CD169+ cells)
p<0.0001
E
6694c2
Ccr2-/-
Injected Tumor
Contralateral Tumor
Tumor volume (mm³)
Days post-inoculation
WT vehicle isotype
WT STING agonist αPD-1 αCTLA-4
Ccr2-/- vehicle isotype
Ccr2-/- STING agonist αPD-1 αCTLA-4
p<0.0001
F
6694c2
anti-CSF1R
vehicle isotype
STING agonist αPD-1 αCTLA-4
αCSF1R vehicle isotype
αCSF1R STING agonist αPD-1 αCTLA-4
p=0.0164
p<0.0001
p=0.0067
G
6694c2
Ccr2-/- anti-CSF1R
WT vehicle isotype
WT STING agonist αPD-1 αCTLA-4
Ccr2-/- αCSF1R vehicle isotype
Ccr2-/- αCSF1R STING agonist αPD-1 αCTLA-4
p=0.0001
p=0.1112
p<0.0001
p=0.057
8/10 mice tumor-free on one side,
7/10 mice tumor-free on both sides
in Ccr2-/- anti-CSF1R group
H
6694c2
anti-Ly6C
vehicle isotype
STING agonist αPD-1 αCTLA-4
αLy6C vehicle isotype
αLy6C STING agonist αPD-1 αCTLA-4
p=0.0044
p=0.0009
p=0.0057
p<0.0001

Figure S7. cDC2 populations identified are not macrophages/monocytes, and macrophages are not required for triple combination therapy efficacy, related to Figure 3. (A) Bar graphs showing cDC2:cDC1 ratio, fraction of $CD80^{+}$ cDC2s, and fraction of $ZsGreen^{+}$ cDC2s in CCR2-/- and WT mice, respectively. (B) Bar graphs showing frequency of $F4/80^{+}$ $CD11b^{+}$ cells out of single cells, and fractions of $CD80^{+}$ $F4/80^{+}$ $CD11b^{+}$ cells and $ZsGreen^{+}$ $F4/80^{+}$ $CD11b^{+}$ cells in vehicle and triple combination therapy-treated mice. (C) Bar graphs showing frequency of $F4/80^{-}CD11b^{+}$ cells out of single cells and fractions of $CD80^{+}$ $F4/80^{-}$ $CD11b^{+}$ cells and $ZsGreen^{+}$ $F4/80^{-}$ $CD11b^{+}$ cells in vehicle and triple combination therapy-treated mice. (D) Representative flow plots and bar graph showing frequencies of subcapsular sinus (SCS) macrophages in tumor-draining lymph nodes of mice treated with control or triple combination therapy. (A-D) In all panels, tumor-draining lymph nodes were harvested two days post-STING agonist anti-PD-1 anti-CTLA-4 treatment. N=10. Statistical significance was determined by conducting T-tests (error bars report SD). (E, G) *Ccr2*-/- and (F, H) WT mice (green) were inoculated with bilateral 6694c2 subcutaneous tumors. (F-H) Starting seven days prior to inoculation, mice were treated with (F-G) anti-CSF1R or (H) anti-Ly6C antibody (150 µg, every 2-3 days). (E) WT 4/5 and *Ccr2*-/- 2/4 cured. (F) 4/5 control and 5/5 anti-CSF1R-treated mice cured. (G) 5/5 WT and 7/10 *Ccr2*-/- anti-CSF1R mice cured. (H) 4/4 control and 4/4 anti-Ly6C-treated mice cured. Seven days post-6694c2 tumor inoculation, one tumor was injected with STING agonist or vehicle, and mice were treated systemically with anti-PD-1 and anti-CTLA-4 (checkpoint blockade repeated weekly). Tumor growth was measured, and mice were euthanized when they reached the humane endpoint of >2000 mm^3. Tumor growth curve significance was determined by calculating AUC in GraphPad Prism and conducting T-tests of areas (error bars report SEM).

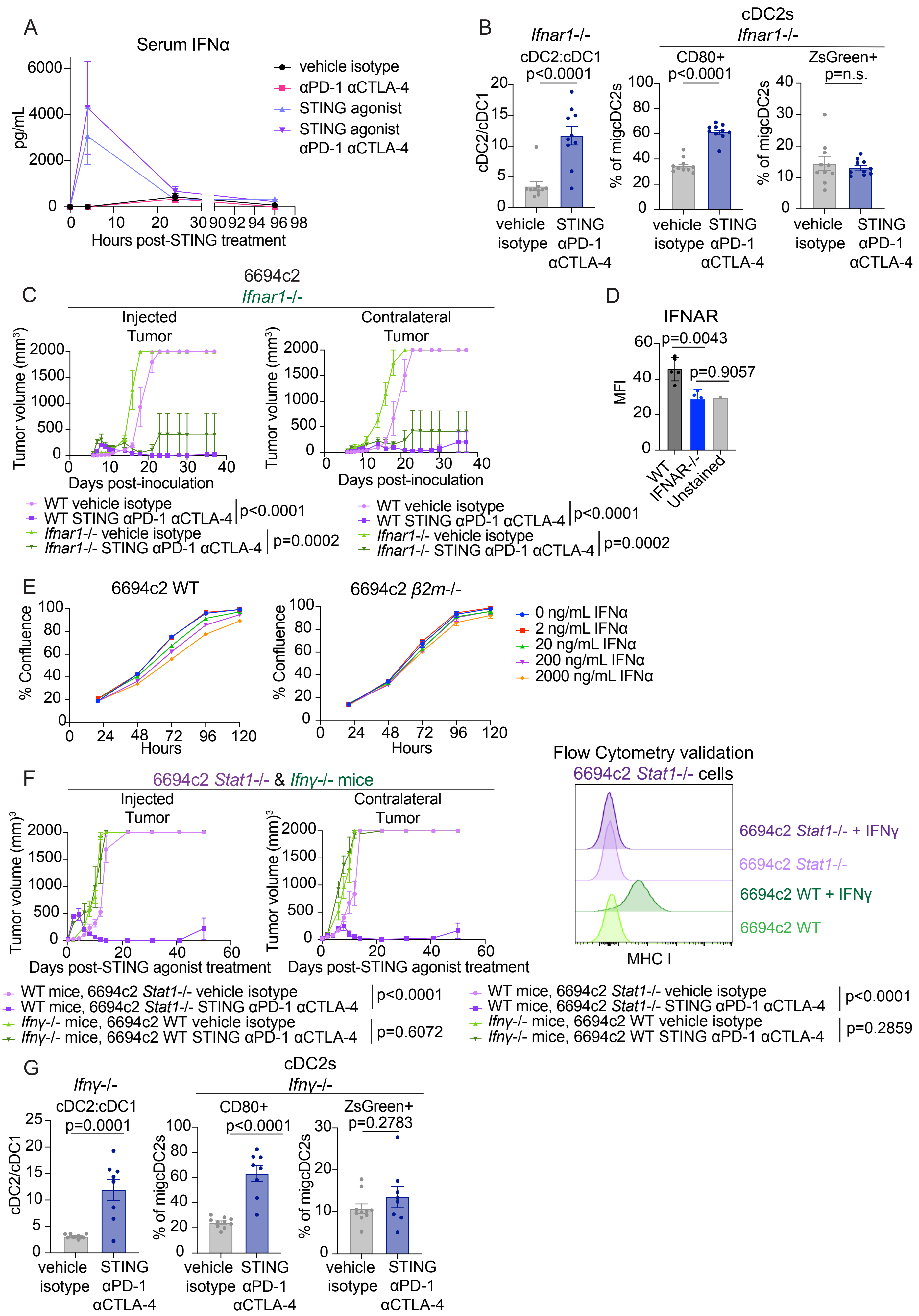
A
Serum IFNα
pg/mL
Hours post-STING treatment
vehicle isotype
αPD-1 αCTLA-4
STING agonist
STING agonist αPD-1 αCTLA-4
B
Ifnar1-/-
cDC2:cDC1
p<0.0001
cDC2/cDC1
cDC2s
Ifnar1-/-
CD80+
p<0.0001
% of migcDC2s
ZsGreen+
p=n.s.
vehicle isotype
STING αPD-1 αCTLA-4
C
6694c2
Ifnar1-/-
Injected Tumor
Contralateral Tumor
Tumor volume (mm³)
Days post-inoculation
WT vehicle isotype
WT STING αPD-1 αCTLA-4
p<0.0001
Ifnar1-/- vehicle isotype
Ifnar1-/- STING αPD-1 αCTLA-4
p=0.0002
D
IFNAR
MFI
p=0.0043
p=0.9057
WT
IFNAR-/-
Unstained
E
6694c2 WT
6694c2 β2m-/-
% Confluence
Hours
0 ng/mL IFNα
2 ng/mL IFNα
20 ng/mL IFNα
200 ng/mL IFNα
2000 ng/mL IFNα
F
6694c2 Stat1-/- & Ifnγ-/- mice
Injected Tumor
Contralateral Tumor
Tumor volume (mm)³
Days post-STING agonist treatment
WT mice, 6694c2 Stat1-/- vehicle isotype
WT mice, 6694c2 Stat1-/- STING αPD-1 αCTLA-4
p<0.0001
Ifnγ-/- mice, 6694c2 WT vehicle isotype
Ifnγ-/- mice, 6694c2 WT STING αPD-1 αCTLA-4
p=0.6072
p=0.2859
Flow Cytometry validation
6694c2 Stat1-/- cells
6694c2 Stat1-/- + IFNγ
6694c2 Stat1-/-
6694c2 WT + IFNγ
6694c2 WT
MHC I
G
Ifnγ-/-
cDC2:cDC1
p=0.0001
cDC2/cDC1
cDC2s
Ifnγ-/-
CD80+
p<0.0001
% of migcDC2s
ZsGreen+
p=0.2783
vehicle isotype
STING αPD-1 αCTLA-4

Figure S8. Type I IFN is not required for triple combination therapy efficacy, related to Figure 3.

(A) Graph showing 6694c2-bearing mouse serum concentrations of IFNα after STING agonist and triple combination therapy. At intervals, mice were bled, and serum was isolated. An IFNα ELISA was conducted to determine the in vivo kinetics of type I IFN production post-treatment. N=5. (B) Bar graph showing the cDC2:cDC1 ratio and frequency of $CD80^+$ and $ZsGreen^+$ cDC2s in the 6694c2 tumor-draining lymph nodes of *Ifnar1*-/- (N=5 mice) and (G) *Ifnγ*-/- (N=4 triple combination-treated, N=5 vehicle isotypes-treated mice) mice 48 hours post-STING agonist anti-PD-1 and anti-CTLA-4 or control therapy. Tumor-draining lymph nodes were analyzed via flow cytometry. (C) Tumor growth curve in *Ifnar1*-/- mice. *Ifnar1*-/- (green) or WT (purple) mice were inoculated with 6694c2 tumors, and 7 days-post inoculation were treated with STING agonist anti-PD-1 and anti-CTLA-4. Tumor growth was measured every 2-3 days. Checkpoint blockade was administered weekly. N=5, except *Ifnar1*-/- vehicle isotype, where N=4. 4/5 WT mice and 3/5 *Ifnar1*-/- mice were cured. (D) Bar graph showing the MFI of IFNAR staining on immune cells taken from the WT and Ifnar1-/- mice used in panel C. At the time of harvest/sacrifice, mice were bled, and their cells were stained for IFNAR. (E) 6694c2 and 6694c2 *β2m*-/- in vitro confluence upon treatment with IFNα for 5 days. Measurements were taken with a Celigo imaging cytometer. (F) Tumor growth curve showing in vivo tumor growth of 6694c2 *Stat1*-/- tumors (purple) in WT mice compared to 6694c2 tumor cells in *Ifnγ*-/- mice (green). Mice were inoculated with tumors and treated with STING agonist anti-PD-1 anti-CTLA-4 or control therapy seven days later. Checkpoint blockade was administered weekly. N=5, except *Ifnγ*-/- vehicle isotype, where N=4. 3/5 6694c2 *Stat1*-/- tumors cured (0/5 *Ifnγ*-/- mice cured). Flow cytometry validation of 6694c2 *Stat1*-/- cells shown on right; 6694c2 WT (green) and 6694c2 *Stat1*-/-(purple) cells were treated with or without 20 ng/mL IFNγ for 24 hours. MHC-I expression was analyzed via flow cytometry. Tumor growth curve significance was determined by calculating AUC in GraphPad Prism and conducting T-tests of areas (error bars report SEM). Other p values were calculated by performing T-tests (error bars report SD).

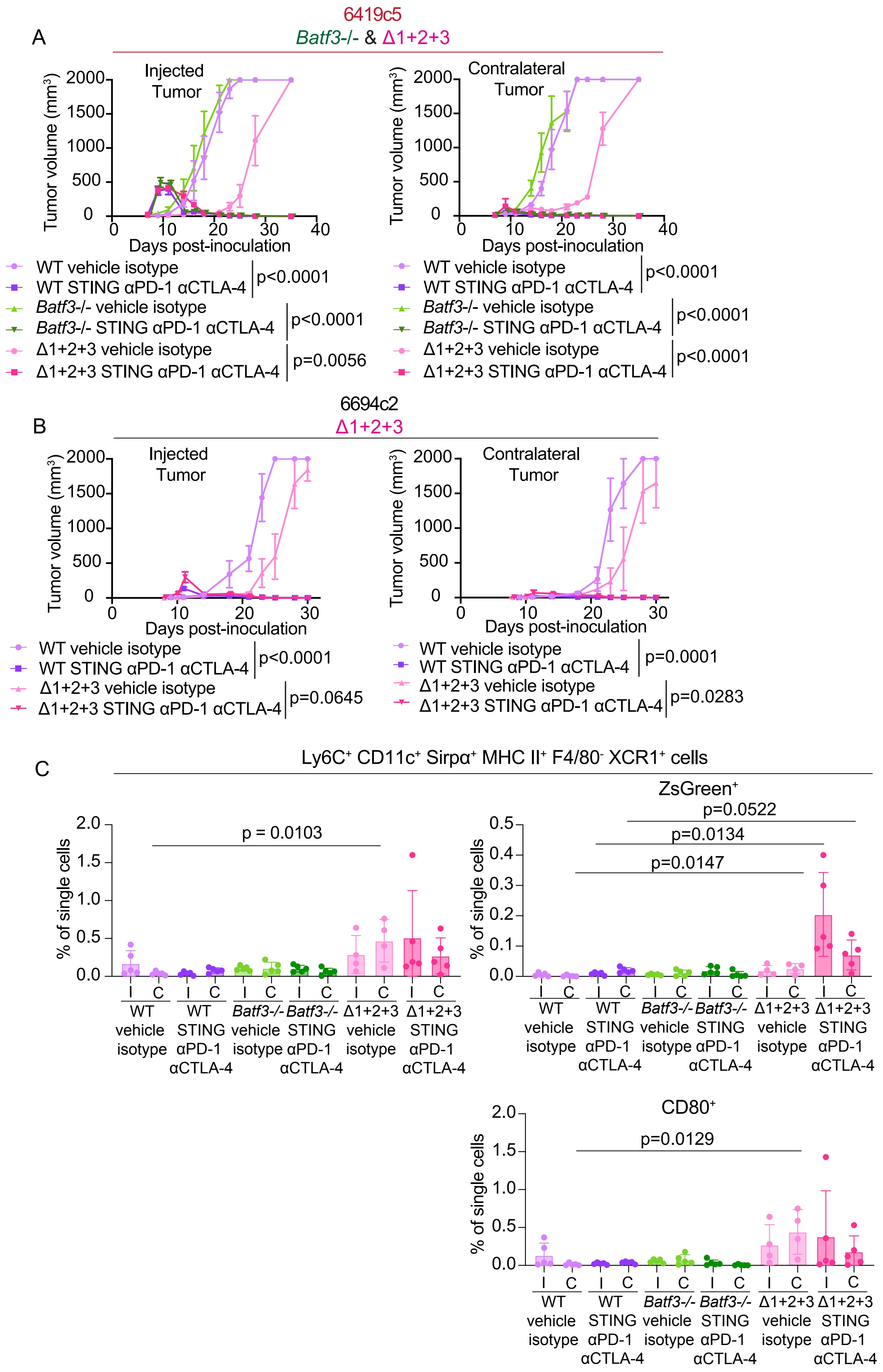

A
6419c5
Batf3-/- & Δ1+2+3
Injected Tumor
Contralateral Tumor
Tumor volume (mm³)
Days post-inoculation
WT vehicle isotype
WT STING αPD-1 αCTLA-4
p<0.0001
Batf3-/- vehicle isotype
Batf3-/- STING αPD-1 αCTLA-4
p<0.0001
Δ1+2+3 vehicle isotype
Δ1+2+3 STING αPD-1 αCTLA-4
p=0.0056
WT vehicle isotype
WT STING αPD-1 αCTLA-4
p<0.0001
Batf3-/- vehicle isotype
Batf3-/- STING αPD-1 αCTLA-4
p<0.0001
Δ1+2+3 vehicle isotype
Δ1+2+3 STING αPD-1 αCTLA-4
p<0.0001
B
6694c2
Δ1+2+3
Injected Tumor
Contralateral Tumor
Tumor volume (mm³)
Days post-inoculation
WT vehicle isotype
WT STING αPD-1 αCTLA-4
p<0.0001
Δ1+2+3 vehicle isotype
Δ1+2+3 STING αPD-1 αCTLA-4
p=0.0645
WT vehicle isotype
WT STING αPD-1 αCTLA-4
p=0.0001
Δ1+2+3 vehicle isotype
Δ1+2+3 STING αPD-1 αCTLA-4
p=0.0283
C
Ly6C⁺ CD11c⁺ Sirpα⁺ MHC II⁺ F4/80⁻ XCR1⁺ cells
% of single cells
p = 0.0103
ZsGreen⁺
p=0.0522
p=0.0134
p=0.0147
CD80⁺
p=0.0129
I C
WT vehicle isotype
WT STING αPD-1 αCTLA-4
Batf3-/- vehicle isotype
Batf3-/- STING αPD-1 αCTLA-4
Δ1+2+3 vehicle isotype
Δ1+2+3 STING αPD-1 αCTLA-4

Figure S9. cDC2s are not required for rejection of subcutaneous pancreatic tumors, and Ly6C$^+$ cells appear in the setting of cDC2 loss, related to Figure 5.
(A-B) Tumor growth curves of mice inoculated with (A) 6419c5 or (B) 6694c2 subcutaneous tumors. (A) WT (purple), *Batf3*-/- (green), and Δ1+2+3 (pink) mice were inoculated with 6419c5 tumors. Seven days later, one tumor was injected with STING agonist or vehicle. Mice received dual checkpoint blockade or isotypes weekly. 5/5 WT, 5/5 *Batf3*-/- and 5/5 Δ1+2+3 mice were cured. (B) Same as A, except mice received 6694c2 tumors, and only WT and Δ1+2+3 mice were compared. 4/5 WT and 4/5 Δ1+2+3 mice were cured. (C) Bar graphs showing the percent of Ly6C$^+$ CD11c$^+$ Sirpα$^+$ MHC-II$^+$ F4/80$^-$ XCR1$^-$ cells in tumor-draining lymph nodes out of total single cells and fractions of CD80$^+$ and ZsGreen$^+$ Ly6C$^+$ CD11c$^+$ Sirpα$^+$ MHC-II$^+$ F4/80$^-$ XCR1$^-$ cells. I = injected side (lymph node draining the tumor that received STING agonist treatment), C = contralateral side (contralateral lymph node). WT, *Batf3*-/-, and Δ1+2+3 mice bearing 6694c2 ZsGreen tumors were treated with STING agonist anti-PD-1 and anti-CTLA-4 or vehicle and isotypes seven days post-inoculation. Two days later, tumor-draining lymph nodes were harvested and digested for flow cytometry analysis. N=5 mice in all groups except for N=4 in both groups with Δ1+2+3 mice. Significance of tumor growth curves was determined by calculating AUC in GraphPad Prism and conducting T-tests of areas (error bars report SEM). Other statistical significance was determined by conducting T-tests (error bars report SD).

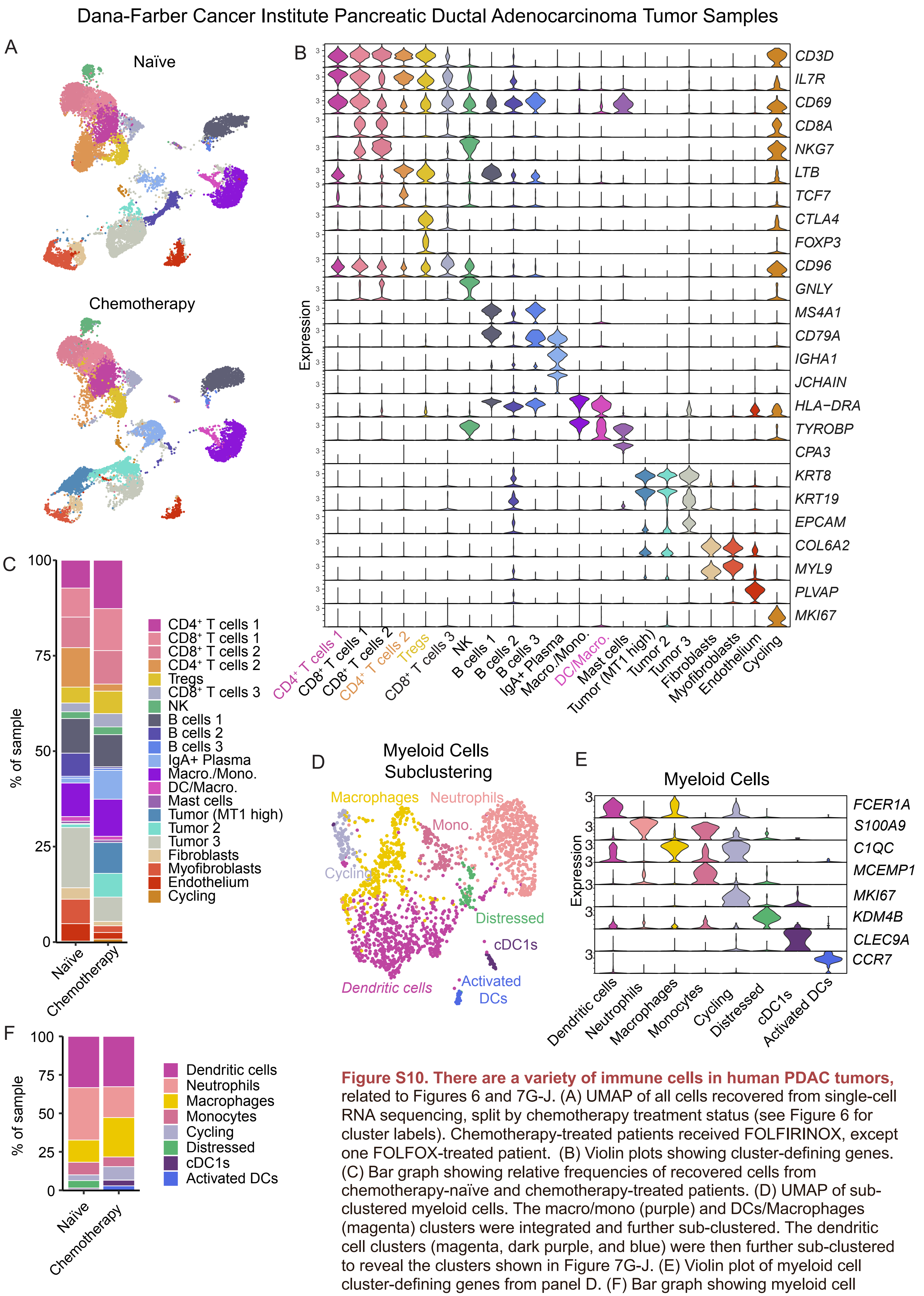

Figure S10. There are a variety of immune cells in human PDAC tumors, related to Figures 6 and 7G-J. (A) UMAP of all cells recovered from single-cell RNA sequencing, split by chemotherapy treatment status (see Figure 6 for cluster labels). Chemotherapy-treated patients received FOLFIRINOX, except one FOLFOX-treated patient. (B) Violin plots showing cluster-defining genes. (C) Bar graph showing relative frequencies of recovered cells from chemotherapy-naïve and chemotherapy-treated patients. (D) UMAP of sub-clustered myeloid cells. The macro/mono (purple) and DCs/Macrophages (magenta) clusters were integrated and further sub-clustered. The dendritic cell clusters (magenta, dark purple, and blue) were then further sub-clustered to reveal the clusters shown in Figure 7G-J. (E) Violin plot of myeloid cell cluster-defining genes from panel D. (F) Bar graph showing myeloid cell relative frequencies.

Data reanalyzed from Loveless, et al., *Clin. Cancer. Res.*, 2025.

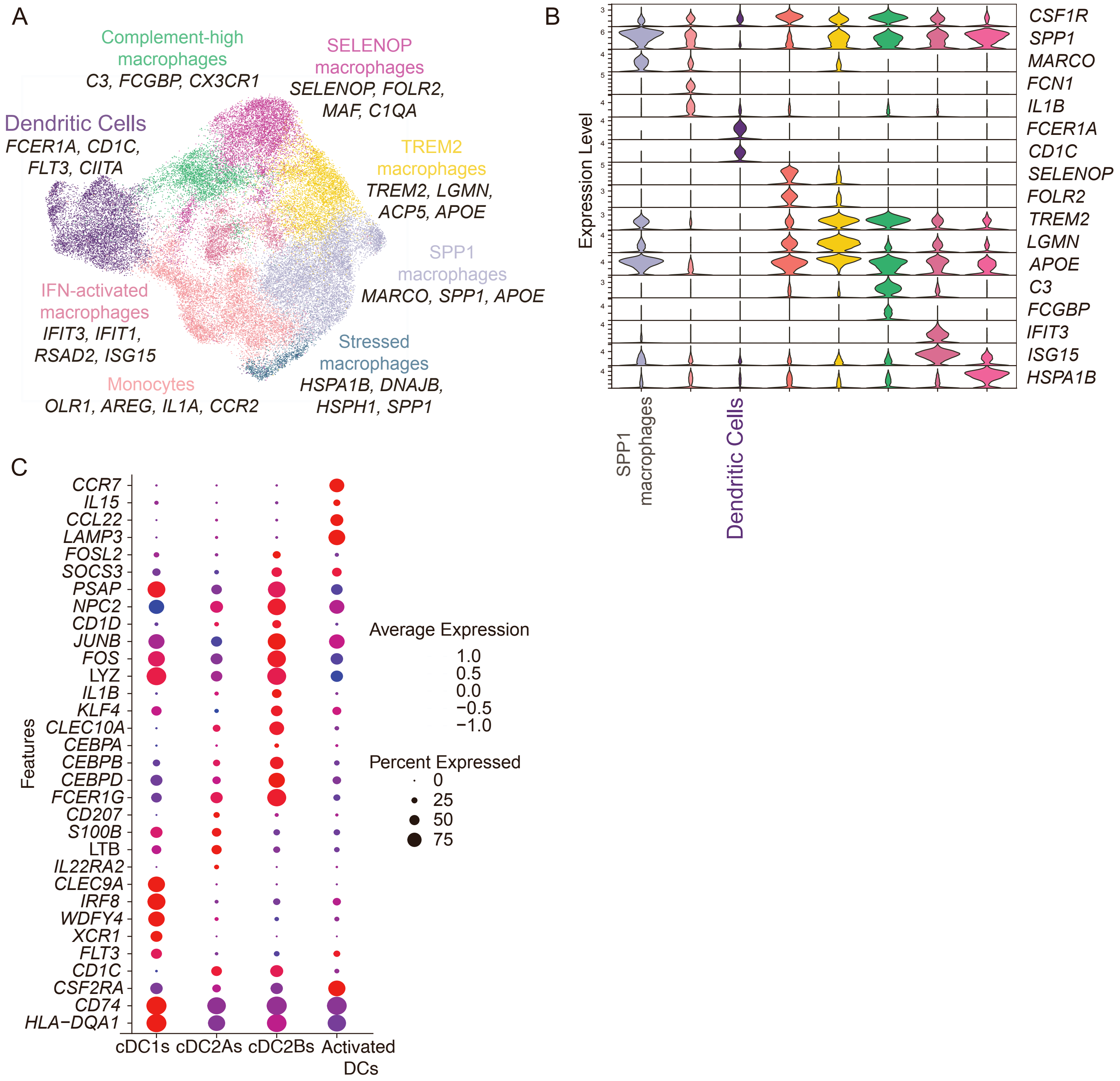

Figure S11. Myeloid cells are present in published human PDAC tumor datasets, related to Figure 7A-C. All data reanalyzed from Loveless et al., *Clin. Cancer Res.*, 2025. (A) UMAP of myeloid cells. (B) Violin plots of myeloid cell cluster-defining genes. (C) Dot plot of differentially expressed genes between the dendritic cell subsets shown in Figure 7A-C.

Data reanalyzed from Hwang, Jagadeesh, Guo, & Hoffman et al., *Nat. Genetics.*, 2022.

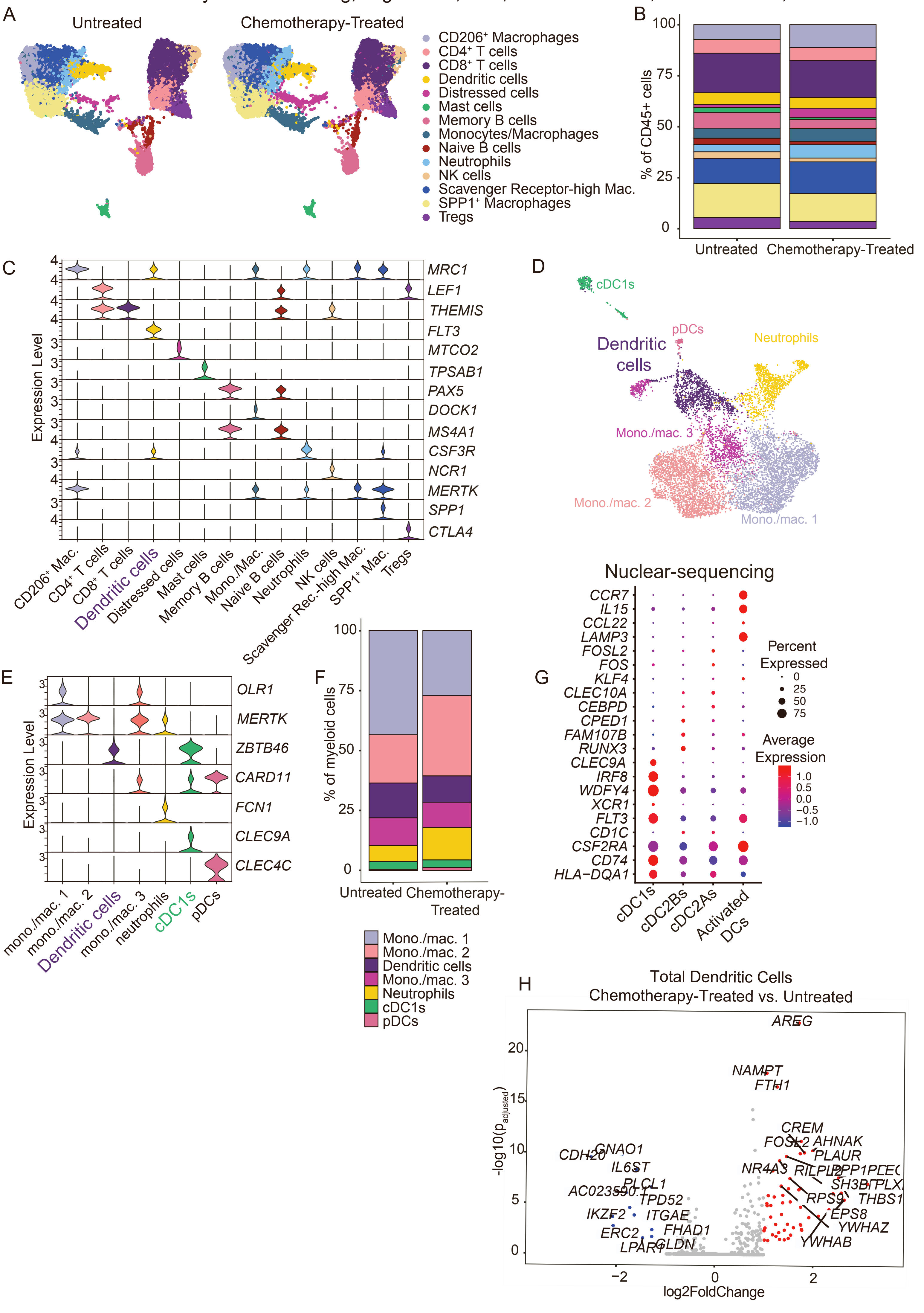

Figure S12. There are a variety of immune cells in PDAC tumors, and their frequencies do not significantly change after chemotherapy treatment, related to Figure 7D-F. All data reanalyzed from Hwang, Jagadeesh, Guo, & Hoffman et al., *Nat. Genetics*, 2022. (A) UMAP of all cells recovered from PDAC tumors, split by chemotherapy treatment status. (B) Relative frequencies of recovered cells, split by treatment status. (C) Violin plot of cluster-defining genes. (D) UMAP of myeloid cells. Dendritic cell clustering shown in Figure 7 done by sub-clustering the dendritic cell cluster and removing contaminants. (E) Violin plot of myeloid cell cluster-defining genes. (F) Relative frequencies of myeloid cells, split by treatment status. (G) Dot plot of dendritic cell cluster-defining genes. (H) Volcano plot of differentially expressed genes between dendritic cells that arose from chemotherapy-naïve and -treated tumors.

Figure	Panel	Group	Cure rate
1	B	STING αPD-1 αCTLA-4	5/5
	C	STING αPD-1 αCTLA-4	5/5
		αNK1.1 STING αPD-1 αCTLA-4	4/5
	D	WT STING αPD-1 αCTLA-4	3/10
		β2m-/- STING αPD-1 αCTLA-4	3/5
	E	STING αPD-1 αCTLA-4	5/5
		αCD8 STING αPD-1 αCTLA-4	3/5
	F	STING αPD-1 αCTLA-4	4/5
		αCD8 STING αPD-1 αCTLA-4	3/5
	G	STING αPD-1 αCTLA-4	5/5
		αCD4 STING αPD-1 αCTLA-4	0/5
	H	WT STING αPD-1 αCTLA-4	5/5
		I-Ab-/- STING αPD-1 αCTLA-4	0/5
	I	STING αPD-1 αCTLA-4	4/5
		αCD4 STING αPD-1 αCTLA-4	0/5
2	F	WT STING αPD-1 αCTLA-4	4/5
		Ifnγ-/- STING αPD-1 αCTLA-4	0/5
	G	STING αPD-1 αCTLA-4	5/5
		αIFNγ STING αPD-1 αCTLA-4	0/5
	H	WT STING αPD-1 αCTLA-4	5/5
		Ifnγ-/- STING αPD-1 αCTLA-4	0/5
	I	WT STING αPD-1 αCTLA-4	4/5
		Tbx21-/- STING αPD-1 αCTLA-4	0/5
3	A	STING αPD-1 αCTLA-4	3/5
		FTY720 STING αPD-1 αCTLA-4	0/5
5	F	WT STING αPD-1 αCTLA-4	4/5
		Batf3-/- STING αPD-1 αCTLA-4	3/4
	G	$CD11c^{cre-}$ MHC $II^{fl/fl}$ STING αPD-1 αCTLA-4	5/5
		$CD11c^{cre+}$ MHC $II^{fl/fl}$ STING αPD-1 αCTLA-4	0/5
	H	$LysM^{cre-}$ MHC $II^{fl/fl}$ STING αPD-1 αCTLA-4	4/5
		$LysM^{cre+}$ MHC $II^{fl/fl}$ STING αPD-1 αCTLA-4	4/5

Table S1. In vivo tumor growth curve cure rates, related to Figures 1, 2, 3, and 5. Cure rates of in vivo triple combination therapy-treated groups.

PANFR number	Neoadjuvant radiation?	treatment
PAN03237	no	gemcitabine/n(ab)paclitaxel
PAN03243	no	FOLFIRINOX
PAN03251	no	FOLFIRINOX
PAN03258	no	FOLFIRINOX
PAN03288	no	gemcitabine/n(ab)paclitaxel
PAN03344	no	FOLFIRINOX
PAN03354	no	FOLFIRINOX
PAN03375	no	FOLFIRINOX
PAN03377	no	FOLFIRINOX
PAN03407	no	gemcitabine/n(ab)paclitaxel
PAN03797	no	FOLFIRINOX
PAN03800	no	FOLFIRINOX
PAN03803	no	FOLFIRINOX
PAN03846	no	FOLFIRINOX
PAN03279	no	gemcitabine/n(ab)paclitaxel
PAN03336	no	FOLFIRINOX
PAN03363	no	FOLFIRINOX
PAN03474	no	gemcitabine/n(ab)paclitaxel
PAN03867	no	FOLFIRINOX
PAN03804	no	gemcitabine/n(ab)paclitaxel
PAN04179	no	FOLFIRINOX
PAN03856	no	FOLFIRINOX
PAN04080	no	treatment naïve
PAN03962	no	FOLFOX
PAN04114	no	FOLFIRINOX
PAN03491	no	FOLFIRINOX
PAN04271	yes	FOLFIRINOX
PAN04050	no	treatment naïve
PAN04137	no	FOLFIRINOX
PAN90136 (new ID PAN04091)	no	treatment naïve
PAN04081	no	FOLFIRINOX
PAN04158	no	treatment naïve
PAN04164	no	treatment naïve
PAN04830	no	treatment naïve
PAN04832	no	treatment naïve
PAN05676	no	treatment naïve
PAN05713	no	treatment naïve
PAN05812	no	treatment naïve

stage at time of blood draw	PBMC analysis	tumor 10x analysis	gender
met	yes	no	F
met	yes	no	M
met	yes	no	F
met	yes	no	F
met	yes	no	M
met	yes	no	F
met	yes	no	F
met	yes	no	M
met	yes	no	M
local- borderline resect neoadjuvant	yes	no	M
local- borderline resect neoadjuvant	yes	no	F
local- borderline resect neoadjuvant	yes	no	F
met	yes	no	M
local - resectable adjuvant	yes	no	F
LA - neoadjuvant	yes	no	M
met	yes	no	M
met	yes	no	F
met	yes	no	M
met	yes	no	M
LA - unresectable pallative adjuvant chemo	yes	no	F
local - borderline resect neoadjuvant	no	yes	M
local - resectable neoadjuvant	no	yes	M
local borderline resect - neoadjuvant	no	yes	M
local borderline resect - neoadjuvant	no	yes	M
local - neoadjuvant	no	yes	M
local borderline resect - neoadjuvant	no	yes	M
Local borderline resect - neoadjuvant	no	yes	F
local - upfront resection	no	yes	F
local borderline resect - neoadjuvant	no	yes	F
local - upfront resection	no	yes	F
local - resectable neoadjuvant	no	yes	M
local - upfront resection	no	yes	F
local - upfront resection	no	yes	F
local - upfront resection	no	yes	F
local - upfront resection	no	yes	M
local - upfront resection	no	yes	F
local - upfront resection	no	yes	M
local - upfront resection	no	yes	F

race	age at diagnosis	Cancer Primary Site ICD-O-3	Liver Met at Diagnosis?	Diabetes Status
White	65	Head of pancreas	Yes	No
White	57	Body of pancreas	Yes	Yes
Black	64	Body of pancreas	Yes	No
White	61	Head of pancreas	Yes	Yes
White	62	Head of pancreas	Yes	No
White	80	Head of pancreas	Yes	No
White	72	Tail of pancreas	Yes	No
White	57	Pancreas NOS	Yes	Yes
White	41	Head of pancreas	Yes	No
White	71	Head of pancreas	No	No
White	67	Head of pancreas	No	No
White	68	Head of pancreas	No	
White	53	Overlapping lesion of pancreas	No	Yes
White	64	Tail of pancreas	No	No
White	72	Tail of pancreas	No	Yes
White	62	Body of pancreas	Yes	No
White	70	Body of pancreas	Yes	Yes
White	80	Tail of pancreas	Yes	No
White	67	Head of pancreas	No	No
White	72	Body of pancreas	No	Yes
White	64	Head of pancreas	No	Yes
White	52	Head of pancreas	No	No
White	65	Head of pancreas	No	Yes
White	79	Head of pancreas	No	Yes
White	71	Head of pancreas	No	Yes
White	75	Pancreas NOS	No	No
White	77	Head of pancreas	Yes	No
Unknown	63	Body of pancreas	No	No
White	64	Pancreas NOS	No	No
White	71	Tail of pancreas	No	No
White	78	Pancreas NOS	No	No
White	76	Body of pancreas	No	No
White	69	Head of pancreas	No	No
White	64	Body of pancreas	No	No
White	29	Head of pancreas	No	No
White	74	Head of pancreas	No	Yes
White	60	Body of pancreas	No	No
White	73	Body of pancreas	No	Yes

Table S2. Additional patient characteristics, related to Table 1. Demographic information of patient cohort, listed by patient.
