# Supplementary material for: Dendritic cell redundancy enables priming of anti-tumor CD4+ T cells in pancreatic cancer
Source: Cancer Cell. Author manuscript; Available in PMC 2026 Aug 3. (PMC13430408; doi:10.1016/j.ccell.2026.04.005)
Supplement: Supplemental data file [file NIHMS2192170-supplement-Supplemental_data_file.pdf]

Data S1. Raw western blot images and representative flow cytometry, related to STAR Methods.

A Raw blot image: anti-caspase 8

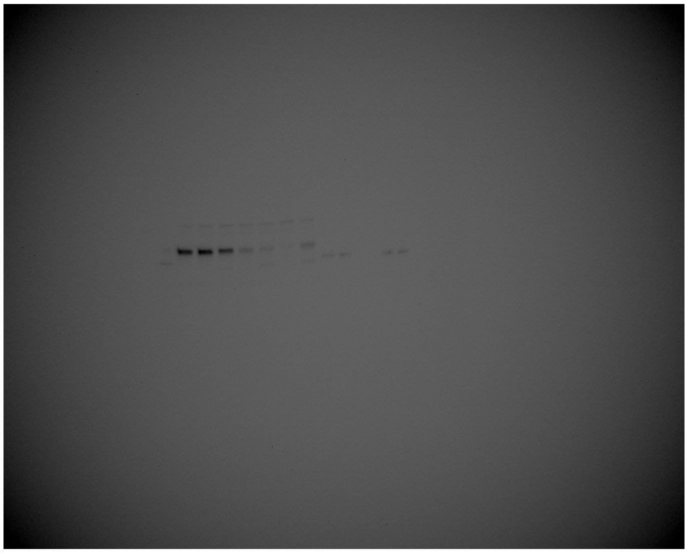

Raw blot image: anti-GAPDH

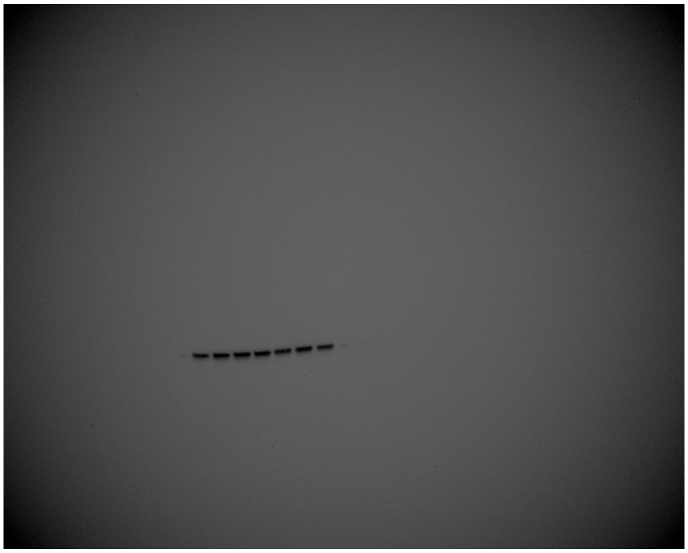

Raw, uncropped blot images. 6694c2 WT, B16 nectin-1, 6694c2 empty vector, and four 6694c2 lines transduced with caspase-8 single-guide RNAs were lysed with 0.5% NP-40, 50mM HEPES, 50mM NaCl, and protease and phosphatase inhibitor. A BCA assay was performed to normalize for protein amounts.

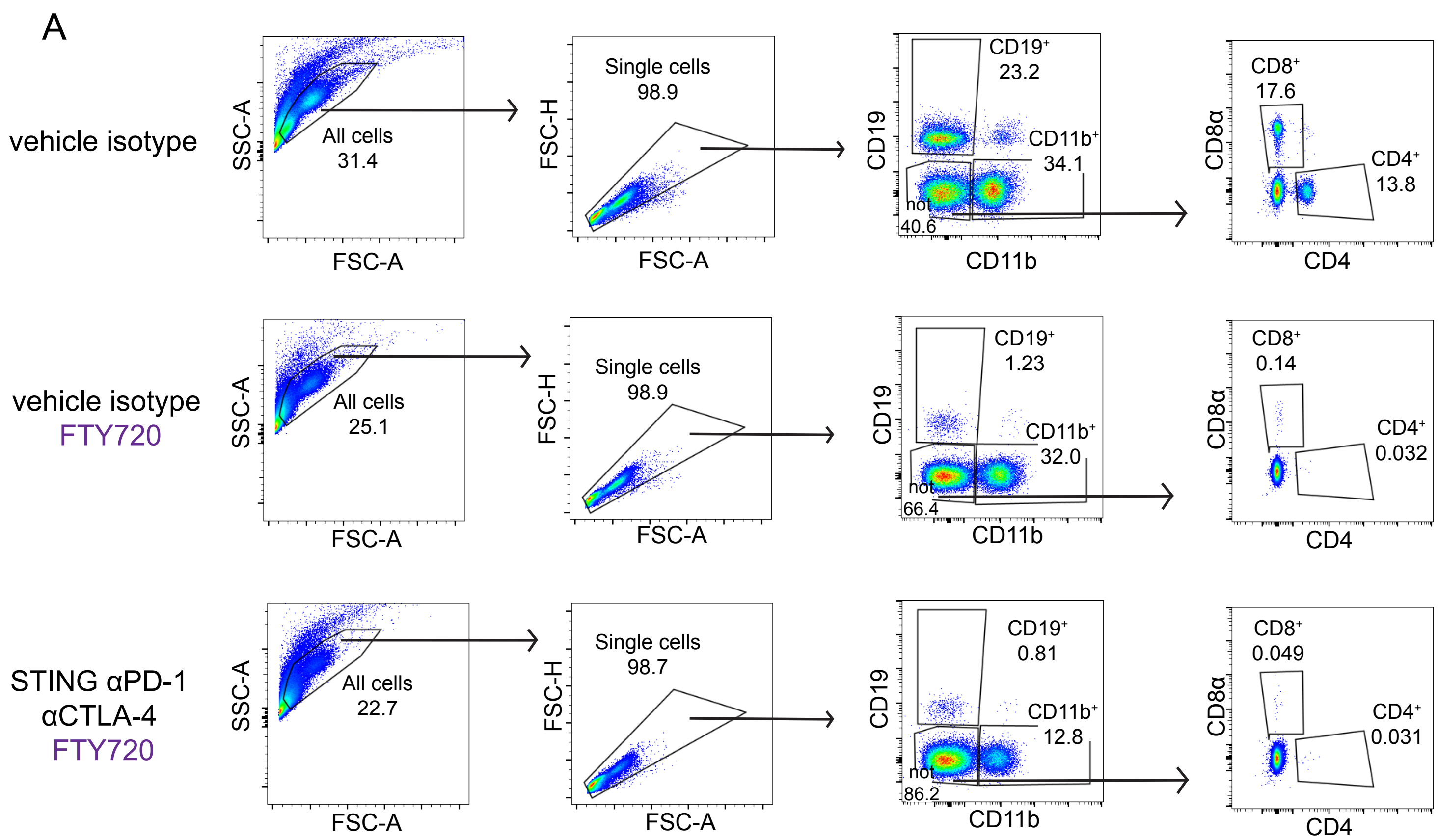

WT mice were inoculated with 6694c2 tumors, and 7 days post-inoculation, were treated with triple combination or control therapy. Also on day 7, daily oral gavage with FTY720 or water commenced. Mice were bled at endpoint prior to euthanasia. Blood was stained for flow cytometry analysis. Related to Figure 3 and Figure S5.

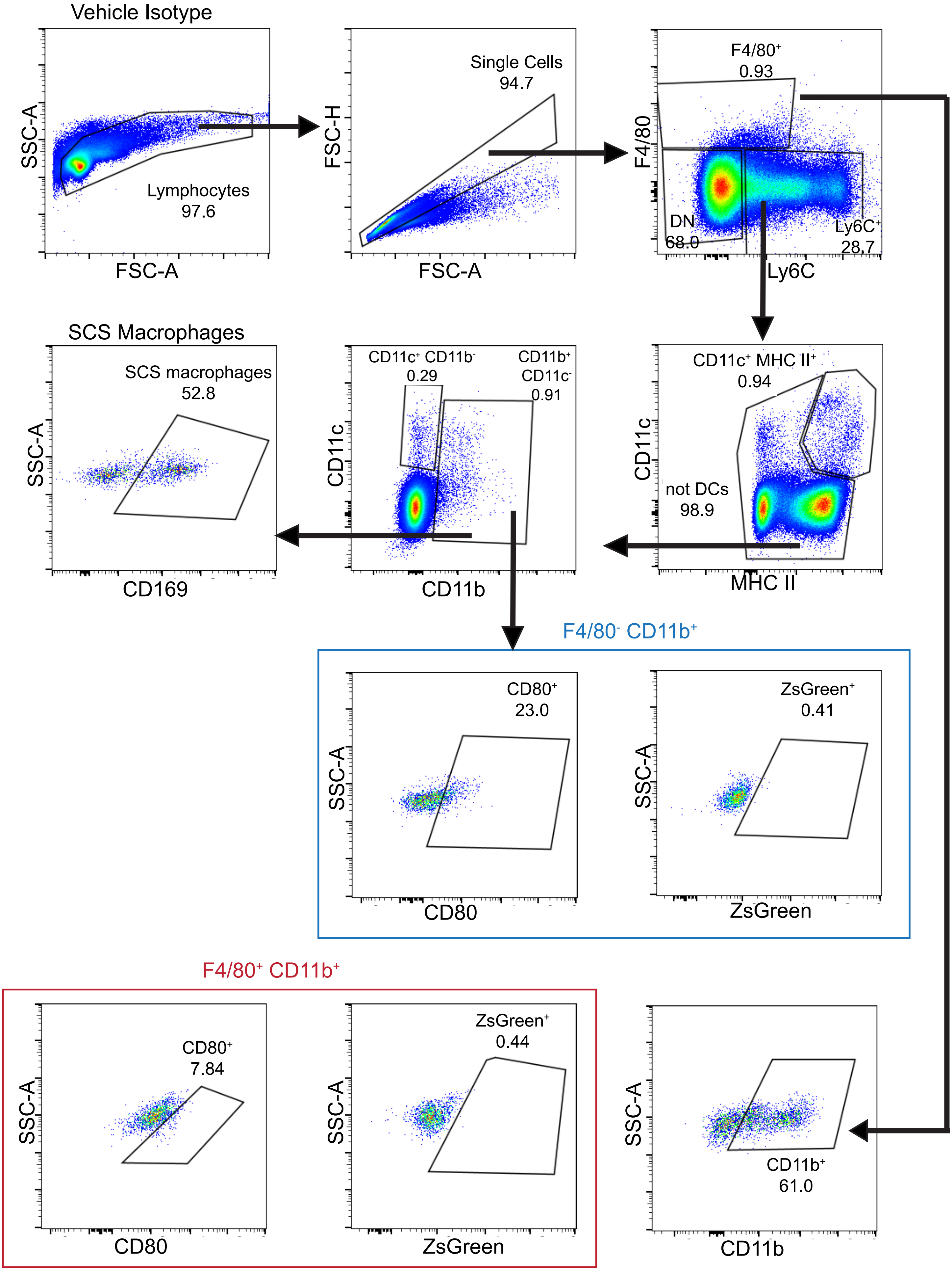

Representative flow plots showing gating on macrophage populations (quantified in Figure S13). WT mice were inoculated with bilateral subcutaneous 6694c2 tumors. Seven days later, one tumor was injected with STING agonist or vehicle, and mice were treated intraperitoneally with anti-PD-1 and anti-CTLA-4 or vehicle. Two days later, tumor-draining lymph nodes were harvested, digested, and analyzed via flow cytometry. Related to Figure 3 and Figure S7.

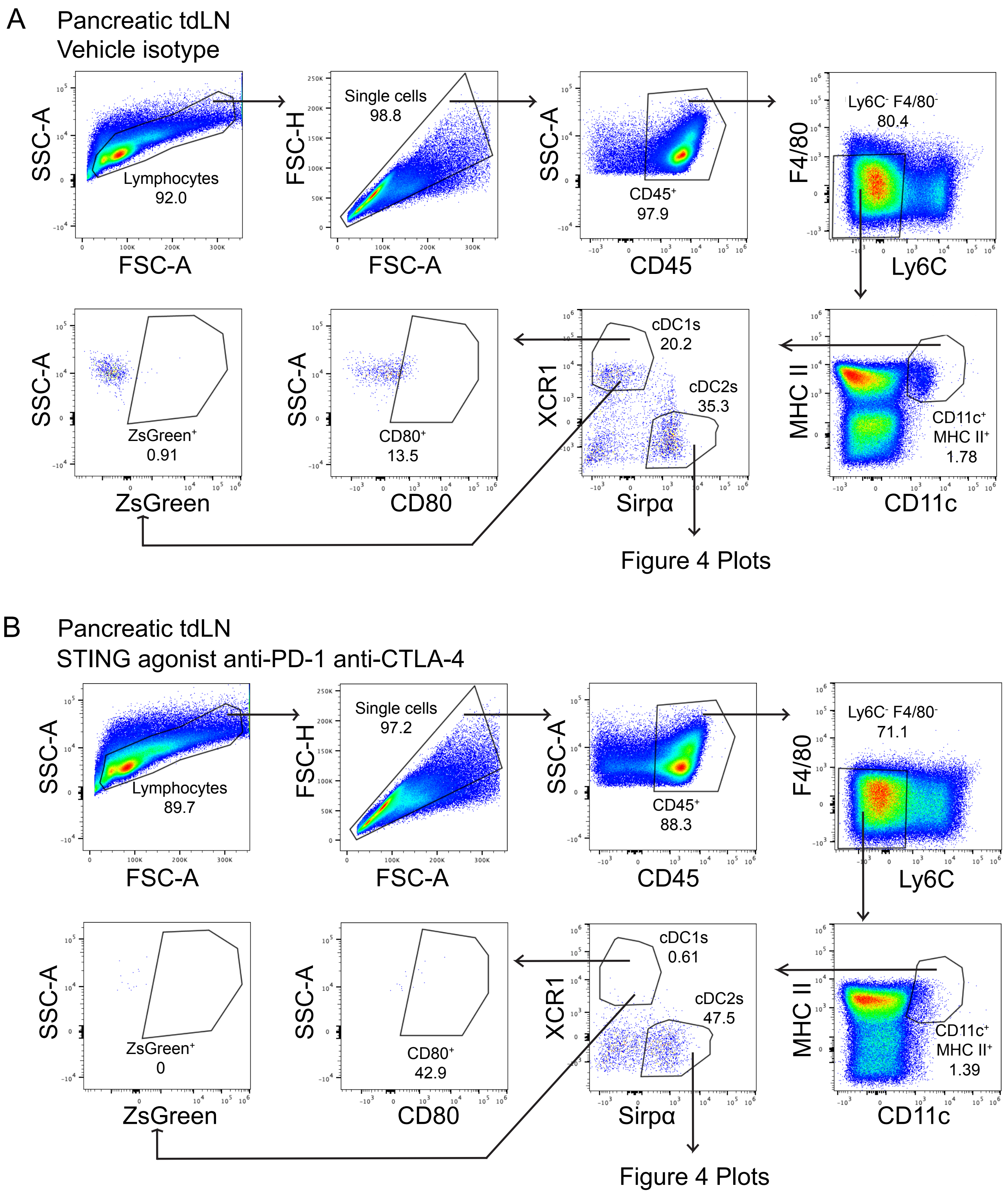

Dendritic cell subsets are found in pancreatic tumor-draining lymph nodes, related to Figure 4. Representative flow plots of pancreatic tumor-draining lymph node dendritic cell populations from (A) vehicle isotype-treated mice and (B) STING agonist anti-PD-1 anti-CTLA-4-treated mice quantified/shown in Figure 4G-I. Lymph nodes were harvested two days post triple combination therapy (day 9 post-inoculation), digested, and analyzed via flow cytometry.

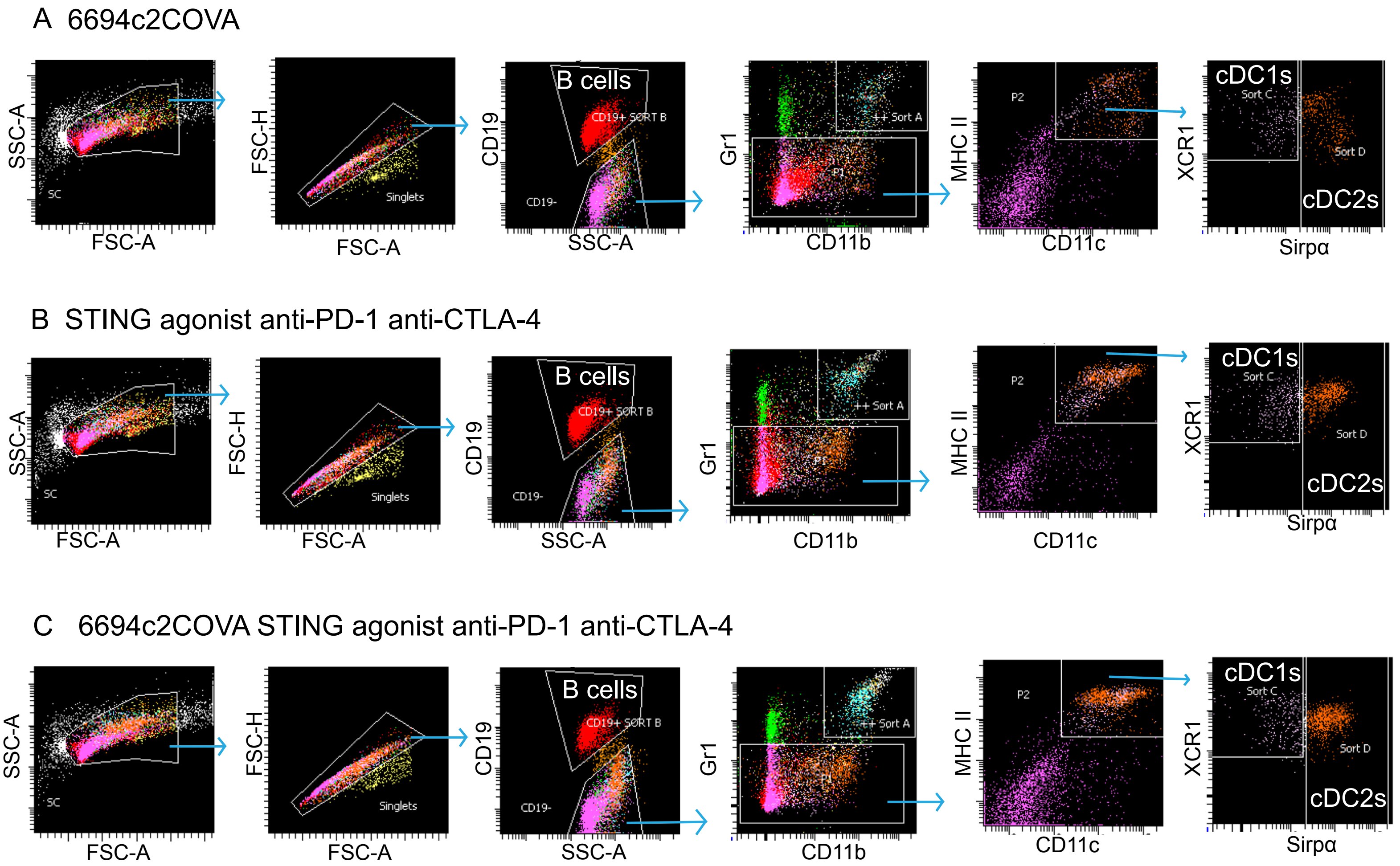

Ex vivo antigen presentation coculture sort gates, related to Figure 5. Representative flow plots from the experiment shown in Figure 5A-E. Mice were inoculated with 6694c2COVA tumors. 13 days post-inoculation, tumors were injected with STING agonist or vehicle and mice received intraperitoneal anti-PD-1 anti-CTLA-4 or isotype, respectively. Two days later, tumor-draining lymph nodes were harvested and digested. After staining, antigen-presenting cells were sorted from lymph nodes (sort gates shown).

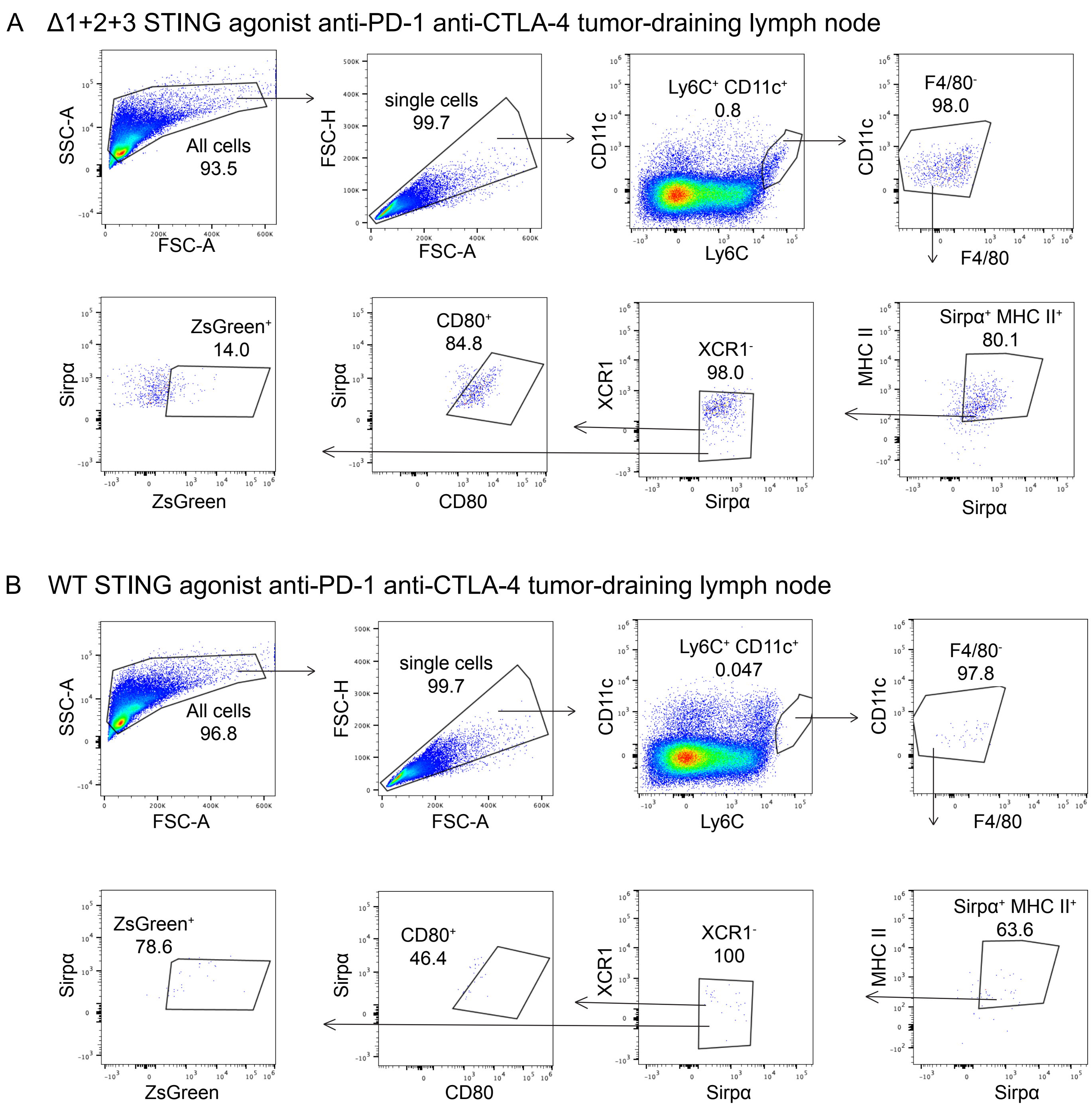

Flow cytometry gating of Ly6C+ cells, related to Figure S9. Representative flow cytometry plots of tumor-draining lymph node cells from (A) Δ1+2+3 and (B) WT mice 48 hours after treatment with STING agonist anti-PD-1 and anti-CTLA-4.

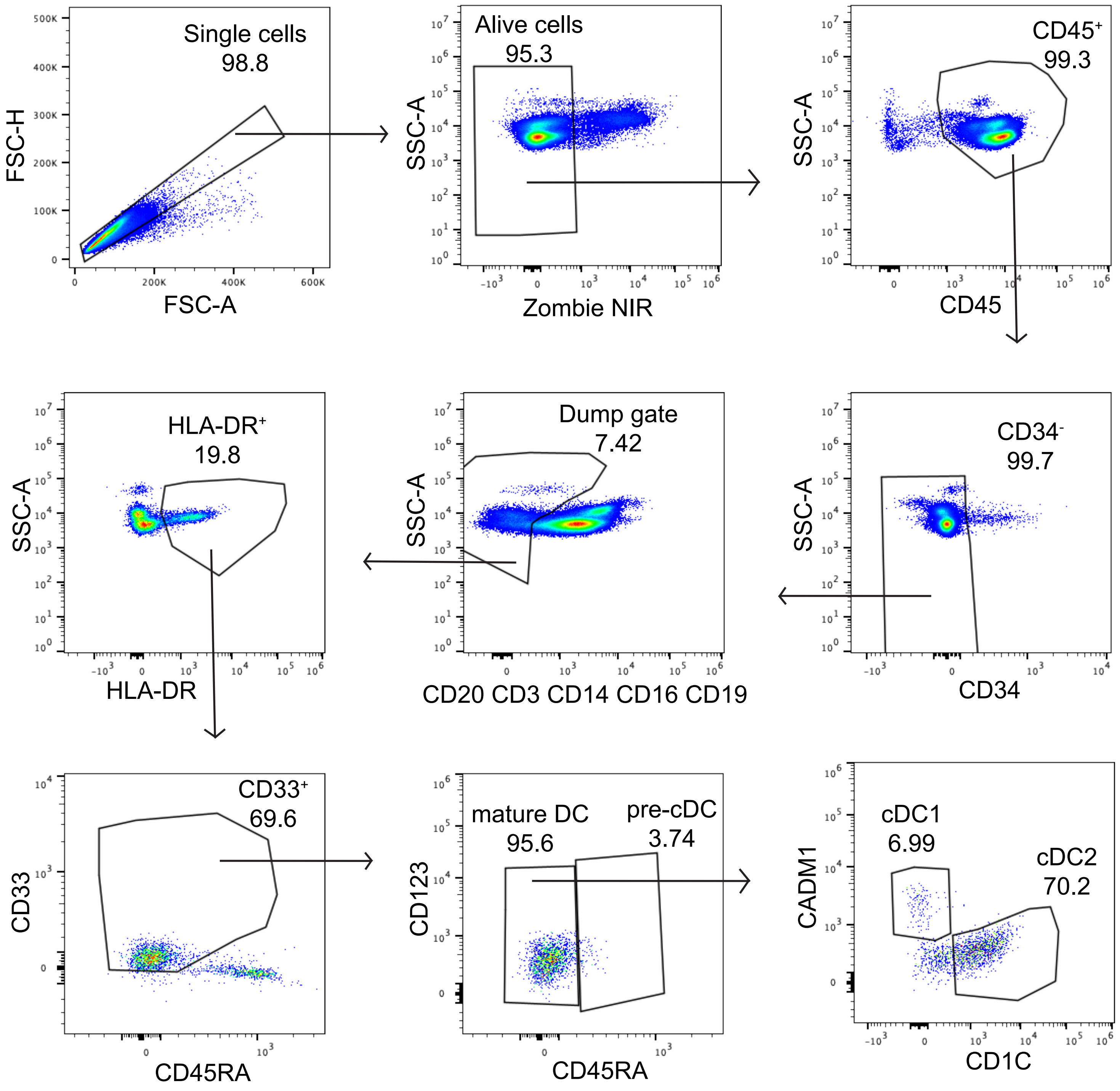

Human dendritic cell gating, related to Figure 7. Representative flow plots showing flow cytometry gating for Figure 7K. Blood samples were taken from patients with PDAC. Samples were stained for flow cytometry analysis.
